# Harnessing glucose metabolism with nanomedicine for cancer treatment

**DOI:** 10.7150/thno.100036

**Published:** 2024-10-17

**Authors:** Xudong Wang, Liping Wang, Qingyi Hao, Meng Cai, Xueting Wang, Wenlin An

**Affiliations:** 1 National Vaccine & Serum Institute (NVSI), China National Biotech Group (CNBG), Sinopharm Group, No. 38 Jing Hai Second Road, Beijing 101111, China.; 2 School of Life Science and Technology, China Pharmaceutical University, Nanjing, 211195, China.; 3 China National Pharmaceutical Group Co Ltd., Sinopharm Plaza, No 20 Zhichun Road, Haidian district, Beijing 100191, China.; 4 Department of Respiratory and Critical Care Medicine, The Affiliated Jiangning Hospital of Nanjing Medical University, Nanjing 211100, China.

**Keywords:** nanomedicine, glucose metabolism, cancer therapy, Warburg effect, glycolysis

## Abstract

The significance of metabolic processes in cancer biology has garnered substantial attention, as they are essential for meeting the anabolic demands and maintaining the redox balance of rapidly dividing cancer cells. A distinctive feature of tumors is that cancer cells, unlike normal cells, exhibit an increased rate of glucose metabolism. They predominantly relying on aerobic glycolysis to metabolize glucose, which enables these cells to supply energy and produce the necessary building blocks for growth. Targeting glucose metabolism has led to the development of various cancer treatments. However, these agents often have limited efficacy due to factors such as poor stability and solubility, rapid clearance and an insufficient amount of the drug reaching the target site. These limitations can be overcome by preparing nano dosage forms through nanotechnology, which leverages the unique properties of nanomaterials to deliver drugs more precisely to target tissues with controlled release. In this review, we provide a comprehensive overview of the latest advancements in nanomedicine, focusing on the modulation of glucose metabolism in cancer cells. We discuss the design and application of various strategies that have been engineered to target the metabolic hallmarks of cancer. These nanomedicine strategies aim to exploit the metabolic vulnerabilities of cancer cells, thereby offering novel approaches to cancer therapy. The review highlights the innovative nanomaterials and their potential to deliver therapeutic agents more effectively, as well as the challenges and considerations in translating these nanomedicines from bench to bedside. By targeting the glucose metabolism of cancer cells, these nanoscale interventions hold promise for improving treatment outcomes and potentially overcoming the resistance that often plagues conventional cancer therapies.

## 1. Introduction

Cancer cells undergo metabolic alterations that enable them to efficiently utilize glucose to support their high energy and biosynthetic requirements 1. Cancer cells frequently display the Warburg effect, a metabolic hallmark where they opt for aerobic glycolysis to generate energy and metabolic intermediates, regardless of the availability of oxygen 2. This metabolic shift not only supplies the building blocks essential for their growth and proliferation but also helps them maintain a delicate redox balance crucial for survival. Beyond glycolysis, cancer cells showcase metabolic plasticity, harnessing the power of mitochondrial oxidative phosphorylation (OXPHOS) to produce ATP with inherently necessitates a continuous oxygen supply 3. This adaptability in their metabolic pathways allows them to thrive in challenging conditions, such as hypoxic and nutrient-deprived environments, which are common in the tumor microenvironment (TME), limiting the efficacy of therapies aimed at exploiting reactive oxygen species (ROS). Metabolic reprogramming in cancer cells is often a direct response to the TME, which can influence the activity of various metabolic pathways. Key events in these pathways include the pronounced Warburg effect, alterations in the Krebs cycle metabolites, and an increased rate of o OXPHOS. In addition, cancer cells often rely on OXPHOS as a critical mechanism for their survival and proliferation, with cancer stem cells (CSCs) demonstrating an intensified reliance on this pathway 4. This pronounced dependency is commonly seen in instances of inherent or acquired resistance to chemotherapy and tyrosine kinase inhibitors 5. CSCs play a significant role in tumor metastasis and resistance to standard therapeutic strategies. Together, these metabolic adaptations fuel the relentless energy demands of cancer cells, supporting their relentless growth, invasion, and ability to withstand the harsh conditions within the tumor microenvironment. Given their significance in the glucose metabolism of cancer cells, these transporters and enzymes present themselves as potential molecular targets for cancer therapy 6. Hence, inhibiting their function could disrupt the metabolic pathways that cancer cells rely on for growth and survival, offering a promising strategy for the development of new cancer treatments.

Nevertheless, the instability and poor solubility of drugs, rapid clearance and metabolism rates, and nonspecific targeting issues led to limited therapeutic outcomes. The application of nanomaterials in the field of drug delivery has revolutionized the way various therapeutic agents are transported to target sites within the body 7. Leveraging their high specific surface area and adjustable size, nanocarriers have been engineered to effectively deliver a broad spectrum of therapeutic payloads, including small molecules, nucleic acids, and proteins 8. One of the significant advantages of using nanomaterials as drug carriers is their ability to address several inherent challenges associated with conventional drug delivery systems 9. The instability and poor solubility of drugs, rapid clearance and metabolism rates, and nonspecific targeting issues can be mitigated by encapsulating the drugs within nanocarriers 10. This encapsulation not only protects the therapeutic agents from degradation but also enhances their solubility and circulation time in the body. Nanomedicine, particularly, has demonstrated its prowess in enriching at the site of solid tumors due to the unique phenomenon known as the enhanced permeability and retention (EPR) effect 11. This effect allows nanoparticles to preferentially accumulate in tumor tissues, which are more permeable due to their abnormal vasculature. Nanomedicine offers a promising avenue for targeting glucose metabolism in cancer cells as a strategy to overcome resistance and improve treatment outcomes. By designing nanocarriers that can specifically modulate glucose metabolism within cancer cells, it may be possible to disrupt their energy supply and induce cell death, thereby enhancing the effectiveness of cancer therapy.

**Scheme 1:**
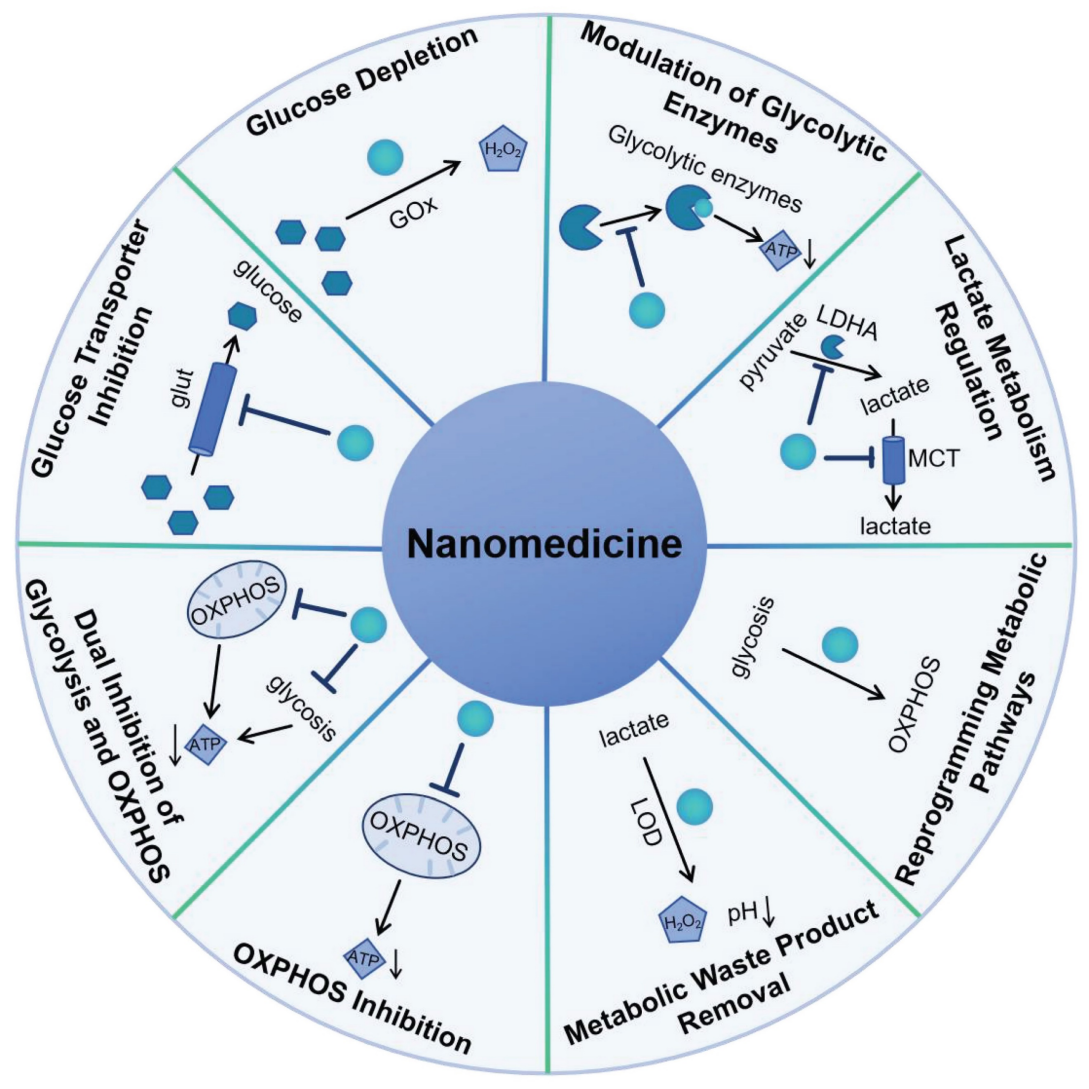
Schematic representation of nanomedicine approaches that exploit glucose metabolism pathways to treat cancer.

In this review, the latest advancements in nanomedicine that focus on targeting the glucose metabolism of cancer cells was summarized according to the mechanism of nanomedicine to regulate glucose metabolism including glucose transporter inhibition, glucose depletion by glucose oxidase, modulation of glycolytic enzymes, lactate metabolism regulation, reprogramming metabolic pathways, metabolic waste product removal, OXPHOS inhibition and combination therapy of inhibition of glycolysis and OXPHOS (Scheme 1). It highlights the innovative approaches and novel nanomaterials being developed and tested for their potential to improve cancer treatment by exploiting the unique metabolic vulnerabilities of cancer cells.

## 2. Glucose Metabolism in Cancer

**Figure 1:**
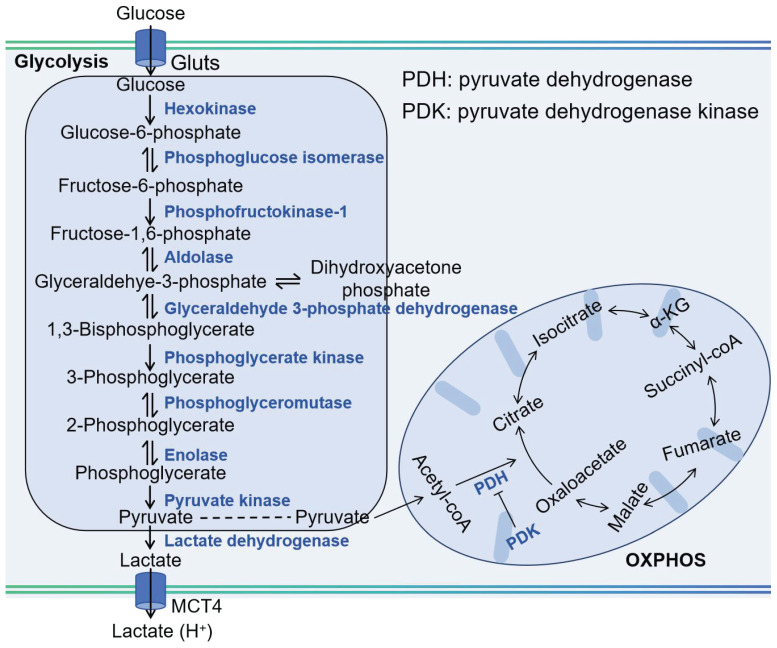
Glycolysis and OXPHOS in tumor cells.

Glucose metabolism in cancer is a critical process that supports the rapid proliferation and survival of tumor cells. Cancer cells often exhibit a phenomenon known as the Warburg effect, where they preferentially utilize aerobic glycolysis to produce energy and metabolic intermediates, even in the presence of oxygen 12. This altered metabolism provides cancer cells with the necessary building blocks for growth and division, as well as a means to maintain redox balance. As shown in Figure 1, the initial phase of glucose metabolism in cancer cells is facilitated by an elevated glucose intake, achieved through the overexpression of glucose transporters (Glut), such as Glut1 13. This increased glucose availability is crucial for the first committed step of glycolysis, where hexokinase enzymes phosphorylate glucose to glucose-6-phosphate (G6P). In cancer cells, the expression of hexokinase 2 (HK2) is induced alongside the hexokinase 1 (HK1) that is also present in normal cells, thereby doubling the capacity for this critical step. Following the initial phosphorylation, the second committed step in glycolysis is catalyzed by phosphofructokinase 1 (PFK1), which converts fructose-6-phosphate (F6P) into fructose-1,6-bisphosphate (F1,6BP). This reaction is significant as it is a key regulatory point in glycolysis, controlling the flux of metabolites through the pathway. The third committed step is mediated by pyruvate kinases, which transform phosphoenolpyruvate (PEP) into pyruvate. This conversion is another critical regulatory point, as it marks the final step in the glycolytic pathway leading to the production of adenosine triphosphate (ATP). The end product, pyruvate, is then reduced to lactate by lactate dehydrogenase (LDH), which simultaneously generates nicotinamide adenine dinucleotide (NAD^+^) from nicotinamide adenine dinucleotide (NADH). The regenerated NAD^+^ is essential for sustaining glycolysis, as it is required for the reduction of pyruvate to lactate. The lactate produced is then secreted by the cancer cell via monocarboxylate transporters (MCT), which facilitate its exchange across the cell membrane. The overexpression of Glut1, HK2, PFK1, lactate dehydrogenase A (LDHA), and MCT1, 4 is observed in a multitude of tumor types, highlighting their role in cancer metabolism 14. Limiting supplying nutrients to the cell and impair bioenergetics could prevent an adaptive response to cell stress.

Cancer cells exhibit a metabolic versatility that goes beyond glycolysis, as they can also generate ATP through OXPHOS. Contrary to the common belief that OXPHOS is universally reduced in cancer due to the Warburg effect, recent research indicates that certain cancers, such as leukemias, lymphomas, pancreatic ductal adenocarcinomas, certain melanomas, and endometrial carcinomas, can have an upregulated OXPHOS pathway, even when glycolysis is active 3. Cancer stem cells, a subset of cells within tumors known for their self-renewal and tumor-initiating capabilities, are also characterized by an upregulated OXPHOS mechanism 15. This heightened metabolic activity supports their resistance to therapies and their potential to drive tumor recurrence and metastasis. The OXPHOS process involves the electron transport chain (ETC) in the mitochondrial inner membrane, where nicotinamide adenine dinucleotide (NADH), reduced flavin adenine dinucleotide (FADH2), and succinate donate electrons to a series of protein complexes (I to IV). These complexes pump protons into the intermembrane space, creating a gradient. The protons then flow back through ATP synthase (complex V), synthesizing ATP in the process, with oxygen serving as the final electron acceptor. The use of OXPHOS inhibitors is a novel strategy, either to directly treat cancers with elevated OXPHOS or to mitigate tumor hypoxia, thereby enhancing the effectiveness of other treatments.

Thus, targeting glucose metabolic pathways emerges as promising strategy to impede tumor growth effectively. There has been a resurgence in the quest to leverage metabolic enzymes as therapeutic targets for cancer, yet the array of molecules that are specifically targeting glucose metabolism in clinical trials remains sparse (Table 1). Despite this, a growing corpus of evidence is lending credence to the potential of several metabolic enzymes as viable targets. Studies employing tool compounds have begun to unveil encouraging results within the realm of preclinical cancer research. In the foreseeable future, it is expected that an influx of innovative molecules will be directed toward these metabolic enzymes and will make their way into clinical trials. A comprehensive summary of the enzyme targets that are relevant to both categories is provided in Table 1. However, the therapeutic impact of drugs designed to target glucose metabolism is often curtailed by the challenges of off-target toxicity and the adverse effects associated with high dosages. This reality underscores the pivotal role that nanodelivery strategies play in enhancing drug efficacy, diminishing toxicity, and ameliorating the deficiencies in the application of these therapeutic agents.

## 3. Regulation of Glucose metabolism in Clinical Practice

In clinical practice, modulating glucose metabolism in cancer stands as a pivotal and burgeoning field in both research and therapeutic development. The advent of medications that exploit the metabolic frailties of cancer cells, especially those within energy and redox metabolic pathways, has significantly impacted cancer treatment strategies. Lonidamine, an indazole-3-carboxylic acid derivative, exemplifies such an approach, demonstrating its utility in clinical trials for solid tumors such as breast, ovarian, and lung cancers. Its mechanism of action involves the significant disruption of cancer cell energy metabolism, including the inhibition of oxygen consumption and the accumulation of lactate. While lonidamine's efficacy as a single agent is limited, its synergistic use with chemotherapy has shown promising results, enhancing the therapeutic index in various cancers 39.

The use of 2-deoxy-D-glucose (2DG) in culturing peripheral blood mononuclear cells (PBMCs) has also revealed its potential in modulating immune responses to cancer. PBMCs cultured with 2DG prior to microwave ablation have exhibited an increase in CD8^+^ central memory T cells and CD39^+^ CD8^+^ TCM, suggesting an enhancement of the immune system's memory response to malignancies. TLN-232, a novel peptide, is another therapeutic candidate that targets pyruvate kinase M2 (PKM2), a protein overexpressed in various tumors and linked to the Warburg effect 40. By inducing the translocation of PKM2 into the nucleus, TLN-232 triggers cancer cell death. Early-phase clinical trials in advanced renal cell carcinoma have shown that TLN-232 is well-tolerated, with some patients exhibiting stable disease. Dichloroacetate (DCA) has demonstrated the ability to target mitochondrial respiration of cancer cells, thereby impairing their survival and progression. Clinically, DCA is administered orally or parenterally, with dosages ranging from 10 to 50 mg/Kg/day. It has shown a favorable safety profile, although common side effects include gastrointestinal issues and peripheral neuropathy, which may be mitigated by co-administration of antioxidants 41. Overall, the clinical manipulation of glucose metabolism in cancer represents a swiftly progressing field that necessitates ongoing research to overcome current therapeutic challenges and to pioneer more efficacious treatment methods.

## 4. Principles of Nanomedicine Design

Nanotechnology has been harnessed to enhance the precision, stability, and bioavailability of pharmaceuticals, ensuring their targeted delivery to specific bodily sites and cells 42. Historically, drugs have faced challenges such as instability, low solubility, rapid clearance, swift metabolism, and nonspecific targeting. Nanoencapsulation addresses these issues by shielding therapeutic agents from degradation and enhancing their solubility, thereby achieving efficient delivery to disease sites while minimizing off-target effects. An effective nanocarrier must meet a range of design specifications, including high drug loading capacity, triggered or timed-release mechanisms, optimized circulation (stealth properties), stability in serum or plasma, non-toxicity, targeting ability, lack of immunogenicity, cellular uptake, and avoidance of accumulation in non-target tissues. Efficacy and safety are paramount in assessing whether a novel nanocarrier is viable for practical application. The progress in surface modification, precise control over nanoparticle morphology, and advanced functionalization techniques have empowered nanomedicines to adeptly navigate the complex tumor microenvironment. These advancements ensure accurate targeting of tumor tissues while minimizing or even eliminating adverse safety effects. Recent breakthroughs in the field of nanomedicine have significantly improved the efficacy and safety of cancer treatments. Innovations in surface modification have not only enhanced the biocompatibility of nanoparticles but also their targeting efficiency, ensuring that therapeutic agents are delivered precisely to the tumor site. Moreover, the meticulous control over the morphology of nanoparticles, including their size and shape, has been instrumental in optimizing their penetration and distribution within the tumor microenvironment. This allows for a more effective engagement with cancer cells while sparing healthy tissues. Furthermore, the functionalization of nanoparticles has been a game-changer, endowing them with specific properties that fine-tune their therapeutic response. This sophisticated approach tailors the interaction between the nanomedicine and the tumor, leading to a more precise and potent attack on cancer cells while minimizing adverse effects. These collective advancements have set the stage for a new era in cancer therapy, where treatment is not only more effective but also safer for patients 43-47.

### 4.1 Surface Modification

The surface modification of nanomedicine involves the attachment of ligands that have a high affinity for receptors overexpressed on cancer cells. This targeted approach minimizes the adverse effects on healthy tissues and reduces systemic toxicity, which is a common issue with traditional chemotherapy.

Song *et al.* have shown that Anti-HIF-1α antibody-conjugated nanocarriers can effectively target and then been internalized by cancer cells where the antibody-conjugated nanocarriers release their encapsulated toxin and selectively kill cancer cells 48. Besides, Nie *et al.* assembled dibenzocyclooctyne-modified anti-CD47 and anti-SIRPα antibody-conjugated nonocarriers, which can actively target tumors through the specific recognition between anti-CD47 antibody and CD47 on the tumor cell surface. The antibody-conjugated nonocarriers then abolish “don't eat me” signaling and improve phagocytosis of macrophages within acidic tumor microenvironment 49. In addition, studies demonstrated elevated tumor penetration efficacy by modified nanocarriers with peptide 50, featuring high-targeting profile, facile preparation, and excellent biocompatibility. Furthermore, aptamer 51 and small molecule 52 conjugated nanocarriers were reported have enhanced tumor targeting, permeability, and retention effects.

### 4.2 Nanoparticles morphology control

One pivotal aspect of nanoparticle design that significantly influences both pharmacokinetics and cellular uptake is the morphology of the nanoparticles, encompassing both their size and shape. The optimal size for evading clearance and accumulating in tumor tissues through the EPR effect is often cited as being around 10-200 nm. The shape of nanoparticles also plays a critical role in their interaction with biological systems. Nanoparticle morphology can directly affect their surface area and surface chemistry, which in turn dictate the binding affinity to biological targets and the subsequent internalization mechanisms, such as phagocytosis, macropinocytosis, or clathrin-mediated endocytosis. The shape can also influence the release profile of encapsulated drugs, with certain shapes allowing for more controlled or triggered release compared to others.

#### 4.2.1 Size Control

Size control is another critical aspect of nanomedicine design. Nanoparticles within a certain size range (typically 10-200 nm) can preferentially accumulate in tumor tissues due to the EPR effect 53. This size-dependent selective delivery is a key advantage of nanomedicine, as it allows for a higher concentration of the drug to reach the tumor while avoiding rapid clearance by the immune system or the kidneys. Computational modeling can help predict the effect of nanoparticle size on the motion, margination, and nonspecific or specific adhesion of particles in a hemodynamic flow. For example, Gentile *et al.* investigated the behavior of nanoparticles of different diameters (spherical particles 50, 100, 200, 500, and 750 ​nm in diameter, 1, 6, and 10 μm) in dynamic flows 54. Soltani used pH-responsive nanocarriers to examine the impacts of hypoxic regions as well as the size of nanocarriers for cancerous cell-death. Results show that nanocarriers with smaller sizes are more effective due to higher accumulation in the tumor tissue interstitial. The small size of the nanocarriers also allows them to penetrate deeper, so they can expose a larger portion of the tumor to the drug 55.

#### 4.2.2 Shape Control

The configuration of nanoparticles plays a crucial role in their *in vivo* behavior, including circulation longevity, flow properties, tumor accumulation, cellular internalization, and the ability to penetrate tumor tissues. Research by Zhao and team has illustrated that, after oral ingestion, rod-shaped nanoparticles linger longer in the gastrointestinal tract than spherical ones 56. Long rod nanoparticles (NLR) showed an enhanced capacity to avoid swift removal by the reticuloendothelial system, leading to an extended presence in the bloodstream when compared to short rod nanoparticles (NSR) and spherical nanoparticles (NS). Spherical nanoparticles were found to be eliminated more quickly than the rod-shaped ones. Moreover, silica nanoparticles exhibit shorter rods (NSR) degrading faster than their longer (NLR) and spherical (NS) counterparts, potentially due to increased surface reactivity. LSR provided superior bioavailability compared to those loaded into short rods and spheres. The strategic transformation of nanoparticle shape is pivotal for optimizing retention times, ensuring not only their efficient circulation and extravasation but, more critically, enhancing their accumulation, retention, and penetration within tumor tissues. This shape-mediated approach is essential for maximizing therapeutic efficacy by ensuring that nanoparticles can effectively target and infiltrate the TME.

### 4.3 Functionalization

Functionalization of nanomedicine refers to the incorporation of additional features that can enhance the therapeutic effect or improve the delivery of the drug. For example, pH-sensitive nanocarriers can release their payload in the slightly acidic environment of the tumor 57, while thermo-responsive nanoparticles can be triggered to release drugs upon exposure to mild heat in the presence of nanoparticles which could induce an increase of temperature 58. The use of nanocarriers in drug delivery offers a multifaceted solution to common issues in pharmaceuticals, including improved stability, enhanced solubility, prolonged circulation, targeted delivery, and stimulus-responsive release, all of which contribute to a more effective and safer therapeutic approach. For example, inorganic nanocarriers could functionalized with peptide, proteins, DNA and RNA, enabling targeted delivery and controlled release of therapeutic agents 59. Biological nanocarriers, such as virus-like particles (VLPs), exosomes, cell membrane camouflaged nanocarriers, and outer membrane vesicles be engineered to encapsulate a variety of cargoes, including nucleic acids, proteins, and small molecules, and can be produced on a large scale for applications in vaccine development, gene therapy, and drug delivery. The simple and modular composition of VLPs allows for easy modification and functionalization, making them an attractive platform for the development of targeted and controlled release systems 60. Cheng and colleagues have ingeniously combined the genetic code expansion technique with synthetic biology strategies to achieve site-specific modification of VLPs for the display of exogenous tumor antigens while mitigating preexisting immunity 61. Through a process of modification site screening, hepatitis B core VLPs which incorporated with azido-phenylalanine at the primary immune region, could efficiently assemble and swiftly conjugate with dibenzocyclooctaline-modified tumor-associated antigens, specifically mucin-1. Membrane derived from cells or bio-vesicles of different types could be employed to coat particles to alter their surface property. By utilizing the nature of their shell membrane, the property of these nanoparticles can be improved including excellent biocompatible, prolonged circulation as well as targeting 62. Many types of membranes have been used to construct biomimetic core-shell nanoparticles for cancer therapy, including membranes from red-blood-cells, platelets, bacteria, white-blood cells, cancer cells, stem cells.

Although the longevity, targeting ligands, and stimuli-responsive moieties of nanomedicines significantly increased the drug disposition in the tumor area, insufficient cellular internalization of nanomedicines can be another significant barrier, especially for macromolecular drugs such as proteins or nucleic acids. To achieving precise delivery, the introduction of multifunctional nanocarriers could encapsulate drug combination for the improvement of therapy outcome. For instance, Wu and colleagues have developed a lipid-coated mesoporous silica nanoparticle (MSN) system for the co-delivery of cisplatin and tirofiban, an antiplatelet agent, aimed at disrupting lymphatic vessel invasion (LVI) formation and simultaneously boosting the antitumor efficacy of cisplatin 63. To augment tumor-targeting specificity, the nanodrug was functionalized with a cyclic peptide, Cys-Arg-Glu-Lys-Ala (CREKA). This tailored nanodrug is capable of preventing LVI formation while also enhancing the chemotherapeutic potency of cisplatin, all without incurring significant adverse effects, offering a multifaceted therapeutic approach to cancer treatment. The advancement of functionalized nanoparticles enables cancer therapy to evolve into more precise modalities, offering enhanced treatment options that are tailored to the specific characteristics of tumors.

## 5. Strategies to Improve Tumor Targeting of Nanomedicine

Nanomedicine, an interdisciplinary field that combines nanotechnology and medicine, has shown great promise in improving the targeting of therapeutic agents to tumor sites. The main goal is to enhance the efficacy of treatments while minimizing side effects on healthy tissues. Here are several strategies to improve tumor targeting of nanomedicine:

### 5.1 Passive Targeting

Tumors frequently exhibit characteristics such as leaky blood vessels and deficient lymphatic drainage systems, which can lead to a preferential accumulation of nanoparticles at the tumor site due to their size. This phenomenon is known as the EPR effect, and it can be strategically utilized to passively direct nanoparticles towards tumors 64. Optimizing the size (typically ranging from 10 to 200 nm) and surface characteristics of nanoparticles can significantly improve their concentration at the tumor site through the EPR effect. The efficacy of therapeutics relying on the EPR effect can be influenced by the shape and surface charge of the nanocarrier, as these factors can alter the mode of transport, enhance stability, and improve intracellular penetration.

Non-spherical nanocarriers, for instance, may exhibit greater diffusivity compared to their spherical counterparts 65. This is because they can interact more with the vessel walls, potentially due to an increased radial thrust force resulting from rapid pressure fluctuations. Studies have shown that rod-shaped nanocarriers can be retained up to four times more in solid tumors than spherical ones 66. Additionally, nanocarriers with a positive surface charge have been observed to accumulate more at the tumor site than those with a negative charge 67. However, positively charged nanocarriers can also present higher toxicity to non-target tissues and are more rapidly cleared from the body, which can limit their therapeutic potential.

To overcome these limitations, surface modification techniques for nanocarriers have been developed. These modifications allow the nanocarriers to assume a positive charge within the tumor environment, thus mitigating the drawbacks associated with non-target tissue toxicity and rapid clearance. The incorporation of polyethylene glycol (PEG) modification has been instrumental in augmenting the systemic circulation time of nanosystems 68. This strategy is pivotal in surmounting the formidable barrier of immune system clearance, which frequently impedes the effective delivery of nanoparticles. Utilizing membranes that can sequester cell surface antigens, nanosystems was engineered with superior biocompatibility, thereby minimizing their recognition and clearance by the immune system. For example, CD47 on red blood cell membrane which serves as a “don't eat me” signal helps prevent macrophage uptake of nano vehicles 69. By employing such strategies, the development and application of nanocarriers in cancer therapy can be significantly enhanced, potentially leading to more effective treatments with reduced side effects.

### 5.2 Active Targeting

This strategy involves equipping nanocarriers with ligands that can specifically bind to overexpressed receptors on cancer cells 70. These ligands can be antibodies, peptides, or small molecules that recognize and bind to tumor-specific antigens, leading to selective uptake by cancer cells. By actively targeting cancer cells, the delivery of therapeutic agents can be significantly improved. The surface of nanoparticles can be modified with a variety of targeting ligands that possess specific recognition capabilities 71. When these ligands bind to their corresponding receptors or antigens on the surface of cancer cells, they enable the precise targeting of nanocarriers to tumors. This active targeting strategy leverages the overabundance of specific receptors on the cell membranes of tumor cells or the unique expression of certain protein receptors.

Active targeting using these biorecognition events is a strategic approach to improve the selectivity and efficacy of nanomedicine. By ensuring that the nanocarriers are preferentially taken up by cancer cells, this method aims to minimize off-target effects and increase the therapeutic index of the treatment. The use of targeting ligands that have a high affinity for molecules that are overexpressed in cancer cells provides a means to actively direct the nanocarriers to the tumor site, potentially revolutionizing the treatment of cancer with nanomedicine.

### 5.3 Tumor Microenvironment-Responsive Nanocarriers

The TME is characterized by unique features such as low pH, high temperature, and the presence of specific enzymes 72. Nanocarriers can be designed to respond to these environmental cues, releasing their payload in response to these triggers. The complex immunosuppressive network formed by stromal cells, inflammatory cells, the vascular system, extracellular matrix (ECM), immune-related cells, and their secreted cytokines in the TME plays a key role in tumor immune escape. By leveraging these features, an intelligent delivery system was implemented to specifically target tumor sites, thereby facilitating cancer therapy. This approach has led to significant therapeutic benefits while also minimizing side effects. TME selective therapy can be facilitated by various biomarkers in TME, including acidic pH levels indicative of the tumor microenvironment, elevated endogenous hydrogen peroxide (H_2_O_2_), overexpression of specific enzymes, hypoxia resulting from inadequate oxygen supply, and high levels of glutathione (GSH), among other factors 73. For example, pH-sensitive nanoparticles can release drugs in the slightly acidic TME, while enzyme-responsive nanoparticles can release their cargo upon cleavage by specific enzymes overexpressed in the TME.

## 6. Mechanism of Nanomedicine to Regulate Glucose Metabolism

The mechanism by which nanomedicine regulates glucose metabolism in cancer cells involves several strategic approaches such as direct inhibition of glucose uptake and glycolytic enzymes, glucose depletion, reprogramming of metabolic pathways, immune system modulation, waste product removal, and the integration of imaging and therapy. These strategies aim to exploit the metabolic vulnerabilities of cancer cells, offering a promising approach to enhance the efficacy of cancer treatment.

### 6.1 Targeting Glucose Transporter Inhibition

Nanomedicine can be designed to specifically target GLUTs that are overexpressed on the surface of cancer cells. By conjugating nanocarriers with molecules that can inhibit GLUTs, such as competitive inhibitors or GLUT antagonists, the influx of glucose into cancer cells is reduced. This limitation of glucose supply can impair the glycolytic pathway, thereby disrupting the energy production and biosynthesis processes that cancer cells rely on for growth and survival.

A significant characteristic of tumor was that cancer cell could utilize glucose to generate ATP via glycolysis, accompanied by the production of lactate. It has been found that the glut1 expression was higher in tumors in comparison with normal tissue from patients, demonstrating the increase of glucose uptake. While more glucose was applied to synthesis ribose, glycosylation precursors, amino acids, as well as lipids. As a consequence, glucose deprivation was considered as a therapeutic strategy for the treatment of cancer. To date, a variety of drugs that target tumor cell energy metabolism including glycolysis and OXPHOS was employed as anticancer agents, which has been approved or on trial. These drugs were selectively target on specific transporters (glucose transporters and monocarboxylate transporters) and enzymes including, hexokinase, 6phosphofructo 2kinasefructose2,6biphosphatase 3 (PFKFB3), pyruvate kinase isozyme M2 (PKM2), LDHA. In addition, glucose which was metabolized by pentose phosphate pathway (PPP) could provide NADPH to maintain cell redox homeostasis. Glucose transporters act as a passive energy independent carrier that transport glucose from extracellular into intracellular. Thus, gluts were considered as an effective therapeutic target for the treatment of cancer. Inhibition glut1 was a direct strategy to inhibit glucose uptake, enabling glucose across cancer cell membrane. Glut1 inhibitors contain cytochalasin B, WZB117, STF-31, BAY-876, siRNA and ASO. Several drugs inhibiting glut1 has been tested clinically.

**Figure 2:**
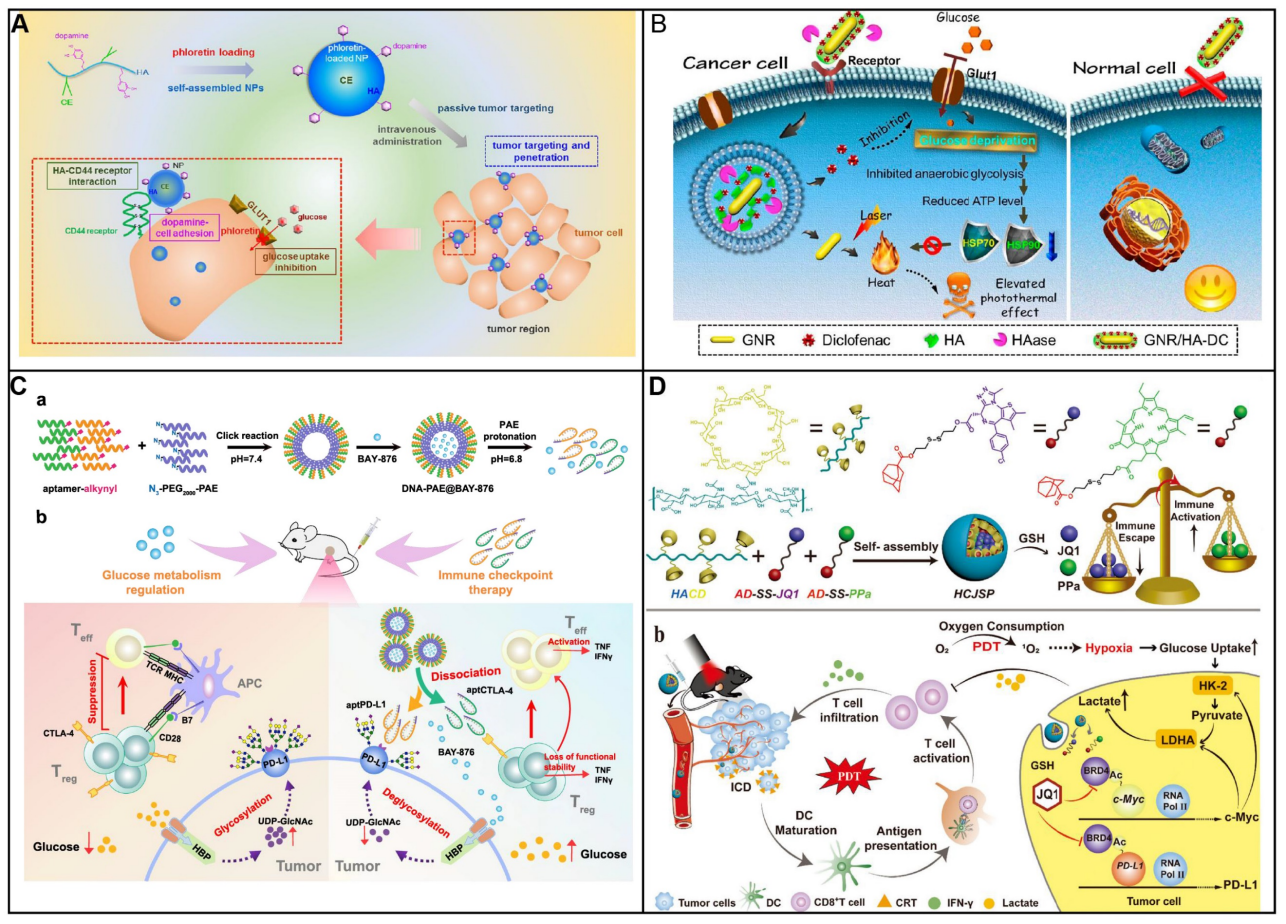
Targeting glucose transporter inhibition. (A) Schematic diagram showing tumor targeting and penetration strategy of HACE-d/phloretin NPs. Reproduced with permission from ref 74. Copyright 2017 American Chemical Society. (B) Schematic diagram depicting GNR/HA-DC for selectively sensitizing tumor cells to photothermal therapy by interfering with the anaerobic glycolysis metabolism. Reproduced with permission from ref 75. Copyright 2017 American Chemical Society. (C) Schematic diagram showing DNA-PAE@BAY-876 remodel TME, unleashing potent immunostimulatory responses to combat cancer progression. Reproduced with permission from ref 78. Copyright 2023 Nature. (D) Schematic diagram showing HCJSP prodrug nanoparticle and proposed mechanisms of HCJSP-based combinatory immunotherapy of pancreatic tumor by eliciting immunogenicity and overcoming adaptive immune resistance. Reproduced with permission from ref 79. Copyright 2021 Wiley-VCH.

Phloretin is rich in apples and could inhibit glucose uptake through inhibiting GLUT1. In order to enhance tumor targeting as well as penetration, Lee and coworkers have developed a nanosystem based on amphiphilic hyaluronic acid-ceramide-dopamine conjugate to delivery GLUT1 inhibitor phloretin (Figure 2A) 74. The dopamine which has mussel-inspired property in the nanosystem could be used to enhance cellular adhesion. The nanosystem was capable of targeting and penetration into tumor because high binding capacity as well as cellular adhesion property. The nanosystem exhibited high inhibition toward tumor in an MDA-MB-231 spheroid model. Furthermore, the nanosystem were more distributed in tumor tissue, demonstrating high specificity toward cancer. Compared with HACE NPs group, the nanosystem could infiltrate into the inside tumor. Therefore, the antitumor efficacy was improved by taking advantage of mussel-inspired delivery system.

By inhibiting anaerobic glycolysis, antitumor capacity of photothermal therapy (PTT) was augmented by decreasing the expression of heat shock proteins (HSPs), which was as the natural defense of cell to heat stress, leading to the thermal resistance in PTT in cancer therapy. To diminishes the thermal resistance of the targeting cell for an enhanced hyperthermal therapeutic efficiency, Chen and his colleagues have decorated the gold nanorod (GNR) with diclofenac (DC) as the adjuvant for enhancing the photothermal therapeutic efficiency (Figure 2B) 75. To enhance the tumor targeting of prepared nanomedicine, hyaluronic acid (HA) was conjugated on its surface. Once nanomedicine reaches cancer cells, highly expressed hyaluronidase (HAase) degraded HA, resulting the release of DC to reduce the level of Glut1. As a result, the glucose deprivation environment was formed in the cancer cell, and thus blocking anaerobic glycolysis, hampering ATP production and down-regulating HSP expression. As a consequence, cancer cells are more susceptive to PTT owing to glucose deprivation, therefore broadening the therapeutic window for PTT both *in vitro* and *in vivo*. By combining traditional therapeutic modalities (GNR) with glucose uptake inhibition by DC, this nanosystem exhibits an enhanced efficiency for hyperthermal therapy.

In addition, nanoparticle can be employed to load siRNA, shRNA as well as antisense oligonucleotide targeting GLUT-1 mRNA to silence the GLUT-1 expression, leading to the decrease of glucose uptake. Oliveira *et al.* have developed gold nanoparticles to delivery GLUT1 and GLUT3 antisense hairpin for downregulation glucose transporters of gastric cell 76. Although nanomedicine targeting GLUT1 is considered as a potential approach against cancer. Nevertheless, high expression of GLUT1 was also observed in a lot of normal tissue. Thus, there are severe side effects that GLUT1 serves as therapeutic target, limited its clinical application. GLUT3 is another transporter with high level in cancers. Especially, high expression level of GLUT3 was observed in brain tumor cells. Moreover, expression of GLUT3 is highest in brain tumor stem cells. When glucose expression is low in cancer, GLUT3 plays a more important role in tumor progression. In view of this, Wang group have developed a siRNA-based nanomedicine for the treatment of glioma via downregulation GLUT3 expression 77. In this study, cationic lipid-assisted nanoparticles were used to loaded siRNA with high drug encapsulation efficiency of 90%. The nanomedicine could knock GLUT3 expression in a glucose-poor microenvironment, and significantly decreased tumor cell proliferation. Moreover, the proportion of the glioma stem cell decreased owing to high expression of GLUT3 in glioma stem cell and improved the therapeutic effect.

An enhanced tumor-intrinsic PD-L1 glycosylation in triple-negative breast cancer (TNBC) was found due to its hyperglycolysis characteristic. This, strengthens the function of regulatory T cells (Tregs), consequently undermining the efficacy of immune-checkpoint inhibitors. The glycolytic activity in tumor cells produces D-fructose-6-phosphate, which is channeled into the hexosamine biosynthetic pathway (HBP) to generate UDP-GlcNAc, a key donor for protein glycosylation. Moreover, these hyperglycolytic tumor cells can outcompete immune cells in the TME for glucose, thereby reinforcing Treg immunosuppression and fostering tumor immunotolerance. Targeting TNBC glycolysis strategically could reshape the immunosuppressive TME and enhance the effectiveness of ICIs. Ren *et al.* introduced an aptamer-based nano assembly for TNBC treatment, integrating tumor cell-selective glycolysis inhibition with bispecific immune checkpoint blockade (Figure 2C) 78. The poly β-amino ester (PAE) was conjugated to PD-L1 and CTLA-4-antagonizing aptamers (aptPD-L1 and aptCTLA-4), which self-assembled into nanoparticles under neutral pH, increasing aptamer stability. The hydrophobic BAY-876 was encapsulated within the PAE core. In the acidic TME of TNBC, the PAE conjugates became protonated and hydrophilic, disrupting the nano assembly and releasing BAY-876, aptPD-L1, and aptCTLA-4. BAY-876 functioned to inhibit PD-L1 glycosylation, converting immunosuppressive Tregs into an immunostimulatory state, which amplified the therapeutic impact of the aptamers. The nanomedicine (DNA-PAE@BAY-876) treatment led to a significant increase in dendritic cells (DCs) and central memory T cells, along with an increase in proinflammatory cytokines IFN-γ and TNF-α by 33.65% and 30.87%, respectively, and a decrease in the anti-inflammatory IL-10 by 11.95%. This treatment induced a transformation of the immunosuppressive TNBC phenotype into an immune-activated one, evidenced by the enhanced infiltration and proliferation of CD8^+^ T cells, CD4^+^ T cells, DCs, and M1 macrophages at the tumor site. The DNA-PAE@BAY-876 nano assembly effectively halted TNBC growth, illustrating the potential of this multifaceted approach in overcoming the immunosuppressive challenges in TNBC treatment.

Furthermore, GLUT-1 level as well as its function can be regulated by various signal molecules including cAMP, c-Myc, Akt, p53, PI3k as well as HIF-1. Although immune checkpoint blockade therapy has attracted much attention for the treatment of solid cancer including head and neck squamous cell cancer, non-small cell lung cancer as well as melanoma. Notwithstanding, neither an anti-PD-1 nor anti-CTL4 treatment could be an effective clinical treatment for patients with pancreatic cancer either as a monotherapy or in combination. It is ascribed to its unique immunosuppressive tumor environment, that is, cytotoxic T lymphocytes cell deficiency but also a mass of Tregs, tumor-associated macrophages (TAMs) as well as myeloid-derived suppressive cells. Photoimmunotherapy was that photodynamic therapy induced immunogenic cell death of cancer cell to generate cell lysates. Then, the cell lysates including damage-associated molecular patterns (DAMPs) could promote immune response. However, photodynamic therapy could not only increase oxygen consumption but also destroy angiogenesis. This results in glucose metabolic reprogramming to facilitate the glycolysis of cancer cells, producing a large number of lactic acids, further inducing immunosuppressive environment. Sun and coworkers have developed a nanoplatform to delivery photosensitizer as well as a prodrug of JQ1 (Figure 2D) 79. The nanoplatform consists of three units: cyclodextrin-modified hyaluronic acid served as nanocarrier and targeting group, pyropheophorbide acts as photosensitizer and JQ1 was used to inhibit the expression of bromodomain-containing protein 4. As nanomedicine reaches at tumor site via EPR, the nanomedicine could be taken by cancer cell specifically owing to its high CD44 expression on the surface of cancer cell. Subsequently, photodynamic therapy (PDT) generated by pyropheophorbide could contribute the tumor microenvironment immunogenicity and cytotoxic T lymphocytes infiltration. At same time, the level of c-MYC as well as PD-L1 was decreased in the presence of JQ1, thus inhibiting glycolysis and immune evasion. Therefore, a new approach to enhance photoimmunotherapy has been presented to treat pancreatic cancer via provoking T cells activation as well as overcoming adaptive immune resistance. Similarly, Wu *et al.* also have employed HA to enhance selective delivery of nanodrug to tumor sites, thereby achieving a precise and comprehensive blockade of tumor energy metabolism 80. Once within the melanoma cells, the nanodrug induces a rapid and significant increase intracellular Zn^2+^ concentration, inducing a decrease of NAD^+^ and inactivation of GAPDH, achieving effective glycolysis blockade. Additionally, Zn^2+^-activatable DNAzyme that specifically targets and cleaves the GLUT1 mRNA, further inhibiting the upregulation of glycolytic flux.

Glucose and glutamine are pivotal for mammalian cells, serving as the primary source of energy and the building blocks for cell growth and proliferation 81. In the event of glucose metabolism disruption, cancer cells have the ability to reroute their metabolism to rely on glutamine, ensuring a continued supply of energy and the biomass. This metabolic plasticity underscores the limitation of singular therapeutic approaches that target either glucose or glutamine metabolism. While such inhibitions may show promise *in vitro*, their efficacy *in vivo*, particularly in cancer therapy, often falls short of expectations. Recognizing this challenge, Wang and coworkers employed bioinformatics to identify key regulatory genes in metabolic pathways of pancreatic cancer: which is crucial for glucose uptake, and alanine, serine, cysteine transporter 2 (ASCT2), responsible for glutamine transport 82. They discovered that BAY-876 and V-9302 are potent inhibitors for GLUT1 and ASCT2, respectively. To overcome the limitations of single-target therapies, they innovatively co-loaded BAY-876 and V-9302 into human serum albumin nanoparticles through a straightforward self-assembly method. This dual-inhibition strategy effectively targets both glucose and glutamine transporters in pancreatic cancer cells. By inhibiting GLUT1, the strategy deprives tumor cells of their energy supply and triggers oxidative stress. Concurrently, blocking ASCT2 not only further restricts the energy supply but also diminishes the synthesis of the reducing agent GSH, leading to a significant reduction in the cell's antioxidant capacity. This dual action results in a surge of ROS within the cell. The overaccumulation of ROS sets off a cascade of events, activating key proptosis mediators such as caspase 1 and gasdermin D. This activation ultimately leads to a regulated form of cell death known as proptosis, which could be a powerful approach to eliminating cancer cells while minimizing harm to normal tissues. This strategy holds great potential for improving clinical outcomes in pancreatic cancer and possibly other cancer types with similar metabolic dependencies.

Besides utilizing glutamine metabolism, tumor cells adapt by scavenging alternative nutrients including lactate or albumin to sustain their metabolism, when glucose becomes scarce. Capitalizing on the fact that albumin can be internalized by tumors as a nutrient, albumin-based nanotherapeutics have been developed for cancer therapy. Nevertheless, these nanomedicine face challenges due to suboptimal tumor targeting and intracellular uptake. The innate tumor-homing ability of albumin is insufficient to concentrate the drug effectively at the tumor site, which can result in diminished therapeutic efficacy. To enhance the targeting of tumor, bacteria (Escherichia coli Nissle 1917 (EcN)) is used to delivery albumin based nanodrug containing BAY-876 and paclitaxel 83. Upon reaching the tumor tissues, EcN not only homes in on the area but also consumes glucose, reducing its availability for tumor cells. The reduced glucose levels mediated by EcN facilitated the enhanced uptake of BAY-876 bound human serum albumin nanodrugs by CT26 tumor cells, thereby creating a more favorable condition for the therapeutic intervention to take effect. Once internalized, the nanodrug releases BAY-876, which inhibits the glucose transporter, further restricting glucose uptake by the tumor cells. This reduction in glucose levels, both inside and outside the cell, activates AMP-activated protein kinase (AMPK), a key regulator of cellular energy and nutrient signaling. The activation of AMPK stimulates an increase in macropinocytosis, a form of fluid-phase endocytosis. This process, in turn, enhances the internalization of the nanodrug and the delivery of the chemotherapeutic agent, paclitaxel, amplifying the therapeutic effect on the tumor cells. This innovative strategy harnesses the natural metabolic competition between bacteria and tumor cells to improve drug delivery and therapeutic outcomes. By combining the targeting ability of bacteria with the metabolic disruption caused by BAY-876, this approach offers a novel and potentially powerful method for enhancing the efficacy of cancer therapy.

The high-glucose metabolism within the TME, coupled with the desmoplastic tumor's resistance to efficient drug delivery, and the emergence of chemoresistance of gemcitabine, all contribute to the restricted efficacy of GEM. In GEM-resistant cancer cells, the redirection of glycolytic intermediates towards the nonoxidative PPP boosts pyrimidine synthesis and raises deoxycytidine triphosphate (dCTP) levels. The similarity between dCTP and gemcitabine causes competitive inhibition, reducing the drug's ability to integrate into DNA during replication and thus decreasing therapeutic effectiveness of GEM. Moreover, hypoxia within tumors stimulates the sonic hedgehog (SHH) pathway, enhancing smoothened (SMO) expression and GLI transcription factor activity, which drives fibrosis and hinders blood flow, perpetuating a cycle of desmoplasia and hypoxia that undermines therapeutic effectiveness in pancreatic cancer. In pancreatic cancer, miR-519c which is downregulated in pancreatic cancer cells and has the ability to bind to HIF-1 mRNA, which in turn suppresses the expression of hypoxia-inducible factor 1 α (HIF-1α). By inhibiting HIF-1α, miR-519c can increase the sensitivity of pancreatic cancer cells to treatment, offering a potential strategy to overcome chemoresistance and enhance the effectiveness of therapies like GEM. Xin and his coworkers have developed a redox-responsive nanomedicine through combination of GEM as well as miR-519c to resensitize resistant pancreatic cancer cells to GEM 84. In this study, the nanomedicine was modified with EGFR targeted GE11 peptide to enhance its tumor accumulation. And then, the nanomedicine of about 100 nm, designed for pancreatic cancer treatment, efficiently releases miR-519 and GEM upon degradation by GSH, achieving a GEM payload of 14% (w/w) and ensuring nearly complete (90%) GEM release upon exposure to GSH. Subsequently, the expression of both HIF-1α and ABCG2 was decreased by miR-519c, resulting in the decrease of GLUT1 expression as well as cancer cell metabolism under hypoxia. As the decrease of glucose uptake, the pentose phosphate pathway was suppressed, leading to the decrease of dCTP. As a result, cancer cell demonstrated sensitive to GEM to inhibit its proliferation.

### 6.2 Targeting Glucose Depletion by Glucose Oxidase

Glucose oxidase (GOx) has been widely used in various nano medicine systems. Gox can catalyze the oxidation of β-D-glucose into glucono-δ-lactone which will then spontaneously hydrolyzes into gluconic acid. This enzyme reaction requires flavin adenine dinucleotide (FAD) as the coenzyme to transfer the electron from glucose to oxygen. The molecule oxygen is used as the electron acceptor for this reaction and will be reduced into H_2_O_2_.

The intrinsic reaction that GOx catalyzes brings multiple combinational therapies in cancer treatment. The dissipation of glucose will lead to glucose deprivation in the targeted cell, which will enhance the therapeutic efficiency when combining with other treatments like chemotherapy, metal ion therapy and photodynamic therapy. Besides, the *in situ* generation of H_2_O_2_ will significantly increase the intratumorally oxidative stress which would further lead to apoptosis. Under the facilitation of Fenton reagent, the H_2_O_2_ will be further converted into hydroxyl radicals which induce severe oxidative damage inside the tumor cell. Furthermore, the consumption of the oxygen and the formation of gluconic acid in the tumor microenvironment will enhance the characteristics of the TME, therefore amplifying the hypoxia or pH response of the nano vehicle, improving the targeting efficiency of the nano system. As a consequence, GOx can not only be regarded as an effective anti-tumor drug with the combination of glucose deprivation and oxidation therapy, but also outstanding as the excellent adjuvant for an enhanced responsive drug release in TME. The versatile utility of GOx promises a broad application and an increasing research interest in GOx-based anti-cancer nano drug design.

Although ROS-dependent therapy mediated by GOx has attracted much attention in the treatment of cancer. The ECM in the cancer was a barrier to prevent the penetration of nanoparticles. Therefore, the production of high level of ROS and elimination ECM for enhancing the penetration of nanoparticles was effective approach to treat cancer. Recently, Chen group have reported a novel *in situ* polymerizes MSN biocatalysis nanoreactor to enhance ROS generation capability for the treatment of pancreatic ductal adenocarcinoma (PDAC) via combination of chemodynamic therapy (CDT) and PDT 85. In the beginning, collagen in the ECM was broken down by the released collagenase, facilitating the penetration of nanoreactor as well as oxygen infiltration. Subsequently, ultrasmall gold nanoparticles acted as nanozyme to mimic GOx to convert glucose into H_2_O_2_ which was subsequently harnessed by Cu^2+^ to generate ROS through a Fenton-like reaction. In the meanwhile, the ability of photosensitizer to produce singlet oxygen was enhanced due to the infiltration of oxygen. The HMON-Au-Col@Cu-TA-PVP nanoplatform, in conjunction with LI treatment, induced rapid tumor regression and resulted in long-term, tumor-free survival in approximately 80% of the mice, demonstrating superior efficacy compared to other therapeutic strategies. Thus, enhancing ROS therapy was an effective strategy for the treatment of pancreatic cancer.

**Figure 3:**
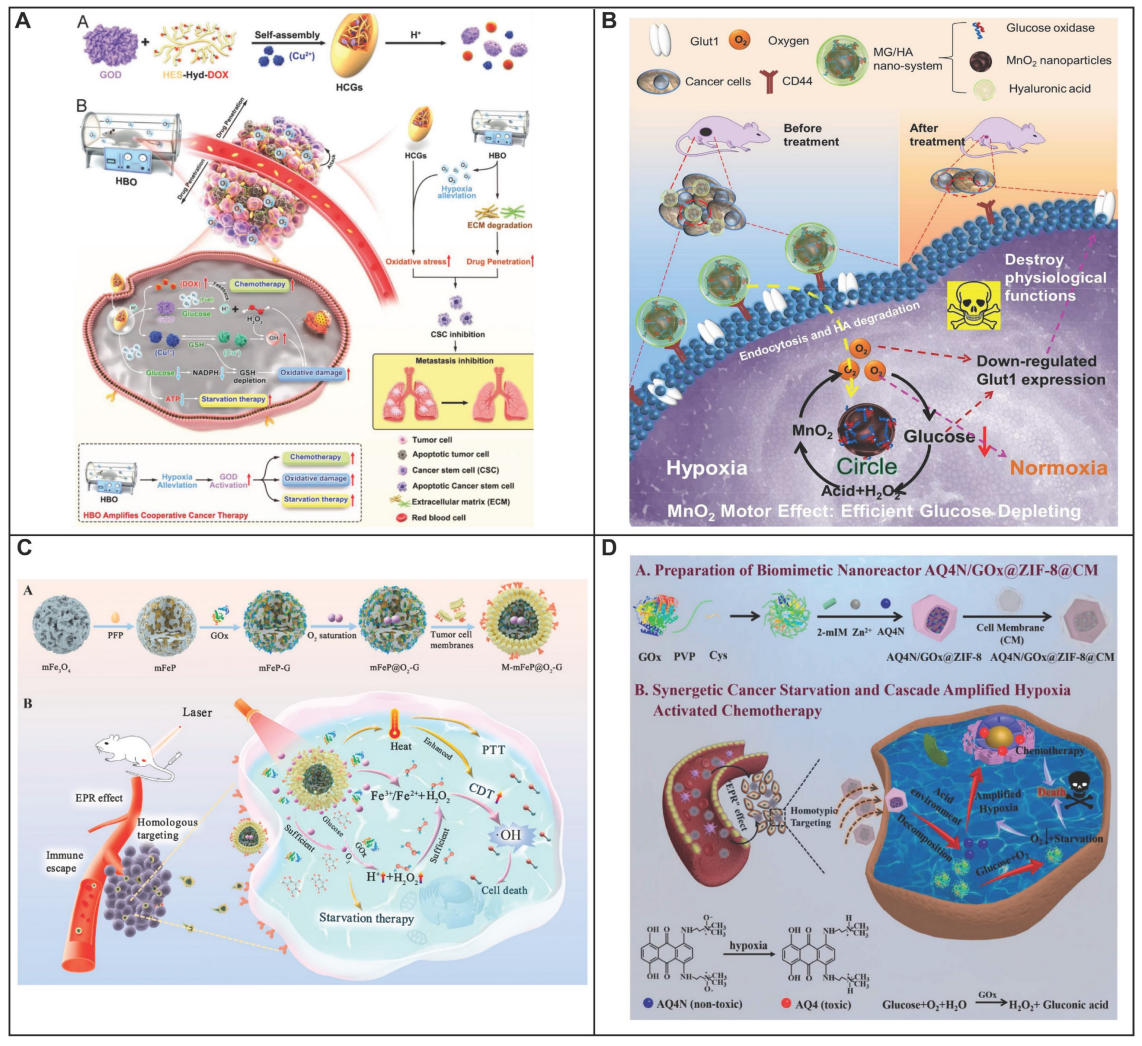
Targeting glucose depletion by glucose oxidase. (A) Schematic diagram showing the preparation of HCG and the therapeutic schematics of HBO-activated HCG-triggered cooperative cancer therapy. Reproduced with permission from ref 90. Copyright 2023 Wiley-VCH. (B) Schematic diagram showing MnO_2_ motor effect in the MG/HA nano-system for starving therapy with interfering Glut1 expression. Reproduced with permission from ref 91. Copyright 2021 American Chemical Society. (C) Schematic diagram showing the tumor microenvironment adaptive nanoplatform for cascaded CDT for the treatment of osteosarcoma. Reproduced with permission from ref 94. Copyright 2023 Elsevier. (D) Schematic diagram showing preparation of biomimetic nanoreactor AQ4N/GOx@ZIF-8@CM. and synergetic cancer starvation therapy and cascade amplificated hypoxia activated chemotherapy. Reproduced with permission from ref 97. Copyright 2021 Elsevier.

During the hydrolysis of glucose by GOx, the enzyme reaction necessitates the presence of FAD as a coenzyme to mediate the electron transfer from glucose to oxygen, generating H_2_O_2_ 86, 87. The intrinsic reaction that GOx catalyzes brings multiple combinational therapies in cancer treatment. The dissipation of glucose will lead to glucose deprivation in the targeted cell, which will enhance the therapeutic efficiency when combining with other treatments like chemotherapy, metal ion therapy and photodynamic therapy. Numerous studies have been focusing on combining chemotherapy drug with GOx, exhibiting an excellent therapeutic efficiency. Zhang and his colleagues have deigned a GOx based, metal-organic framework nanoparticle with tarapazamine (TPZ) as the chemotherapy agent 88. The nanoparticle is coated with erythrocyte membrane to escape from the immune surveillance and have a prolonged blooded circulation. By glucose starvation and hypoxia enhancement form GOx, the cytotoxicity of TPZ is improved, showing a highly effective synergistic tumor therapy with 97.6% tumor growth inhibition. Glucose starvation can also be coupled with other advanced therapy methods like PDT. Li and his colleagues have designed a nano-bioreactor of mem@CAT@GOx@PCN-224. PCN-224 was used as a photosensitizer to generate highly toxic ^1^O_2_, with the combination of glucose starvation by GOx 89. To have a better therapeutic therapy, Li took the tumor hypoxia environment into consideration. Catalase (CAT) was co-delivered with the nanosystem, converting the endogenous H_2_O_2_ back into O_2_, which would further accelerate the consumption of glucose by GOx. Synchronically, the regenerated oxygen can also promote the production of ^1^O_2_, leading into an enhanced cytotoxicity of the photosensitizer. Overall, by constructing a closed catalytic cycle of GOx and CAT, the therapeutic efficiency of PDT would be no longer limited by the stress protein, nor the hypoxia environment in tumor, leading to a very strong inhibition of tumor growth. Addressing the issue of hypoxia, which is common in solid tumors, hyperbaric oxygen (HBO) therapy has been incorporated to enhance oxygen delivery to these regions (Figure 3A) 90. Beyond simply providing oxygen, HBO's impact extends to the degradation of the dense tumor ECM, which is known to impede the distribution of therapeutic agents. This degradation facilitates better accumulation and deeper penetration of nanomedicines within the tumor mass. This formulation has shown an impressive therapeutic profile, effectively targeting not just the bulk tumor cells but also the notoriously resistant cancer stem cells. Manganese dioxide (MnO_2_) can be reduced into Mn^2+^ by H_2_O_2_ to produce O_2_, a process that not only generates oxygen but also enhances the delivery of oxygen, thereby improving the effectiveness of glucose deprivation therapy mediated by GOx. A novel nanoplatform was engineered to augment the therapeutic efficacy of starvation therapy for solid tumors. (Figure 3B) 91. The nanoplatform integrates three key components: GOx, MnO_2_ as well as hyaluronic acid. GOx is responsible for depleting glucose to produce gluconic acid and H_2_O_2_, a reaction that also consumes oxygen. The nanosystem showed specific targeting toward cancer cells with high CD44 expression in CT-26 tumor-bearing mice owing to the presence of hyaluronic acid. Simultaneously, MnO_2_ is introduced to counteract hypoxia and reduce the level of GLUT1, which in turn decreases glucose uptake. Consequently, this approach not only suppresses tumor aggressiveness but also inhibits metastasis. Therefore, the MG/HA nanoplatform presents a promising strategy for cancer treatment, leveraging MnO_2_ to overcome the limitations associated with starvation therapy.

Besides glucose deprivation, the *in situ* generation of H_2_O_2_ will also increase the intratumorally oxidative stress, as another account for GOx cytotoxicity. Hence, a polymeric nanoreactor with GOx-quinone methide (QM) has been constructed to prove this concept 92. Through pH-response, the nanoreactor will dissociate and release the GOx for glucose deprivation and oxidative stress. The codelivered QM will also deplete the intracellular GSH to impair the anti-oxidation agent. Therefore, by synergistically inducing the intracellular oxidative stress in tumor, a great therapeutic efficiency with complete ablation toward A549 tumors was shown by this GOx-QM nanoreactor with a negligible system toxicity. Moreover, when GOx is combined with Fenton reagent, the generated H_2_O_2_ will be converted into the more toxic hydroxyl radicals with a more severe oxidative damage inside the tumor cell. For instance, Huo and his collaborators constructed a mesoporous silica nanoparticle with GOx and Fenton reagent (ultrasmall Fe_3_O_4_ nanoparticles) loaded 93. GOx catalyzes glucose into H_2_O_2_, which will elevate the endogenous H_2_O_2_ level in tumor. Through the catalysis of Fe_3_O_4_ nanoparticles, the elevated H_2_O_2_ will further be disproportionate into highly toxic hydroxyl radical which will lead to cell apoptosis. This proof-of-concept nanomedicine achieved a mild therapeutic effect which requires future optimization and modification.

The efficacy of CDT is often hindered by the TME, characterized by low H_2_O_2_ levels, acidic pH, and hypoxia. Direct administration of GOx into tumor cells can also result in unwanted leakage and clearance by the reticuloendothelial system, posing risks to healthy cells. Moreover, the activity of GOx is dependent on local oxygen availability and specific temperature conditions. Current CDT nanoparticles frequently lack the precision to target tumors effectively, leading to diminished therapeutic impact and increased toxicity. To counter these challenges, a TME-adaptive nanoplatform was developed by Li group. (Figure 3C) 94. This platform is capable of not only reducing the pH and elevating oxygen levels within tumor cells but also efficiently producing high concentrations of H_2_O_2_ *in situ*. It also possesses the ability to specifically target tumors, thereby minimizing adverse effects. Mesoporous Fe_3_O_4_ (mFe_3_O_4_) nanoparticles have been chosen for their expansive surface area and potent photothermal effect, making them ideal as nanocarriers and catalysts for Fenton reactions. These nanoparticles encapsulate GOx to deplete glucose in the TME, generating gluconic acid and H_2_O_2_, which further catalyze the Fenton reaction and produce hydroxyl radicals (⋅OH). To ensure a continuous supply of oxygen for this cascade reaction, perfluorocarbon (PFP), known for its substantial oxygen storage capacity, is integrated into the system. The nanoparticles are cloaked with K7M2-WT (K7M2) osteosarcoma cell membranes to enhance their affinity for homologous tumor cells, thanks to specific surface proteins. Once injected intravenously into mice, these nanoparticles (M-mFeP@O_2_-G) are guided to the tumor sites by the tumor cell membranes and disintegrate in the TME's slightly acidic environment, releasing Fe ions, GOx, and O_2_. This release triggers a self-sustaining catalytic cascade under 808 nm laser irradiation, synergistically combining enhanced CDT, starvation therapy, and PTT to effectively target and destroy tumor cells.

Starvation therapies utilizing GOx face challenges due to the absence of precise targeting and the inhibitory effects of the CD24/Siglec-10 signaling pathway, which can protect cancer cells from being engulfed by macrophages. This pathway hampers the activation of immune cells, thereby shielding tumor cells from the body's natural defenses. To overcome these obstacles, a pioneering approach has been taken by Wang and colleagues, who have combined GOx with Lysosome-targeting chimeras (LYTACs) 95. This fusion not only remedies the targeting deficiency but also potentiates the therapeutic efficacy of GOx in cancer starvation therapy. LYTACs are ingenious small molecules with dual affinity, designed by fusing a protein of interest (POI)-binding element to a lysosome-targeting receptor ligand, such as the cation-independent mannose 6-phosphate receptor (CI-M6PR) and the asialoglycoprotein receptor (ASGPR). The ASGPR, specifically expressed on the surface of hepatocytes, is a reliable target for protein degradation research and liver cancer treatment. The innovative nanoplatform, Nanosphere-AntiCD24, is crafted from polypeptide-modified N-acetylgalactosamine, GOx, and a CD24 antibody. The polypeptide-modified N-acetylgalactosamine self-assembles into nanospheres that can specifically bind to the ASGPR, while the nanospheres' internal hydrophobic structure provides a secure loading space for GOx. The CD24 antibody is crosslinked to the nanospheres, forming novel LYTACs endowed with the capability to degrade CD24, a protein widely expressed in various solid tumors. Once internalized by hepatocellular carcinoma (HCC) cells, the nanosphere-AntiCD24 selectively targets HCC cells that overexpress CD24, facilitating the transport of the CD24 protein to the lysosome for degradation. This strategic degradation weakens the immunosuppressive influence of the CD24/Siglec-10 signaling pathway, thereby reducing the immunosuppressive activity of macrophages. With GOx encapsulated within, the nanosphere-AntiCD24, upon internalization, releases its payload, continuously depleting the endogenous glucose within HCC cells and triggering a starvation response. This breakthrough in nanoplatform design, which integrates targeted delivery of GOx with the degradation of immunosuppressive proteins, presents a promising strategy for enhancing the potency of cancer starvation therapies. It addresses the limitations imposed by the CD24/Siglec-10 pathway and offers a new avenue for more effective cancer treatments.

GOx also aided to expose tumor-associated antigens for an enhanced antitumor response. However, the immunostimulatory potential of GOx is often dampened by immune resistance mechanisms within the tumor microenvironment. One of the most promising directions in tumor immunotherapy involves the combination of starvation/oxidation therapy with the blockade of negative regulatory pathways, such as the immune checkpoint protein indoleamine 2,3-dioxygenase (IDO). IDO, highly expressed in tumors, suppresses T cell proliferation and fosters the expansion of immunosuppressive T regulatory (Treg) cells by converting tryptophan (Trp) to kynurenine (Kyn), making it an ideal target for immune-therapeutic intervention. he synergistic approach of using 1-MT to inhibit IDO, alongside GOx-induced starvation/oxidation therapy, presents a viable strategy for combating tumors with robust immune responses and diminished immune resistance. However, the clinical application of GOx is hindered by its poor bioavailability and susceptibility to rapid inactivation, necessitating the development of multifunctional nanosystems to improve GOx and 1-MT delivery to tumor sites. Dai and colleagues have developed a pH/ROS dual-sensitive, degradable metal-organic framework (MOF) capable of co-delivering GOx and the IDO inhibitor 1-methyltryptophan (1-MT) for integrated tumor starvation/oxidation immunotherapy 96. This nanoreactor is designed to disassemble rapidly in response to the high levels of intracellular ROS found in tumor cells, thereby reducing the long-term toxicity associated with conventional MOFs. The nanosystem, PCP-Mn-DTA@GOx@1-MT, employs a size/charge changeable strategy that sequentially overcomes biological barriers, enhancing delivery efficiency. Upon reaching the weakly acidic tumor microenvironment, the shielding shell of the nanosystem rapidly sheds its PEG component, revealing a polyethylenimine (PEI)-conjugated cationic core. This transformation results in a significantly improved tumor penetration depth and endocytosis. The glucose consumption by GOx leads to an increased production of H_2_O_2_, which is then converted into highly toxic hydroxyl radicals (·OH) through a Mn^2+^-mediated Fenton-like reaction. This process results in the complete degradation of the MOF, the release of the therapeutic agents, and an overall improvement in therapeutic efficacy. By leveraging the enhanced immune response induced by GOx-mediated starvation/oxidation therapy and the suppression of immune resistance through IDO blockade by 1-MT, the PCP-Mn-DTA@GOx@1-MT nanosystem demonstrates a remarkable therapeutic impact. The successful design of this multifunctional nanoreactor sets a precedent for effectively surmounting delivery challenges and achieving superior tumor-killing efficacy through a combination of starvation therapy and immune modulation. Beyond its direct cytotoxic effects, also serve as a potent enhancer for the nanodrug targeting. The enzymatic action of GOx, intensifies the hypoxic and acidic conditions characteristic of the TME. This alteration enables GOx-loaded nanomedicines to exhibit an enhanced hypoxia or pH-responsive drug release profile, a strategy that has gained popularity and widespread application in the development of pH/hypoxia-responsive nanodrugs. Shao and his colleagues designed a novel biomimetic nanoreactor that harnesses the cytotoxicity of GOx and the hypoxia-activated prodrug banoxantrone (AQ4N), encapsulated within a pH-responsive ZIF-8 metal-organic framework (Figure 3D) 97. As GOx continuously consume O_2_ and generate gluconic acid in its enzymatic reaction, the hypoxia activation of AQ4N and the pH-induced disintegration of ZIF-8 are synergistically amplified with the delivery of more nanoreactors to the tumor site. This cascade effect leads to improved therapeutic efficiency and a more precise targeting of the chemotherapeutic agent AQ4N, leading into a better therapeutic efficiency and targeting effect of the chemotherapy drug AQ4N. In summary, GOx is not only an effective anti-tumor agent when combined with glucose deprivation and oxidative therapy but also serves as an exceptional adjuvant for the enhanced, responsive drug release in the TME. The induced hypoxia can further activate secondary agents, creating a multifaceted approach to cancer treatment that is both innovative and efficacious.

The convergence of GOx with prodrugs in synergistic anticancer strategies heralds a transformative approach, promising to revolutionize cancer treatment with unparalleled precision and potency. Traditional combinations of GOx and prodrugs have fallen short due to their non-selective cytotoxicity, affecting both cancer as well as normal cells. By functionalizing GOx or prodrugs with targeting groups, a significant leap towards selective cytotoxicity. To improve therapeutic effect, a dual-targeting approach was developed with combining the precision of folate receptor-targeted nanoreactors with the potency of cyclooxygenase-2 (COX-2)-targeted prodrugs with more selective and effective anticancer strategy 98. The approach combines the precision of folate receptor-targeted nanoreactors with the potency of COX-2-targeted prodrugs. A silica nanoreactor, modified with folate to target cancer cells and encapsulating GOx, is crafted to generate H_2_O_2_. This nanoreactor encapsulates GOX, which upon glucose metabolism, generates H_2_O_2_. The H_2_O_2_ produced not only induces oxidative stress but also serves as a trigger for the activation of a specially designed prodrug. This prodrug is a dual-functional molecule, featuring a COX-2 targeting moiety, Celecoxib, and an anticancer agent, SN-38, connected by an H_2_O_2_-cleavable thioketal linker. The presence of this linker ensures that the prodrug remains inactive until it encounters the elevated H_2_O_2_ levels within the cancer cells. The folate-modified and H_2_O_2_-generating nanoreactor (F-GOX@NR) is rapidly and selectively transported into the folate receptor-positive cancer cells and generates H_2_O_2_ inside the cells by consuming only glucose. The generated H_2_O_2_ induces not only induces cytotoxicity through oxidative stress but also activates the prodrug by cleaving the thioketal linker, releasing the active SN-38. Meanwhile, the Celecoxib component of the prodrug binds to intracellular COX-2, potentially enhancing its accumulation in the cytoplasm and preventing rapid nuclear transport, which could lead to synergistic cell death. The activation process is dramatically accelerated by the elevated H_2_O_2_ levels generated by the F-GOX@NR nanoreactor, far surpassing the rates achievable with the normal intracellular H_2_O_2_ concentrations. By employing this dual-targeting strategy, which combines the specificity of folate receptors and COX-2, the limitations of traditional GOx therapies are effectively addressed. This approach offers a more personalized and effective treatment with reduced side effects, particularly for aggressive forms of cancer, bringing us one step closer to a new era of cancer therapy.

Though naturally occurring GOx is known for its high catalytic activity, it is not without its drawbacks, such as the challenges associated with purification and susceptibility to inactivation. In contrast, the emergence of nanozymes that emulate the catalytic properties of GOx, presents a compelling alternative. These nanozymes feature tunable catalytic efficiency, enhanced stability, and the potential for large-scale production, which positions them as promising candidates for a wide range of applications. There have been several comprehensive reviews on nanozymes with GOx-like activities 99, and therefore, this topic will not be elaborated upon further in this discussion.

### 6.3 Modulation of Glycolytic Enzymes

Nanoparticles can be engineered to deliver small molecule inhibitors or siRNA directly to the intracellular environment, targeting key enzymes involved in glycolysis, such as HK, LDH, and PFK. By inhibiting these enzymes, nanomedicine can slow down or block the glycolytic pathway, leading to a decrease in ATP production and an accumulation of metabolic intermediates that can be toxic to cancer cells.

#### 6.3.1 Targeting Hexokinases

Hexokinases (HKs), which is the initial step to catalyze glucose, is the rate-limiting enzyme in glucose metabolism. Following glucose entry into cell via glut1, glucose was catalyzed to glucose-6-phosphate in the presence of HK, which is the major precursor for glycolysis, hexosamine, PPP as well as glycogenesis. Four HK isoforms (I, II, III and IV) are expressed in human cells, which was encoded by genome. HK I as well as HK II (HK II) are distributed in the outer mitochondria membrane, HK III is located at a perinuclear compartment, and HK IV is disposed in the cytosol. Compared with normal tissues, not only HK I but also HK II are frequently overexpressed in a series of tumors. HK II has a significant role in the cancer cell with the glycolytic behavior. The elevated expression of HK II was related to worse survival. Therefore, HK II is a therapeutic target for fighting cancer. Several inhibitors targeting HK II including jasmonate (MeJA), lonidamine, 2DG, or 3bromopyruvate (3BP) was used to prevent glycolysis. Furthermore, HK II inhibitors combined with other anticancer agents was a promising strategy to cure tumor.

**Figure 4:**
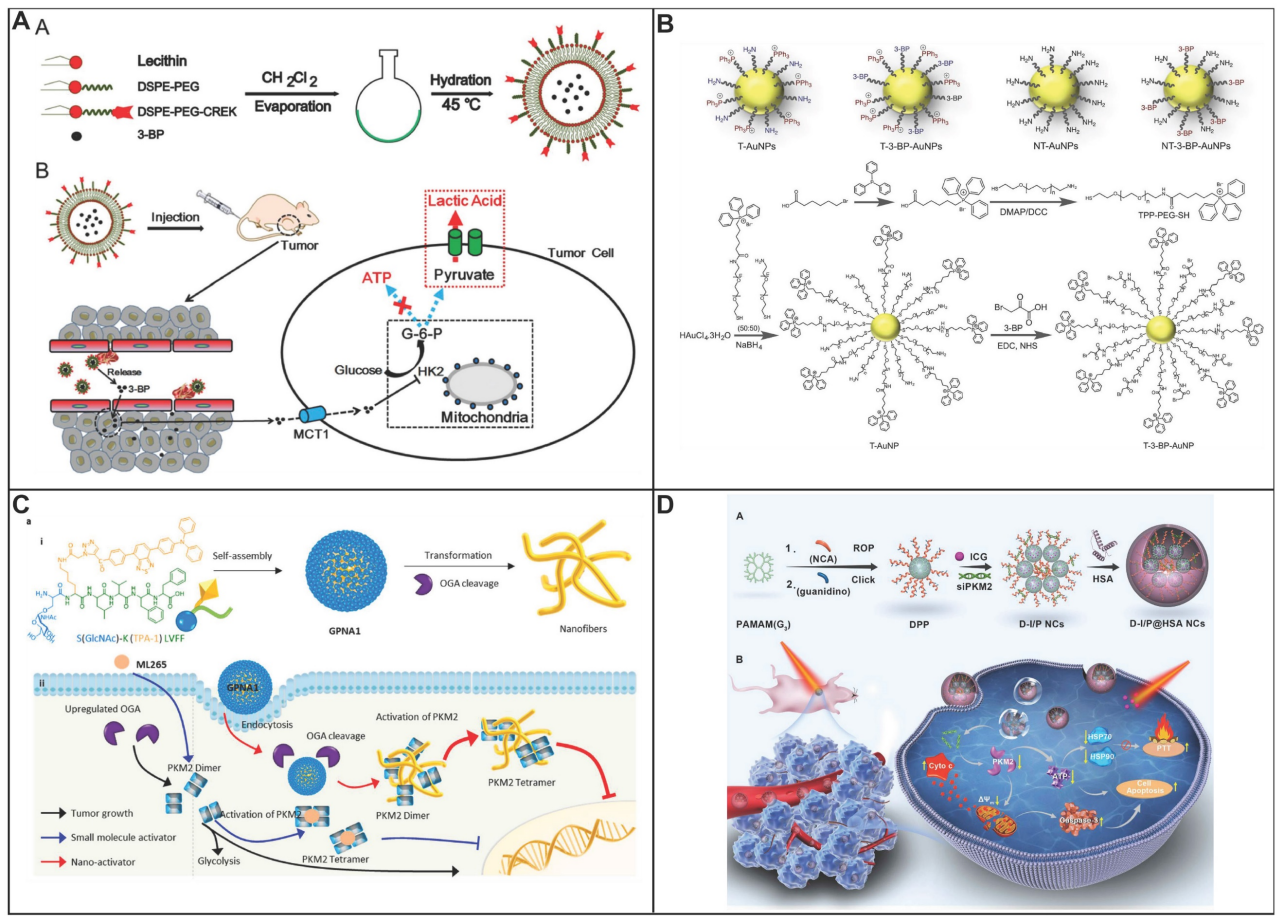
(A) Construction of T-Lipo-3BP nanoparticles and the proposed mechanism of action in tumor vessels through blocking the ATP supply for the tumor cells without affecting the normal cells. Reproduced with permission from ref 200. Copyright 2021 American Chemical Society. (B) Schematic diagram showing engineered AuNP and control NPs used in the mitochondria-targeted delivery of 3-BP. Reproduced with permission from ref 101. Copyright 2018 Royal Society of Chemistry. (C) Schematic diagram showing self-assembly, accumulation and *in situ* fibrillar transformation of GPNA1 in tumor cells, followed by intracellular anti-proliferative events. Reproduced with permission from ref 104. Copyright 2022 Elsevier. (D) Schematic diagram showing spherical helical polypeptide-mediated PKM2 silencing and tumor glycolysis inhibition toward synergistic anti-cancer therapy via sensitization of photothermal ablation and tumor cell starvation. Reproduced with permission from ref 107. Copyright 2019 Elsevier.

In comparison with chemotherapy and radiotherapy, blocking ATP production through the inhibition of aerobic glycolysis that caused cancer cell apoptosis is considered as an attractive strategy to treat cancer. 3-bromopyruvate, pyruvate mimetic, has been demonstrated that could cause apoptosis of cancer cell with highly effective in preclinical studies. Nevertheless, there are several side effects of the inhibitors targeting aerobic glycolysis including unselective delivery, nonspecific targeting as well as low bioavailability, limit its clinical application. In order to address above obstacle, Nie group have developed a nanosystem to delivery 3-bromopyruvate into the tumor tissue, HK II inhibitor, for the treatment of cancer (Figure 4A) 100. The nanosystem consists of three components, liposome that acts as carrier to loading the inhibitor; 3-bromopyruvate which serves as specific inhibitor of HK II to inhibit glycolysis, and CREKA is employed as recognition components for target the tumor vascular endothelium. The proposed nanosystem was capable of targeting tumor vessels, subsequently, 3-bromopyruvate was released to inhibit the generation of ATP, induced cancer cell apoptosis with high selectivity. Thus, the therapeutic strategy could significantly side effect of free 3-bromopyruvate and make it more practical in clinical application.

On account of its non-specificity toward HK1 as well as other metabolic proteins, 3-BP induced systemic toxicity and was unable in clinic to cure disease. Most of HK II is capable of binding to the outer membrane of mitochondria via porin in cancer cells. Therefore, mitochondria-targeted nanoplatform was considered as an effective strategy to increase therapeutic efficacy and reduce side effect. Various nanoparticle could precisely delivery small molecule drugs and improve its pharmacokinetics as well as biodistribution. Gold nanoparticles were widely utilized in medical applications to loading various drugs including small drugs, RNA and DNA owing to controlled geometrical (size and shapes), optical properties and ease of functionalization. Marrache and coworkers have designed a mitochondria-targeted nanoplatform to delivery 3-BP into mitochondria to reduce the production lactate as well as ATP (Figure 4B) 101. Au NPs was decorated with PEG to improve stability *in vivo*. Where 3-BP was conjugated to PEG acted as drug unit. Lipophilic triphenyl phosphonium cations was covalently attached to PEG which served as target unit to decrease the toxicity of drugs. In addition, Au nanoparticles which possess the photothermal capacity was employed to enhance curative effect. Finally, the proposed nanomedicine could selectively cancer cells through the combination of the inhibition of HK II and photothermal treatment.

#### 6.3.2 Targeting Pyruvate Kinase M2

Pyruvate kinase, acting as a rate-limiting enzyme in metabolism, catalyzes the culminating step of glycolysis 102. This pivotal reaction involves the transfer of a high-energy phosphate group from phosphoenolpyruvate (PEP) to ADP, resulting in the formation of pyruvate and the concurrent generation of ATP. PK includes four types of isomers including PKM1, PKM2, PKL, and PKR. Among them, PKM2 is particularly prominent during embryonic development and is notably reactivated in contexts of tissue regeneration and tumorigenesis, underscoring its vital role in supporting the metabolic needs of rapidly proliferating cells. PKM2 plays a dual role in cancer cell survival. Confronted with elevated level of ROS, PKM2 diverts pyruvate towards PPP, thereby generating NADPH to mitigate oxidative stress and prevent cell death. This metabolic flexibility of PKM2 highlights its potential as a strategic target for therapeutic intervention in cancer, offering a unique opportunity to modulate the proliferative and survival mechanisms of cancer cells.

In an innovative approach to cancer treatment, redox modulators are being explored for their ability to adjust the activity of PKM2 by manipulating ROS levels within cancer cells. Duraipandy *et al.* presents a novel application of juglone-modified silver nanoparticles (JFSN) that selectively target cancer cells 103. The JFSN's effectiveness lies in its multi-targeted approach. It inhibits NADPH oxidase 4 (NOX4) and HIF1α, two key players in cancer cell survival, which in turn suppresses PKM2 and disrupts the Wnt/β-catenin signaling pathway-a critical process in cell proliferation. What sets JFSN apart is its dual advantage: it minimizes toxicity to healthy cells while enhancing its impact on cancer cells, with an effective concentration as low as 2.5 μM. This selectivity is achieved by inducing a controlled ROS response that inhibits the enzymes and pathways vital to cancer cell metabolism. By strategically disrupting the glycolytic pathway, JFSN initiates apoptosis in cancer cells, offering a promising strategy in the fight against cancer. This targeted therapy represents a significant step forward in the development of more effective and less toxic cancer treatments.

Serine, a natural substrate, can bind to N-terminal domain of PKM2 to promote its tetramerization, but its effect is limited. To amplify this activation, small molecules have been identified to act as PKM2 activators. Hou *et al.* have developed a glycopeptide-based nano-activator that, upon exposure of the serine binding site by the tumor-overexpressed enzyme O-GlcNAcase (OGA), transforms from a spherical to a fibrous shape. (Figure 4C) 104. This change not only increases the serine binding sites but also boosts the accumulation and retention of PKM2 at the tumor site, thereby promoting its tetramerization. Importantly, this localized nano-activator sustainably reprograms tumor metabolism, leading to a significant anti-tumor effect. Even in the presence of chemotherapy-resistant, highly metastatic tumors, the glycopeptide-based nanotherapeutics have shown improved therapeutic efficiency by inducing a metabolic slowdown that makes the tumor more sensitive to chemotherapeutic agents, resulting in an initial suppression of tumor growth and an extended survival rate. This innovative strategy leverages the power of PKM2 activation and nanoformulations to offer a promising new avenue in the fight against cancer.

In glioma, the overexpression of ALDH1L1, a key enzyme in the 10-formyl-tetrahydrofolate-NADH-ATP metabolic axis, parallels that of PKM2. To effectively disrupt tumor energy metabolism by impeding ATP production, a synergistic therapeutic approach is warranted-one that concurrently targets glycolysis and NADH-ATP metabolism. Zhao and colleagues have delineated an ATP-deprivation strategy that inhibits PKM2-mediated glycolysis and ALDH1L1-mediated NADH/ATP metabolism 105. This strategy employs the co-encapsulation of disulfiram (DSF), an ALDH1L1 inhibitor, and shikonin (SHK), a PKM2 inhibitor, to comprehensively suppress the tumor's energy supply. Addressing the blood-brain barrier's (BBB) limitations on therapeutic efficacy, albumin and lactoferrin were selected as targeting ligands to enhance the penetration of these inhibitors into the tumor microenvironment. The synergistic potential of the DSF and SHK combination was evaluated using the combination index (CI), with a CI value below 1 signifying a synergistic interaction. The optimal molar ratio of DSF to SHK, established at 2:1, yielded a CI of 0.486, underscoring the synergistic effect of this drug pairing. This combination reduced the IC_50_ to 0.2 μM, a substantial decrease from the individual IC_50_ values of 3 μM for DSF and 0.8 μM for SHK. The nanoformulation of these agents further enhanced efficacy, achieving an IC_50_ of 0.15 μM. Beyond inhibiting glycolysis and reducing lactate and ATP production, this therapy also diminished NADH generation, effectively shutting down alternative energy supply routes. Moreover, SHK-induced inhibition of glycolysis triggered immunogenic cell death (ICD), alleviating immunosuppression. The downregulation of FROUNT expression, a factor that promotes TAMs and tumor progression, facilitated the polarization of TAMs to the M1 phenotype. These multifaceted effects culminated in a robust inhibition of glioma growth, with a 90% reduction rate and a median survival time extended by approximately twofold relative to the saline group. An innovative glioma-targeting approach was introduced, offering a robust strategy for combination therapy at a fixed dosage, designed to effectively deplete ATP and enhance the therapeutic impact on glioma.

Elevated levels of PKM2 in tumor cells are a driving force behind their reliance on glycolytic metabolism for energy production, which in turn powers ATP-binding cassette (ABC) transporters and contributes to chemoresistance observed across various cancers. To counteract this drug resistance, the glycolytic metabolism was disrupted, resulting in not only depriving tumor cells of energy but also triggering the mitochondrial apoptosis pathway, thereby enhancing the efficacy of chemotherapy. A sophisticated nanocomplex was crafted, encapsulating doxorubicin (DOX) within the hydrophobic core of guanidine-rich, spherical helical polypeptides (DPPs) and complexing with PKM2 siRNA to form a polyelectrolyte nanostructure 106. This novel delivery system exploited the DPP's multivalent topology for efficient trans-membrane transport of PKM2 siRNA into DOX-resistant cancer cells, downregulating PKM2 to disrupt glycolysis, thereby starving ABC transporter-mediated drug efflux and reversing chemoresistance. The concomitant induction of apoptosis through mitochondrial dysfunction and ROS generation synergized with chemotherapy, amplifying the anti-cancer effect. The nanocomplex's surface was decorated with HA to enhance tumor targeting via CD44 overexpression and to mitigate toxicity, extending circulation time significantly. To enhance tumor targeting, HA was integrated onto the nanocomplex surface, leveraging its affinity for the overexpressed CD44 on cancer cell membranes. HA also neutralized surface charges, reducing toxicity, and improving serum stability and circulation time. The nanocomplexes, with HA modification, demonstrated a half-life of 4.21 h in blood circulation, significantly longer than free DOX (0.32 h) and non-HA nanocomplexes (1.28 h). Demonstrating pH-responsive drug release, the nanocomplexes showed superior blood circulation longevity and facilitated escape of DOX from endolysosomal entrapment, culminating in a markedly enhanced survival rate *in vivo*. This integrative approach, merging RNAi-mediated metabolic intervention with targeted chemotherapy, sets a precedent for the potent and precise targeting of drug-resistant tumors. he nanocomplexes also exhibited pH-responsive drug release, with increased DOX release at acidic pH values due to protonation-enhanced solubility. Their spherical helical polypeptide component endowed the nanocomplexes with potent membrane activity, facilitating escape from endolysosomal entrapment post internalization. *In vivo*, these nanocomplexes significantly increased drug accumulation in drug-resistant cancer cells and promoted nuclear DOX delivery.

Dang *et al.* devised a strategic approach to cancer therapy by concurrently targeting glycolysis and PTT (Figure 4D) 107. By silencing PKM2, they curtailed the glycolytic metabolism in tumor cells, depriving them of the energy necessary for HSP synthesis and thereby increasing the susceptibility of tumor cells to photothermal ablation induced by indocyanine green (ICG). This energy depletion, orchestrated by siPKM2, not only starved the tumor cells but also intensified the synergistic anticancer impact when combined with PTT. To minimize cytotoxicity, the researchers utilized multivalent helical polypeptides with high siRNA binding affinity and reduced cytotoxicity. The nanocomplexes (NCs) were then coated with human serum albumin (HSA), a strategy that masked the positive surface charges, thereby enhancing serum stability and extending blood circulation times. This coating also facilitated passive targeting of the tumor through the enhanced EPR effect. The siPKM2-induced energy deprivation, in conjunction with PTT, synergistically bolstered the therapeutic efficacy, offering a dual-pronged strategy to combat cancer.

Mitochondria are central to the energy metabolism of cancer cells, balancing the interplay between OXPHOS and glycolysis. Given the sensitivity of tumor cells to mitochondrial dysfunction, targeting these organelles has emerged as a promising anticancer strategy. Yang *et al.* introduced a mitochondria-targeting nanomedicine designed to intervene in the energy metabolism of cancer cells by modulating both PKM2 and FASN 108. The nanoplatform, CUR@DNA-FeS_2_-DA, demonstrated superior photothermal properties and an inherent ability to penetrate mitochondria. Upon exposure to near-infrared (NIR) irradiation, it facilitated the release of curcumin, yielding a markedly enhanced inhibitory effect on tumor cells. This multimodal therapeutic approach outperformed conventional photothermal therapy or curcumin treatment alone, showcasing the potential of mitochondrial targeting in cancer treatment.

### 6.4 Targeting Lactate Metabolism Regulation

Lactate, 2-hydroxypropanoic acid, is a hydroxycarboxylic acid that exists in the human body. During aerobic glycolysis, lactate can be generated or eliminated through a reversible oxidoreduction reaction that is catalyzed by the enzyme LDH. In this reaction, pyruvate is converted to lactate while NADH is oxidized to NAD^+^. Substantial quantities of lactate and H^+^ ions are generated and released into the extracellular space, ultimately contributing to the composition of the TME. Elevated levels of tumor lactate have been linked to increased metastasis, recurrence of tumors, and a generally unfavorable prognosis. Lactate influences cancer cell metabolism and exerts effects on the surrounding environment that contribute to tumor development. Tumor cells can utilize lactate for energy and distribute it to nearby cancer cells, stromal cells, and endothelial cells, leading to metabolic reprogramming. Furthermore, lactate is involved in promoting inflammation within the tumor, as well as acting as a signaling molecule that fosters angiogenesis. As a key signaling molecule in the progression of cancer, oversecreted lactate in the TME leads to acidification with a pH range of 3.3 to 6.9 109. This acidic environment is conducive to processes such as tumor growth promotion, angiogenesis, metastasis, resistance to therapy, and, crucially, immunosuppression, all of which are associated with negative outcomes. Therefore, lactate is considered a significant oncometabolite that is integral to the metabolic reprogramming occurring in cancer.

Lactate can be generated or eliminated through a reversible oxidoreduction reaction that is catalyzed by the enzyme LDH. In this reaction, pyruvate is converted to lactate while NADH is oxidized to NAD^+^. High levels of the LDHA are observed in both muscle tissue and tumors. The primary sources of lactate in humans are pyruvate and alanine, and it represents the final product of both glycolysis and the pentose phosphate pathway. The conversion of lactate back to pyruvate by LDH in the cytosol marks the initial step in the clearance of lactate. The metabolism of lactate is a dynamic and tissue-specific process.

The transportation of L-lactate is primarily facilitated by monocarboxylate transporters (MCT1, MCT2, and MCT4). MCT4 predominantly mediates the efflux of lactate, while MCT1 and MCT2 are capable of transporting lactate in both directions. Additionally, two sodium-coupled monocarboxylate transporters, SMCT1 and SMCT2 (SLC5A12), are involved in the active uptake of L-lactate into cells. This complex interplay of transport and metabolism underscores the multifaceted role of lactate in cancer biology and its potential as a target for therapeutic intervention.

#### 6.4.1 Targeting Lactate Dehydrogenase Inhibition

LDH is a crucial tetrameric enzyme, playing a vital role in the metabolic pathway. It is composed of two different subunits, LDHA and LDHB, encoded by the LDHA and LDHB genes, respectively. These subunits can combine to form five distinct isoforms of LDH, known as LDH1-LDH5, each with unique metabolic characteristics based on their subunit composition. Notably, LDHA, or LDH-M, is particularly favored for its ability to reduce pyruvate to lactate, a process that also involves the oxidation of NADH to NAD^+^. This enzyme is often the focus of medicinal chemistry due to its highly expressed in various cancers and its critical role in tumor growth, invasion, and maintenance. Inhibiting LDHA, as seen in the case of LDH5, can lead to an increase in mitochondrial respiration, which in turn reduces cellular viability under hypoxic conditions. This inhibition is beneficial as it can contribute to the suppression of tumor growth, invasion, and metastasis. As a result, LDHA has emerged as a significant biomarker and a promising target for the diagnosis and treatment of a wide range of cancers. Furthermore, the inhibition of LDHA also results in a decrease in ATP levels, which can trigger an increase in oxidative stress. This heightened oxidative stress can ultimately lead to cell death, providing an additional layer of therapeutic potential in the fight against cancer. The strategic targeting of LDHA presents a multifaceted approach to cancer treatment, offering both metabolic regulation and a means to induce cell death through oxidative stress.

**Figure 5:**
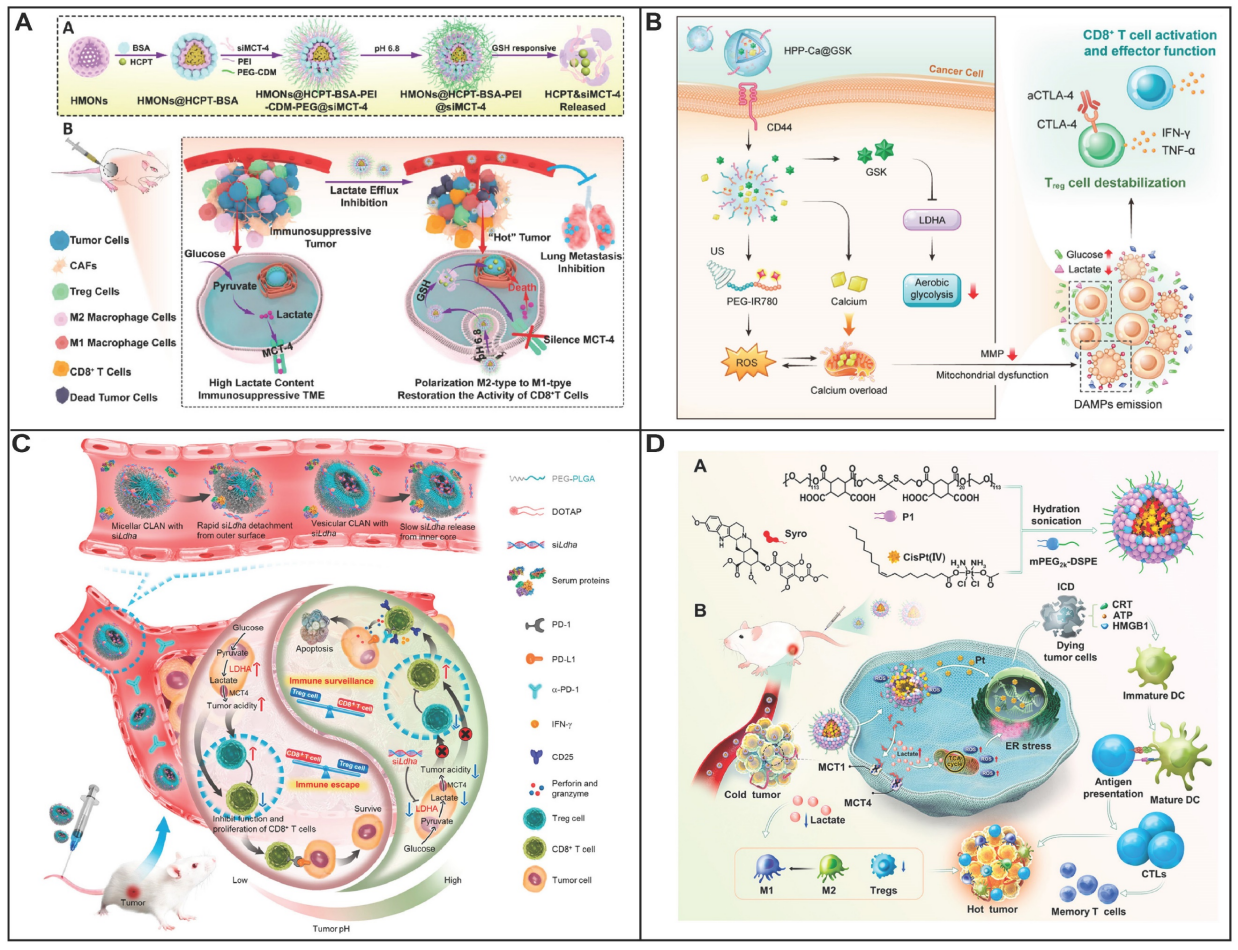
Targeting lactate metabolism regulation. (A) Nanomediated reversion of tumor immunosuppressive microenvironment through tumor acidity modulation. Reproduced with permission from ref 110. Copyright 2019 American Chemical Society. (B) Schematic diagram showing HPP-Ca@GSK nanomedicines for augmenting antitumor immunity via sono-metabolic therapy. Reproduced with permission from ref 111. Copyright 2023 American Chemical Society. (C) Schematic diagram showing the cascaded responsive nanoplatform for enhancing tumor chemo-immunotherapy via inhibiting lactic acid efflux. Reproduced with permission from ref 117. Copyright 2020 American Chemical Society. (D) Schematic diagram showing NP2 mediated inhibition of monocarboxylic acid transporters to enhance chemoimmunotherapy by overcoming low immune response in osteosarcoma. Reproduced with permission from ref 118. Copyright 2024 Elsevier.

Cancer cells possess superior ability to capture glucose which outcompetes the tumor-infiltrating T lymphocytes (TILs), leading to a glucose-starved TME. This scarcity of glucose is coupled with an excessive production of lactate, which acidifies the environment. The acidic TME specifically impairs the efficacy of CD8^+^ TILs, weakening their capacity to fight cancer. On the other hand, it bolsters the suppressive capabilities of rTregs, exacerbating immune suppression. This dual effect not only undermines the immune system's efforts to combat the tumor but also paves the way for the tumor's aggressive proliferation and metastatic spread. Targeting the inhibition of tumor lactate may be instrumental in bolstering the immune response, thereby curbing tumor growth and metastatic spread. Recently, Zhang *et al.* have developed a nanomedicine to delivery LDHA siRNA as well as PD-1 for neutralization of tumor acidity and further immune therapy (Figure 5A) 110. Cationic lipid-assisted nanoparticle serves as carrier to load siRNA which was capable of decreasing the LDHA level in cancer cells. As a result, pyruvate metabolism was reprogrammed, resulting in the decrease of lactate generation in cancer cells with the neutralization of pH in tumor microenvironment. It could be observed that the infiltration of both CD8^+^ T cells and NK cells was increased. In the meanwhile, the density of immunosuppressive T cells was reduced after treatment with LDHA siRNA. Breast cancer and melanoma growth was inhibited after tumor acid neutralization in combination with anti-PD 1 therapy. The blockade of cytotoxic T lymphocyte-associated protein-4 (CTLA-4) is a pivotal strategy which inhibits Treg cell stability by modulating their glucose metabolism, thereby disrupting their suppressive function. LDHA inhibitors, can reshape TME by reducing lactate levels, consequently preserving more metabolic resources for CD8^+^ TILs. However, the success of immunotherapy is not contingent solely on the existing CD8^+^ TILs. Mitochondrial dysfunction leads to an increased emission of DAMPs, which in turn, enhances the presentation of tumor antigens and the recruitment of additional CD8^+^ TILs. Inhibiting aerobic glycolysis induces mitochondrial dysfunction in cancer cells which further reprogram the TME. In Yan's work, a novel HA-modified metalphenolic nanosystem has been introduced, designed to target tumors and amplify antitumor responses (Figure 5B) 111. An LDHA inhibitor (GSK2837808A), IR780, and calcium is integrated which is a multifunctional therapeutic agent that operates on several fronts to enhance the immune response against cancer. The LDHA inhibitor GSK2837808A within the nanosystem restricts glucose consumption in tumor, thereby creating a TME that is more favorable for CD8^+^ TILs. Elevated glucose availability and reduced lactate levels conditions that are detrimental to the stability of Tregs but beneficial for the activation and function of CD8^+^ TILs. Additionally, the nanosystem incorporates a PEGylated IR780 sonosensitizer, which, when activated by US, generates ROS. This sonodynamic effect synergizes with the calcium overload leads to significant mitochondrial dysfunction in cancer cells, resulting in the release of substantial amounts of DAMPs, which are key in activating CD8^+^ T cells and increasing the infiltration of CD8^+^ TILs into the tumor site. Combined with CTLA-4 blockade, represents a coordinated approach to immunotherapy. By modulating the functions of immune cells within the TME, the metal-phenolic nanomedicines are capable of inducing a significantly enhanced antitumor response. The proposed strategy not only targets the metabolic pathways of cancer cells but also leverages own immune system of body to combat the tumor more effectively.

Blocking energy supply by glycolysis inhibitors have the ability to enhance the sensitivity of cancer cells to other therapeutic treatments. FX11, a LDHA inhibitor, may redirect pyruvate towards mitochondrial metabolism, which in turn increases the production of ROS within the cell. Piezodynamic therapy (PzDT), which leverages ROS generated by piezoelectric nanomaterials. This method is particularly effective due to the non-invasive nature of ultrasound, which can penetrate deeply into tissues. PzDT, when activated by US, shows great potential for treating deep-seated tumors. However, due to the diverse and intricate nature of tumors, PzDT alone might not be adequate for complete tumor elimination. A combination therapy involving the LDHA inhibitor FX11, and piezoelectric WS_2_ nanosheets, which can be activated by ultrasound for PzDT, could offer a more effective treatment strategy for cancer 112. The WS_2_ nanosheets, which are two-dimensional, have been modified with PEG to enhance their stability and biocompatibility within the body. Furthermore, to ensure that the therapy targets the mitochondria-the critical cellular organelles involved in cell death and survival-a positively charged, lipophilic agent known as triphenylphosphine (TPP) has been used to decorate the PEGylated WS_2_. This modification allows the WS_2_ nanosheets to specifically target the mitochondria, enhancing the effectiveness of the treatment. The combined effect of these WS_2_ can lead to oxidative damage to the mitochondrial membranes, causing a loss of membrane potential and ultimately triggering programmed cell death, or apoptosis.

Targeted LDHA to prevent M2 of TAM in TME could enhance the efficacy of oxaliplatin (OXA) chemotherapy for colorectal cancer. By employing a cationic polymer, APEG-PAsp(PEI) (PAPEI), to deliver LDHA siRNA, the LDHA gene is effectively silenced, leading to a significant reduction in M2-like TAMs and a marked increase in the M1-like phenotype, which is more antitumor 113. The nanocomplex not only enhances OXA-induced autophagy in tumor cells but also mitigates the lactic acid accumulation in the TME, thereby inhibiting the M2-like polarization of TAMs and amplifying the overall antitumor immune response. By integrating gene silencing with chemotherapy offers a multifaceted therapeutic intervention that targets both the tumor cells and the immune microenvironment, potentially leading to a more effective treatment for colorectal cancer. The overexpression of LDHA is associated with increased secretion of granulocyte colony-stimulating factor (G-CSF) or granulocyte-macrophage colony-stimulating factor (GM-CSF), which in turn boosts the recruitment of myeloid-derived suppressor cells (MDSCs). This process facilitates the secretion of the immunosuppressive cytokine interleukin-10 (IL-10), impairing the functionality of T cells and natural killer (NK) cells. Targeting LDHA through its knockdown in glycolysis presents a potential therapeutic strategy to curb MDSCs recruitment, alleviate immunosuppression, and enhance tumor immunogenicity via the autophagy-related mechanism and DOX-induced ICD effects. Xia and colleagues have engineered a redox-responsive nanoassembly capable of co-delivering LDHA siRNA and DOX to enhance cancer immunochemotherapy 114. The nanoassembly, fortified with the hemolysis safety afforded by the protective shielding of natural PEG on its cationic PAMAM core, ensures a secure and targeted delivery system for therapeutic agents. To ensure precise targeting of tumor cells, the nanoassembly is decorated with RGD peptides, which recognize integrins overexpressed on TNBC cells, thereby enhancing cellular internalization. Once internalized, the nanoassembly escapes endosomes/lysosomes and disintegrates under the intracellular GSH environment through thiol-disulfide exchange, promoting the release of DOX and LDHA siRNA and leading to LDHA silencing. This targeted silencing triggers the AMPK-ULK1 signaling pathway, modulating the synthesis of G-CSF and GM-CSF by diminishing the expression of the autophagy-associated LAP protein, thereby reprogramming the immunosuppressive tumor microenvironment. Concurrently, DOX induces ICD in TNBC cells, promoting the exposure of DAMPs. The RGD decoration endows the nanoassembly with active tumor-targeting capabilities, facilitating efficient delivery of both DOX and LDHA siRNA to the tumor site. Collectively, this redox-responsive nanoassembly has demonstrated enhanced tumor suppression and immune microenvironment remodeling in a TNBC model, opening up new avenues for the development of immunochemotherapy strategies.

Overexpressed LDHA exhibited the immunosuppressive effects in cancer cells, which can activate tumor-associated fibroblasts (TAFs) and impede drug penetration, thereby diminishing the efficacy of DOX-induced ICD, Wu *et al.* have developed a strategy using LDHA as a molecular imprinting template to create a surface-imprinted polymer, LDHA@MIP-DSD 115. LDHA@MIP-DSD is designed to inhibit LDHA activity, effectively deactivating TAFs and enhancing the ability of therapeutic agents to penetrate the tumor stroma, thereby gaining access to cancer cells. Once internalized by cancer cells, LDHA@MIP-DSD responds to the intracellular GSH concentration, releasing DOX and a potent autophagy inducer, SLN. The induction of autophagy by SLN not only triggers tumor cell death but also elicits a robust antitumor immune response. Treatment with LDHA@MIP-DSD has been shown to significantly increase the infiltration of CD8^+^ T cells into the tumor by 11-fold, thereby enhancing the immune attack on cancer cells. Furthermore, LDHA@MIP-DSD demonstrates significant palliative effects on tumor growth and extends survival in a 4T1 tumor model. By leveraging the specific binding of molecularly imprinted LDHA and the autophagy induction by SLN, the LDHA@MIP-DSD treatment offers an optimal combination of immune stimulation and antitumor efficiency, further amplified by its enhanced ICD effect.

#### 6.4.2 Targeting Monocarboxylate Transporters

Lactate, due to its hydrophilic properties, necessitates the use of specialized MCTs to traverse cellular membranes. These MCTs, integral to the solute carrier (SLC) 16A family and comprising 14 unique members, are vital for preserving intracellular acid-base homeostasis and creating a localized acidic niche. Among them, MCT1 and MCT4 have been identified as pivotal for tumorigenesis, with their overexpression in various cancers including breast, colon, pancreatic, glioblastoma, prostate, and renal cell carcinoma-correlating with a poor clinical outcome and a significant role in the acquisition of multidrug resistance. Elevated expression of MCT1 and MCT4, observed across a spectrum of cancers such as breast, colon, pancreatic, glioblastoma, prostate, and renal cell carcinoma, is implicated as a critical factor in the progression of multidrug resistance 116. The upregulation of MCT1 and MCT4 is not only linked to the growth and proliferation of cancer cells but also represents a metabolic vulnerability that can be targeted therapeutically. Inhibition of these transporter proteins can lead to a buildup of intracellular lactate, which has the potential to trigger apoptosis in cancer cells, thereby improving the tumor microenvironment by lowering extracellular lactate levels.

Accumulation of intracellular lactate facilitated by MCTs induces a tumor-acidifying environment and attracts immunosuppressive cells, thereby significantly undermining the effectiveness of immunotherapeutic interventions. Lactate facilitates the reprogramming of TAMs from the pro-inflammatory M1 subtype to the immunosuppressive M2 subtype through lactate-induced epigenetic changes. By reducing the expression of MCT-4 mRNA with siRNA, lactate levels within tumor cells are increased, which in turn enhances tumor cell apoptosis. Concurrently, a reduction in extracellular lactate levels can modulate the TME. This approach of integrating lactate efflux inhibition with chemotherapy may serve as an innovative strategy to curb tumor advancement. A novel strategy was designed to eradicate immunosuppressive TMEs, transforming these tumors into "hot" tumors that elicit robust antitumor responses through the combined actions of lactate efflux inhibition and chemotherapy. A redox (GSH)-responsive, hollow mesoporous silica-based nanocarriers has been engineered to encapsulate the anticancer drug HCPT and to adsorb bovine serum albumin (BSA) onto its surface (Figure 5C) 117. These nanocarriers, which contain siRNA that targets MCT4, are further coated with a layer of positively chargedPEI to boost their uptake by tumor cells. The nanoplatform's design also includes an acid-detachable PEG segment that enhances stability in the bloodstream and allows for targeted charge reversal for improved tumor cell uptake. This multifaceted approach ensures a balance between safety and therapeutic efficacy by controlling activation at various stages: during blood circulation, tumor cell uptake, and intracellular release. The MCT-4 gene silencing action of the nanoplatform leads to elevated intracellular lactate, triggering apoptosis in tumor cells and subsequently lowering extracellular lactate levels. This shift alters the TAM phenotype, rejuvenating CD8^+^ T cell functionality. Moreover, the nanoplatform is capable of stimulating the immune system and preventing lung metastasis. In summary, the nanoplatform loaded with HCPT and MCT-4 siRNA effectively halts tumor growth and mitigates lung metastasis by integrating the effects of chemotherapy and lactate efflux inhibition. This innovative strategy is anticipated to convert immunosuppressive tumors into "hot" tumors, thereby inhibiting tumor progression and lung metastasis.

Intracellular lactate accumulation induces a profound reconfiguration of mitochondrial metabolism and augments OXPHOS pathways via the inhibition of monocarboxylate transporters MCT1 and MCT4. This inhibition precipitates a marked increase in ROS, which synergistically enhances the therapeutic efficacy of cisplatin-based chemotherapy which causes ICD. Despite the potential of this approach, the clinical application of both cisplatin and syrosingopine is impeded by their limited tumor-targeting capabilities and considerable systemic toxicity, thereby restricting their potential in advancing cancer immunotherapy. In an innovative advancement, the Xiao research group has developed a ROS-sensitive polymer (P1) and fabricated nanoparticles (NP2) by encapsulating P1 with a cisplatin (IV) prodrug and syrosingopine.( Figure 5 D) 118. The encapsulation strategy ensures that upon cellular internalization, syrosingopine is released to function as an MCT1/4 inhibitor. This release results in a diminished extracellular lactate concentration and a concomitant rise in intracellular lactate, effectively redefining the lactate distribution gradient. The elevated intracellular lactate, in concert with cisplatin, stimulates ROS generation, leading to endoplasmic reticulum (ER) stress induced by ROS. This ER stress initiates ICD, facilitating the release of DAMPs from the demise of cancer cells. The liberation of DAMPs is instrumental in the maturation of DCs, enhancing their capacity for antigen presentation and instigating a potent adaptive immune response. Furthermore, the reduction of lactate within the TME not only promotes the differentiation of M1-type TAMs but also attenuates the presence of Tregs, thereby mitigating the immunosuppressive milieu of the TME. This novel therapeutic strategy harmonizes chemotherapy and immunotherapy through the targeted delivery of an MCT1/4 inhibitor and a cisplatin (IV) prodrug, thereby sensitizing the antitumor effects of cisplatin and activating a robust antitumor immune response. In addition to the chemotherapeutic effects, the manipulation of lactate metabolism also potentiates ferroptosis within the TME. A TME-activated immunomodulatory nanoadjuvant has been ingeniously designed to deplete GSH and generate ROS through a combination of CDT and PTT, thereby inducing ICD and achieving remarkable outcomes in both ferroptosis and ICD 119. Syro inhibits lactate efflux, resulting in intracellular acidification and extracellular pH neutralization. This dual action not only sensitizes cells to ferroptosis but also alleviates immunosuppression, leading to extensive maturation of dendritic cells in the lymph nodes and the effective infiltration of T lymphocytes. Concurrently, the presence of Tregs is significantly reduced. This multifaceted approach not only suppresses the proliferation of the primary tumor but also significantly impedes the spread of cancer to distant tumors and the lungs, offering a comprehensive therapeutic strategy against cancer progression and metastasis. Similar to this work, Syro was effectively delivered via a manganese-based MOF to potentiate CDT and bolster immunotherapy 120. Consequently, the integration of ROS-generating therapies, such as PDT, SDT, radiotherapy, and CDT, with the strategic inhibition of lactate metabolism, emerges as a promising strategy. This multimodal approach not only suppresses tumor growth by elevating ROS levels but also rejuvenates the immune response. It facilitates the M1 polarization of TAMs, activates dendritic cells, and enhances the activity of cytotoxic T lymphocytes, while concurrently diminishing the presence of immunosuppressive cells, thereby creating a more favorable microenvironment for effective cancer immunotherapy.

Lactate has been innovatively repurposed as a substrate for the production of ROS through the enzymatic activity of lactate oxidase, augmented by the synergistic effects of CDT 121. This biochemical cascade catalyzes the conversion of intracellular lactate into hydrogen peroxide, with the concurrent acidification of the intracellular milieu serving to amplify the self-replenishing nanocatalytic reaction. The ensuing surge in ROS levels inflicts mitochondrial damage, thereby impeding the OXPHOS process as a compensatory energy supply mechanism in the face of impaired glycolysis within tumor cells. Simultaneously, the immunomodulatory landscape is meticulously engineered through the reversal of the pH gradient, which orchestrates the liberation of pro-inflammatory cytokines. This strategic intervention restores the functionality of effector T and NK cells, bolsters the polarization of M1-type TAM, and effectively suppresses the influence of regulatory T cells. Besides LOD, intracellular lactate accumulation can be effectively mitigated by a yolk-shell structured CoP/NiCoP (CNCP) photocatalyst, which functions as a hole scavenger to enhance photocatalytic efficiency 122. Under 808 nm laser excitation, CNCP generates ROS and H_2_, which, in concert, restrict the ATP necessary for HSPs synthesis by inducing severe mitochondrial dysfunction, thereby enabling efficient mild PTT. The yield of ROS and H_2_ also significantly impairs the mitochondrial respiratory chain, disrupting the intracellular energy supply essential for HSPs synthesis and triggering a mild PTT that intensifies ICD. Simultaneously, the sustained heat induced by the NIR laser effectively releases the encapsulated fluoxetine (Flu), an MCT4 inhibitor, which implements a blockade of lactate efflux in 4T1 cells. This action neutralizes the immunosuppressive acidic TME and sequesters lactate within the cells, further enhancing therapeutic effect of ICD, subsequently leading to extensive dendritic cell maturation and T lymphocyte infiltration, which also helps in curbing tumor spread and lung metastasis. CNCP achieves an enhanced immunotherapeutic effect within the TME. Importantly, the negligible impact of CNCP on normal cells underscores the safety of this therapeutic strategy. By leveraging lactate metabolism reprogramming, the efficacy of photoimmunotherapy is significantly improved, offering valuable insights for the clinical translation of therapies under immunosuppressive conditions.

Given the high expression of MCT1 in the heart and eye tissues, the clinical use of its inhibitor, AZD3965, is constrained due to the risk of nonspecific drug distribution from oral administration. To enhance the therapeutic index of AZD3965, a targeted delivery system has been engineered using ultra-pH-sensitive micelle nanoparticles designed to selectively deliver the drug to the tumor microenvironment, thereby priming it for T-cell-mediated cancer therapy 123. These nanoparticles maintain their micellar structure at a neutral pH of 7.4, but disassemble and release their therapeutic payload in response to the acidic pH characteristic of the tumor microenvironment. This targeted delivery approach, termed AZD-UPS NP, when integrated with anti-PD-1 immunotherapy, has been shown to significantly suppress tumor growth and extend survival in two distinct tumor models. Notably, this nanoformulation achieves these therapeutic outcomes at doses of AZD3965 that are more than 200-fold lower than those required for oral administration, thus markedly expanding the drug's safety margin. Mitigating lactate metabolism instigates mitochondrial acidification, a process that is pivotal for inducing mitochondrial depolarization. Moreover, the upregulation of intracellular Ca^2+^ ions is identified as an alternative inducer of mitochondrial depolarization. To synergistically manipulate the H^+^/Ca^2+^ gradients and elicit mitochondrial depolarization, a supramolecular DNA nanocomplex has been meticulously engineered 124. This complex is fabricated through the strategic hybridization of bespoke DNA primers, encapsulating Fe/Mn-polyphenol coordination polymers within a siRNA (siMCT4)-integrated DNA nanonetwork. The nanoconstruct is subsequently biomineralized with Ca^2+^ in the presence of HA, yielding a Ca@DNA-MF nanoconstruct designed for tumor targeting. The HA-functionalized Ca@DNA-MF nanoconstruct exhibits enhanced accumulation at tumor sites, facilitating the targeted release of Ca^2+^ to perturb calcium homeostasis and initiate Ca^2+^-driven mitochondrial depolarization. In tandem, ATP-activated siMCT4 effectively downregulates MCT4 expression on the tumor cell membrane, curbing lactate efflux and instigating acidification of the TME. This TME acidification accelerates the sequestration of Ca^2+^ within the mitochondrial matrix, setting the stage for a heightened Fenton reaction and consequent ROS production. The pronounced cytoplasmic acidification, a consequence of lactate metabolism inhibition, significantly bolsters the Fenton reaction, leading to the generation of adequate ROS. This surge in ROS activity escalates mitochondrial membrane permeability, culminating in the dissipation of the proton gradient. This cascade is further propelled by the synergistic consumption of endogenous GSH through intricate redox mechanisms. Collectively, these orchestrated events result in metabolic reprogramming and the disruption of cancer cell homeostasis, culminating in efficient mitochondrial depolarization through the synergistic modulation of H^+^/Ca^2+^ gradients. This therapeutic strategy adeptly triggers apoptosis and ferroptosis, augmenting ROS generation and offering a robust synergistic therapeutic modality with minimized toxicity. The innovative approach paves the way for enhanced cancer therapies with reduced side effects, underscoring the potential of supramolecular DNA nanocomplexes in the realm of targeted cancer treatment. The efficacy of ferroptosis caused by iron-catalyzed lipid peroxides is hindered by the endogeneous lipid peroxide-scavenging mechanisms and the reliance on acidic pH. Cancer cells thrive in a slightly alkaline environment with a pH of 7.4 and rely on high levels of bioreductants like glutathione to maintain a rapid glycolysis rate, which is crucial for their survival and proliferation. However, this alkaline pH can reduce the solubility of active Fe^2+^ ions, hindering Fe^2+^-mediated lipid peroxidation, and high glutathione levels can counteract the cytotoxic effects of lipid peroxides through GPX4, diminishing the effectiveness of ferroptosis-based treatments. A novel nano integrated approach has been developed to overcome these challenges by combining clinically safe components to restructure glutathione and lactate metabolism in tumor cells, enhancing the efficacy of ferroptosis therapy 125. This involves the creation of nanoassemblies where ferrocene is attached to PEGylated polyamidoamine dendrimers via a ROS-cleavable thioketal bond, which also self-assembles with the GSH-depleting agent diethylmaleate (DEM) and MCT4 siRNA. These nanoassemblies can accumulate in tumors due to the EPR effect, are internalized by tumor cells, and then release their therapeutic components in the cytosol due to ROS-induced thioketal bond cleavage. DEM disrupts the GPX4 antioxidant defense by binding to GSH, and siMCT4 prevents lactic acid efflux, acidifying the intracellular environment and enhancing lipid peroxidation. This coordinated action increases intracellular H_2_O_2_ levels, creating a positive feedback loop for drug release. The nanoassembly synergistically inhibits tumor growth and extends the survival of mice with tumors through a ferroptosis mechanism, offering a promising strategy for clinical ferroptosis therapy development without significant side effects. Hu and colleagues have employed a comparable tactic to deplete lactate within the TME, concentrating lactate intracellularly, and subsequently decomposing it along with GSH to induce the production of ROS 126. To enhance the targeting specificity, disulfide bonds were strategically incorporated into the MOF backbone, capitalizing on their redox-responsive properties for selective accumulation in the reductive TME. Furthermore, the surface of liposomes was decorated with folic acid to augment tumor-targeting efficacy, leveraging the overexpression of folate receptors on many cancer cells to actively direct the therapeutic agents to the tumor site.

## 6.5 Reprogramming Metabolic Pathways

### 6.5.1 Glucose Metabolic Reprogramming of Cancer Cells

Cancer cells are well-known for adapting their metabolism to maintain high proliferation rates and survive in unfavorable environments with low oxygen and nutritional deficiency. Metabolic reprogramming most commonly arises from the tumor microenvironment (TME). The events of metabolic pathways include the Warburg effect, shift in Krebs cycle metabolites, and increase rate of OXPHOS that provides the energy for the development and invasion of cancer cells. In cancer cells, glucose metabolism often transitions from OXPHOS to a preference for glycolysis, a shift that enables rapid energy production essential for sustaining the rapid growth characteristic of cancer cells and ensuring their survival. This metabolic reprogramming can be leveraged therapeutically; specifically inhibiting glycolysis in tumor cells may restore metabolic balance, reduce the production of immunosuppressive lactate, and subsequently lower the levels of immune checkpoint proteins. However, a potential drawback is that inhibiting glycolysis could also affect immune cells, potentially diminishing the efficacy of immunotherapy. 122 The mTOR signaling pathway plays a pivotal role in the reprogramming of glucose metabolism in cancer cells by upregulating the expression of glucose transporters and glycolytic enzymes. In HCC and various other cancers, including pancreatic, breast, lung, and colorectal, mTOR signaling is frequently activated 127. This activation can induce the expression of key glycolytic genes such as GLUT1, HK2, and PKM2 through the action of transcription factors like HIF1α and MYC. Strategically inhibiting glycolysis in cancer cells, rather than in immune cells, could facilitate a reduction in checkpoint protein levels, making this a promising approach for enhancing the effectiveness of cancer immunotherapies. The challenge lies in the development of targeted therapies that can specifically disrupt glycolysis in cancer cells while sparing the metabolic needs of immune cells engaged in tumor rejection. Some nanomedicines aim to reprogram cancer cell metabolism by promoting OXPHOS over glycolysis, a process known as metabolic reprogramming. This can be achieved by delivering molecules that enhance mitochondrial function or by inhibiting glycolytic intermediates, forcing cancer cells to rely more on OXPHOS for ATP production. Since cancer cells are often less efficient at OXPHOS compared to normal cells, this shift can lead to energy deficiency and cell death. While molecular drugs that target glycolysis have been utilized to curb the energy production of cancer cells through glycolysis, the majority of these single-targeting molecular drugs or nanoparticles have struggled to effectively counteract the metabolic adaptability, or "metabolic plasticity," of cancer cells. Consequently, there is an urgent need for innovative therapeutic agents that can comprehensively suppress both glycolysis and cellular respiration to effectively thwart the "metabolic reprogramming" exhibited by cancer cells, thereby leading to more successful cancer treatments. The development of such agents is essential to address the limitations of current therapies and to provide a more robust and effective approach to cancer management.

A simultaneous dual blockade of glycolysis and OXPHOS can disrupt the metabolic adaptability of cancer cells, thereby severing their primary energy sources and paving the way for effective cancer treatment. However, the disparate pharmacokinetic profiles of different drugs make it challenging to achieve a synchronized inhibition of both processes in a coordinated manner. In this context, Lu group introduce an aptamer-based artificial enzyme that enables concurrent dual inhibition of glycolysis and OXPHOS 128. This system, referred to as AptCCN, is engineered by conjugating arginine aptamers with carbon-dots-doped graphitic carbon nitride. AptCCN exploits the aptamers' specific recognition of arginine to concentrically sequester this amino acid within cells, and subsequently catalyzes the conversion of the accumulated arginine into nitric oxide (NO) under red light exposure. Both *in vitro* and *in vivo* studies have demonstrated that the combined depletion of arginine and the introduction of NO stress can effectively suppress glycolysis and OXPHOS, leading to an energy crisis and the subsequent apoptosis of cancer cells. This aptamer-based artificial enzyme represents a novel approach to modulating cellular pathways and offers a synergistic strategy for cancer therapy.

In contrast to the suppression of glycolysis, the enhancement of glucose imports and glycolysis by treatment with a Met and NF-κB inhibitor drives lactate production to significantly higher levels. In tandem with redirecting glycolytic end-products from the TCA cycle, this approach efficiently redirects metabolic energy flow, leading to an increased yield of lactate 129. The consequent sequestration of lactate within the cell, exacerbated by the inhibition of its export via MCT4, results in intracellular lactate accumulation and a concomitant reduction in intracellular pH, culminating in substantial cytotoxic effects. NF-κB is a pivotal regulator of energy homeostasis and metabolic adaptation, accomplishing these roles through the upregulation of mitochondrial respiration 130. The inhibition of NF-κB induces a cellular metabolic reprogramming towards aerobic glycolysis under standard conditions and incites necrotic cell death in scenarios of glucose scarcity. This metabolic reconfiguration, prompted by NF-κB inhibition, circumvents the necessity for mutations in tumor suppressor genes, a common requirement in oncogenic transformation, and attenuates the metabolic adaptability of cancer cells in physiological settings. Relative to scenarios with normal glucose levels, a nanosystem-based therapeutic approach demonstrates heightened potency in the elimination of cancer cells within a high-glucose environment. Corresponding animal model studies have illustrated that diabetes mellitus conditions are conducive to breast cancer xenograft proliferation and progression in immunodeficient mice. Moreover, the application of MRS treatment has been shown to markedly repress the progression of breast cancer induced by high glucose conditions.

Although the simultaneous disruption of OXPHOS and glycolysis through the combination of mitochondrial-targeting PDT and glycolysis inhibitors (Sy) promises synergistic and lethal energy exhaustion in tumor cells, the efficacy of such combination therapies is often hindered by the inadequate co-delivery and suboptimal tumor targeting of conventional drug delivery systems. To overcome these limitations, cancer cell membrane biomimetic nanoparticles have been developed. These nanoparticles can evade immune clearance and accurately target similar tumor cells *in vivo*, thereby enhancing the lethal energy depletion effect. In a groundbreaking study, pyropheophorbide a (PPa) was linked with triphenylphosphine to create a mitochondrial-targeting photosensitizer (TPPa). Dong and colleagues developed cancer cell membrane-coated poly(lactic-co-glycolic acid) (PLGA) nanoparticles that encapsulated both TPPa and Sy 131. These nanoparticles demonstrated a high degree of precision in targeting tumors. Once inside the tumor cells, TPPa localized to the mitochondria and, upon laser irradiation, produced a substantial amount of ROS, leading to a closed-loop that exacerbated OXPHOS inhibition. Most significantly, the OXPHOS inhibition triggered by TPPa synergized with the glycolysis inhibition initiated by Sy, effectively severing the dual energy pathways that fuel cancer cells and leading to a synergistic and lethal depletion of cellular energy.

In glucose metabolism, pyruvate dehydrogenase kinases (PDKs), a rate-limiting enzymes in glycolysis, acts as a metabolic gatekeeper by inhibiting pyruvate dehydrogenase, thereby preventing the conversion of pyruvate to acetyl-CoA and obstructing its entry into the tricarboxylic acid cycle for energy production. PDKs are instrumental in modulating glycolysis, serving as key regulators in the metabolic balance of cellular energy production. Pyruvate dehydrogenase kinase 1 (PDK1) was overexpressed in tumors, which could be employed as molecular target to reverse immunosuppression in tumor microenvironment. PDK inhibitors have shown potential by activating the pyruvate dehydrogenase complex (PDC), redirecting cellular metabolism towards OXPHOS, and inducing apoptosis in cancer cells.

A mitochondrial kinase inhibitor, dichloroacetate (DCA) was used to change metabolic pathways in cancer cells for inhibiting the proliferation of cancer cells as well as activating immune system. Therefore, combination of inhibitor of glycolysis and checkpoint elicit effect in the treatment of cancer. However, high dose of DCA was needed to achieve a therapeutic effect. DCA significantly improved tumor clearance in conjunction with standard chemoradiation among immune-competent mice, a benefit not observed in their immune-compromised counterparts 132. Strategies such as nanoformulation as well as modification of DCA for mitochondrial targeting was used to modulate TME and to upregulate tumor infiltrating lymphocytes, yield a decrease in tumor volume.

A significant inverse relationship has been identified between the expression levels of PD-L1 and PDK in gastric cancer cells 133. The inhibition of PDK has been shown to enhance histone acetylation through the promotion of acetyl-CoA production, a critical epigenetic modification that influences gene expression. This increase in acetyl-CoA, along with the inhibition of histone deacetylation, has been linked to an upregulation of PD-L1 expression, suggesting a novel regulatory mechanism by which PDK modulates PD-L1 expression through histone acetylation. Furthermore, the anticancer efficacy of PD-L1 blocking antibodies was significantly amplified following treatment with a PDK inhibitor. This finding indicates that by impeding tumor aerobic glycolysis, PDK inhibition augments the effectiveness of PD-L1 immunotherapy. The enhancement of glycolysis-induced histone acetylation via acetyl-CoA elevation, and the subsequent modulation of PD-L1 by PDK, present a compelling rationale for targeting PDK as a strategy to augment the efficacy of immune checkpoint blockade (ICB) therapy with PD-L1 antibodies. This approach holds the potential to offer substantial benefits for patients with advanced gastric cancer, providing a new avenue for therapeutic intervention in this challenging disease setting. To amplify mitochondrial oxidative stress, a systemically administrable and efficient delivery system that targets mitochondria would be advantageous. Tang *et al.* have develop that poly[2-(N-oxide-N, N-diethylamino)ethyl methacrylate] (OPDEA), a water-soluble zwitterionic polymer based on a tertiary amine-oxide, is capable of mitochondrial targeting and remains stable in the circulatory system. Building on this, they have engineered a mitochondria-targeted amphiphilic block copolymer, OPDEA-b-PDCA, which self-assembles into stable micelles (OPDEA-PDCA) for the selective suppression of PDK1 134. Within this construct, OPDEA serves the purpose of mitochondrial targeting, while the conjugated DCA is utilized to inhibit PDK1 activity. The specific inhibition of PDK1 by OPDEA-PDCA can lead to an increase in OXPHOS and a consequent rise in oxidative stress, potentially initiating a process of programmed cell death. OPDEA-PDCA is effective in inducing mitochondrial oxidative stress, which subsequently triggers pyroptotic cell swelling and rupture of the cell membrane. Additionally, the proptosis induced by OPDEA-PDCA has been identified as a form of ICD, characterized by the release of multiple immunostimulatory factors, including interleukin-1β (IL-1β) and high mobility group box 1 (HMGB1). This release contributes to the creation of a pro-inflammatory tumor microenvironment. Furthermore, we observed the release of soluble programmed cell death-ligand 1 (PD-L1) from the pyroptotic osteosarcoma cells. While the membrane-bound form of PD-L1 typically interacts with PD-1 on T cells to inhibit their activation, the soluble form of PD-L1 has been found to facilitate immune evasion and T cell exhaustion. In light of this, the combined use of OPDEA-PDCA with an anti-PD-L1 monoclonal antibody was explored to counteract T cell exhaustion. This combined therapeutic approach demonstrated significant antitumor effects against osteosarcoma, leading to substantial tumor regression. The compromised stability of DCA in biological contexts and the anionic charge of its ionized molecules are key factors that can substantially diminish its therapeutic effectiveness. The anionic charge restricts DCA's ability to accumulate across the hyperpolarized mitochondrial membrane, resulting in rapid *in vivo* clearance and preventing the molecule from reaching tumor cells. This also impedes its capacity to traverse the cell membrane and access the mitochondria through passive diffusion. To surmount these obstacles, a triphenylphosphonium (TPP) cation has been integrated to enhance mitochondrial membrane penetration 135. Concurrently, a dimethyloctadecyl silane moiety has been introduced to function as a hydrophobic, extended silane chain. This modification not only bolsters the compound's stability by shielding it from physiological hydrolysis but also augments its overall therapeutic potential. Elevated PDK1 expression has been detected in patients with resistance to Osimertinib, a third-generation EGFR tyrosine kinase inhibitor (TKI) that selectively targets the EGFR T790M mutation in non-small cell lung cancer (NSCLC) 136. The emergence of EGFR C797S mutant cells, characterized by increased EGFR phosphorylation and activation of oncogenic pathways, has been linked to an upregulation of PDK1-mediated glycolysis via the EGFR/AKT/HIF-1α signaling axis. The combination of DCA, a PDK inhibitor, with first-generation EGFR-TKIs like erlotinib and gefitinib has shown promising results, and the synergistic potential of DCA with the third-generation EGFR-TKI rociletinib, alongside radiation therapy, has been demonstrated in NSCLC cell line models. However, the clinical utility of DCA is limited by its requirement for high doses. Leelamine, a more selective PDK inhibitor, has emerged as a more targeted therapeutic agent. The combination of osimertinib with leelamine has been effective in overcoming osimertinib resistance in allograft models, offering a novel and potentially transformative therapeutic strategy for NSCLC patients. This approach underscores the importance of precision medicine in addressing drug resistance and highlights the role of PDK1 inhibition in enhancing the efficacy of EGFR-targeted therapies. High levels of PDK2 have been identified in chemoresistant ovarian cancer tissues and cell lines, correlating with shorter progression-free survival 137. PDK2 knockdown has been shown to enhance oxygen consumption rates and reduce extracellular acidification rates, indicative of a metabolic shift from glycolysis to OXPHOS. This metabolic reprogramming is accompanied by decreased lactate production, increased PDH activity, and elevated levels of electron transport chain complexes III and V, which collectively contribute to the restoration of cisplatin sensitivity in ovarian cancer cells. While DCA exhibits sensitivity to PDK2 and holds promise for augmenting cisplatin sensitivity, its clinical translation is impeded by its limited efficacy in suppressing tumor growth and the associated systemic toxicity. The broad tissue distribution of PDK2 necessitates precise targeting for effective clinical use, prompting the exploration of RNAi as a tool for *in vitro* PDK2 gene silencing. A novel approach involves the use of FSH receptor-mediated nanocarriers for *in vivo* delivery of PDK2 shRNA, leveraging the FSH receptor's specific expression in ovaries as a targeting site for ovarian cancer. The FSH β 33-53 peptide, which binds specifically to the FSH receptor, has been re-engineered with D-amino acids to enhance stability and binding affinity, overcoming the limitations of natural peptides susceptible to protease degradation. By conjugating the retro-inverso FSH peptide with PEG-PEI and loading it with PDK2 shRNA, a targeted gene delivery system has been developed. The combined treatment with this system and cisplatin significantly delayed the growth of chemoresistant ovarian cancer, demonstrating the potential of PDK2 inhibition to redirect metabolism from glycolysis to OXPHOS, thereby suppressing tumor growth and enhancing the therapeutic efficacy of chemotherapy. Furthermore, the Warburg effect, mediated by PDK1 and PDK2, plays a crucial role in the TGFβ1-induced enhancement of stem-like properties in head and neck cancer (HNC) 138. Both HNC cancer stem cells (CSCs) and tumor cells predominantly rely on aerobic glycolysis to rapidly acquire energy and the necessary macromolecular precursors essential for their continuous and uncontrolled proliferation.

The enhanced metabolic plasticity observed in brain tumors, particularly those originating from peripheral tumors like breast cancer, is marked by increased glycolytic and tricarboxylic acid cycle flux, and a robust capacity to switch between OXPHOS and glycolysis. To address the unique challenges of targeting both peripheral and brain tumors, including the impermeableBBB and the need for precise drug ratios and pharmacokinetics, Ashokan has engineered a novel nanoparticle system 139. These nanoparticles, based on poly(lactic-co-glycolic acid)-block-polyethyleneglycol (PLGA-b-PEG) copolymers, are functionalized with a terminal TPP cation that enables efficient crossing of the BBB and the mitochondrial double membrane. The TPP cation capitalizes on the substantial hyperpolarized mitochondrial membrane potential to effectively target mitochondria. This mitochondria-targeted, BBB-penetrating nanoparticle system, along with the EPR effect, allows for targeted drug delivery to the hyperpolarized mitochondrial membrane of cancer cells at both the primary and metastatic sites. By incorporating therapeutic agents such as Platin-M and Mito-DCA, these nanoparticles can disrupt the metabolic adaptability of tumors and initiate chemotherapeutic effects by forming repair-resistant platinum adducts with mitochondrial DNA. The approach presents a promising therapeutic strategy for brain tumors, offering a meaningful clinical option that could significantly impact the treatment of metastatic cancer to the brain. Additionally, Xie and colleagues have synthesized silver nanoparticles with a size range of 9.38±4.11 nm, which have been shown to selectively trigger apoptosis in osteosarcoma cells via a ROS-dependent mechanism, sparing normal cells 140. These silver nanoparticles demonstrated excellent stability in various biological environments and aqueous solutions. They selectively altered the glucose metabolism of osteosarcoma cells from glycolysis to mitochondrial oxidation by inhibiting PDK. The half-maximal inhibitory concentration (IC_50_) values indicated that the 143B cell line (IC50=2.92 ± 1.06 ng/μL) was the most sensitive to the silver nanoparticles among the tested osteosarcoma cell lines. The silver nanoparticles also displayed a significantly higher tumor inhibition ratio (49.19%) compared to cisplatin (14.53%) on the 21st day of treatment. In comparison to cisplatin, mice treated with the silver nanoparticles did not exhibit significant weight changes, nor did they show any alterations in the gross appearance or histological structures of vital organs such as the lung, heart, liver, spleen, and brain. These findings suggest that the silver nanoparticles have a lower toxicity profile than cisplatin in mice bearing osteosarcoma. The treated mice maintained similar or higher body weights and daily food and water intake, indicating a high level of tolerability for the silver nanoparticles at effective therapeutic doses. When osteosarcoma cells were incubated with the silver nanoparticles, there was an initial metabolic shift from glycolysis to glucose oxidation, which eventually led to mitochondrial dysfunction after prolonged exposure. In contrast, the metabolism and mitochondrial function of normal cells remained largely unaffected by the silver nanoparticles. This selective effect is attributed to the activation of pyruvate dehydrogenase (PDH) through the inhibition of PDK, facilitated by the ROS generated in the presence of the silver nanoparticles.

Diabetes is intricately linked to the development and poor prognosis of endometrial cancer (EC), with a 41% increased mortality rate among diabetic EC patients. The metabolic reprogramming in EC with diabetes, marked by enhanced glycolysis and reduced OXPHOS, contributes to metabolic resistance in EC cells. PDK is identified to promote glycolysis in EC cell lines cultured in high glucose, and its inhibition using short hairpin RNA can significantly suppress proliferation, invasion, glycolysis, and the AKT/GSK3β/β-catenin signaling pathway 141. JX06, a PDK1 inhibitor, when combined with metformin, accelerates apoptosis in diabetic EC. To overcome the challenges of JX06's poor water solubility and rapid blood clearance, it has been encapsulated in GSH-responsive biodegradable polymer nanoparticles (JX06-NPs), which show enhanced inhibitory effects on high-glucose EC cells. These JX06-NPs circulate in the blood, accumulate at tumor sites, and release JX06 to inhibit PDK1, while metformin, administered orally, regulates glucose levels and inhibits mitochondrial complex I to suppress OXPHOS in tumor cells. The combination of JX06-NPs and metformin has been shown to increase apoptosis in EC cells, offering a promising new strategy for the treatment of endometrial cancer in patients with concurrent diabetes.

Cancer cells often upregulate the expression of key glycolytic enzymes, such as GLUT1, HK2, and MCT1, which are implicated in the mammalian target of rapamycin (mTOR) signaling pathway. This activation is crucial for sustaining the clonality of cancer cells and facilitating their migration. The mTOR pathway is well-known for its role in promoting mRNA transcription, protein synthesis, glucose metabolism, and lipid synthesis-all of which are essential for cell growth and proliferation. In -HCC, CD147 has been shown to stimulate glycolytic metabolism through the PI3K/Akt/mTOR signaling pathway, contributing to the immunosuppressive phenotype of lymphocytes 142. Similarly, ILT4 induces the reprogramming of aerobic glycolysis in tumor cells by mediating the overexpression of GLUT3 and PKM2 via the AKT-mTOR signaling pathway 143. Research has also highlighted the role of miR-21 in regulating aerobic glycolysis in bladder cancer cells through the PTEN/PI3K/AKT/mTOR pathway. Inhibition of miR-21 resulted in a decrease in aerobic glycolysis, suggesting its potential as a therapeutic target 144. The interplay between the upstream and downstream components of the PI3K/AKT/mTOR signaling pathway is a significant regulator of cancer cell glycolysis 145. For instance, the knockdown of Forkhead box protein O (FOXO)6, a member of the FOXO family of transcription factors, has been shown to inhibit the activation of the PI3K/AKT/mTOR pathway. This inhibition leads to a shift in cellular metabolism by reducing glycolysis and enhancing mitochondrial respiration in colorectal cancer (CRC). These findings indicate that FOXO6 could be a promising mTOR-dependent therapeutic target for CRC treatment.

### 6.5.2 Glucose Metabolic Reprogramming of Stromal Cells

Metabolic reprogramming, a hallmark of cancer, extends beyond tumor cells to encompass the TME, representing a significant and burgeoning avenue of oncological research 146. The TME, a complex ecosystem comprising blood and lymphatic tumor vessels, extracellular matrix (ECM), and various non-cancerous stromal cells, exerts a profound influence on the behavior of cancer cells, including their growth, progression, and resistance to therapeutic interventions. Within this intricate milieu, cancer-associated fibroblasts (CAFs) emerge as a predominant cellular constituent, playing a central role in modulating the capacity of TME to foster tumorigenesis and impede the effectiveness of cancer treatments. Cancer cells highjack CAFs and reprogram their metabolism to perform aerobic glycolysis. CAFs, consequently, secrete metabolites such as pyruvate and lactate, which are taken up by cancer cells and support their metabolic needs for the rapidly proliferating tumor cells. Breast cancer CAFs are particularly reliant on MCT4 to sustain the export of lactate into TME, thereby facilitating the metabolic reprogramming of tumor cells 147. The miR-425-5p, when induced to knockdown MCT4, diminishes lactate extrusion from CAFs, limiting its availability within the TME. Overexpression of miR-425-5p downregulates MCT4 levels in breast cancer CAFs, effectively curbing lactate efflux. Cancer exosomal miR-105 stimulates the MYC signaling cascade in CAFs, thereby enhancing the catabolism of glucose and glutamine 148. TGF-β activation of CAFs induces metabolic reprogramming, oxidative stress, and autophagy, while also modulating the expression of proteins like Cav-1 149. miR-424 orchestrates a metabolic shift in CAFs by downregulating isocitrate dehydrogenase 3 alpha (IDH3α), a key enzyme in the tricarboxylic acid (TCA) cycle, promoting metabolic reprogramming to support glycolysis 150. Epigenetic modifications of HIF-1 α and glycolytic enzymes 151, SNHG3^152^, migration stimulating factor 153, hyperglycaemia 154, estradiol-17 β^155^ and lysophosphatidic acid^156^ have been shown to significantly redirect glucose metabolism in CAFs, resulting in an enhanced production of lactate and pyruvate, which not only reflects a shift toward anaerobic glycolysis but also provides essential substrates for the biosynthetic pathways that support the growth and proliferation of cancer cells. In TME, immune cells are often subjugated by the metabolic machinations of cancer cells and CAFs. Elevated lactate secreted by these cells dampens immune cell functionality, fostering an immunosuppressive landscape. Targeting this metabolic crosstalk, a hybrid biomimetic camouflage delivery system has been crafted to encapsulate the chemotherapeutic agent paclitaxel and the glycolysis inhibitor PFK15 157. By fusing cancer and fibroblast cell membranes, the system harnesses their native targeting prowess for dual-targeting capability, homing in on both cancer cells and CAFs. These hybrid cell membrane-coated nanoparticles effectively inhibit glycolysis, thwarting energy production and starving cancer cells of glycolytic metabolites supplied by CAFs. Moreover, by reducing lactate secretion, these nanoparticles invigorate the TME's immune cells, reversing the suppressive state and bolstering the cytotoxic impact of PTX. The synergistic effect of PTX and PFK15, encapsulated within this innovative delivery system, not only curbs tumor growth but also incites immune responses, showcasing the potency of metabolic reprogramming in the arsenal of cancer therapies.

The paradoxical nature of the TME, which concurrently fosters tumor cell metabolism while inhibiting lymphocyte metabolism, underscores the pivotal role of metabolic adaptations in dictating cellular fate. This phenomenon highlights the immense potential of metabolically modulating immune cells to overcome suppression and immune evasion within the TME, thereby enhancing their therapeutic efficacy. Tumors can be categorized into three distinct types based on their immune microenvironment status: inflammatory or hot tumors, immune inhibitory tumors, and immune escape or cold tumors 158. In immune inhibitory tumors, the immune microenvironment is dominated by bone marrow-derived inhibitory immune cells, leading to an inactivation of the adaptive immune response initiated by DCs. Conversely, in cold tumors, immune cells are unable to infiltrate the TME due to the absence of major histocompatibility complex (MHC) class I molecules on tumor cells or a lack of specific tumor-associated antigens presented by MHC-I. As a result, immunotherapy often fails to elicit the desired response in these cases. Within TME, a diverse array of tumor-infiltrating immune cells, including T-cells, Tregs, MDSCs, tumor-associated neutrophils, dendritic cells, and notably, TAMs, coalesce to influence cancer dynamics 159. TAMs, being the most abundant, are pivotal in promoting cancer metastasis through the secretion of extracellular signals, growth factors, proteolytic enzymes, and T cell inhibitory proteins. A novel study has strikingly revealed that TAMs, rather than cancer cells, are the predominant consumers of glucose in cancers, fulfilling their energy demands by upregulating the GLUT1 on their surface. Under normal conditions, naive M0 macrophages primarily utilize OXPHOS for energy production, whereas polarized M1 and M2 macrophages depend on distinct metabolic pathways reflective of their tissue microenvironment. M1 macrophages, with their proinflammatory functions, engage in aerobic glycolysis and the pPPP to meet their heightened energy needs, while reducing OXPHOS and fatty acid oxidation. These metabolic pathways are crucial for macrophage functional modulation and for averting harmful events during emergencies. HIF-1α, a pivotal regulator, has been demonstrated to stimulate glycolysis by upregulating the expression of lactate dehydrogenase and pyruvate dehydrogenase kinase 160. This metabolic shift facilitates the production of the proinflammatory cytokine IL-1β. Furthermore, M1 macrophage polarization is characterized by disruptions in the tricarboxylic acid cycle (TCA cycle), a pronounced enhancement of glycolysis, and alterations in OXPHOS, all of which contribute to the inflammatory phenotype. In contrast, alternatively activated M2 macrophages, induced mainly by IL-4 and IL-13 secreted from innate and adaptive immune cells, exhibit an anti-inflammatory phenotype characterized by IL-10 and transforming growth factor-β (TGF-β). Unlike M1 macrophages, M2 macrophages predominantly use FAO and OXPHOS for their cellular activities. Cancer cells, typically glucose-dependent, consume most of the glucose in the surrounding microenvironment to fuel their rapid growth through glycolysis, which consequently pushes TAMs to rely more on OXPHOS and FAO in a nutrient-poor TME, thereby sustaining their immunosuppressive functions. Given the critical role of TAMs in tumor promotion and the heterogeneity that evolves under TME selective pressures, modulating macrophage metabolism emerges as a promising therapeutic strategy. Since macrophage glucose metabolism is intrinsically linked to their functionality, metabolic reprogramming of M2-like TAMs to an anti-tumoral phenotype, while disrupting cancer cell metabolism, presents an elegant therapeutic opportunity. In light of the close relationship between OXPHOS and M2 macrophage differentiation, particularly in TAMs, inhibiting the OXPHOS pathway has been explored as a means to encourage the transition of TAMs to the M1 phenotype. Targeting the succinate dehydrogenase complex flavoprotein subunit A and the OXPHOS activities of macrophages with dimethyl malonate treatment has shown significant tumor growth delay, underscoring the potential of metabolic interventions in redirecting macrophage polarization and impeding cancer progression.^3^

**Figure 6:**
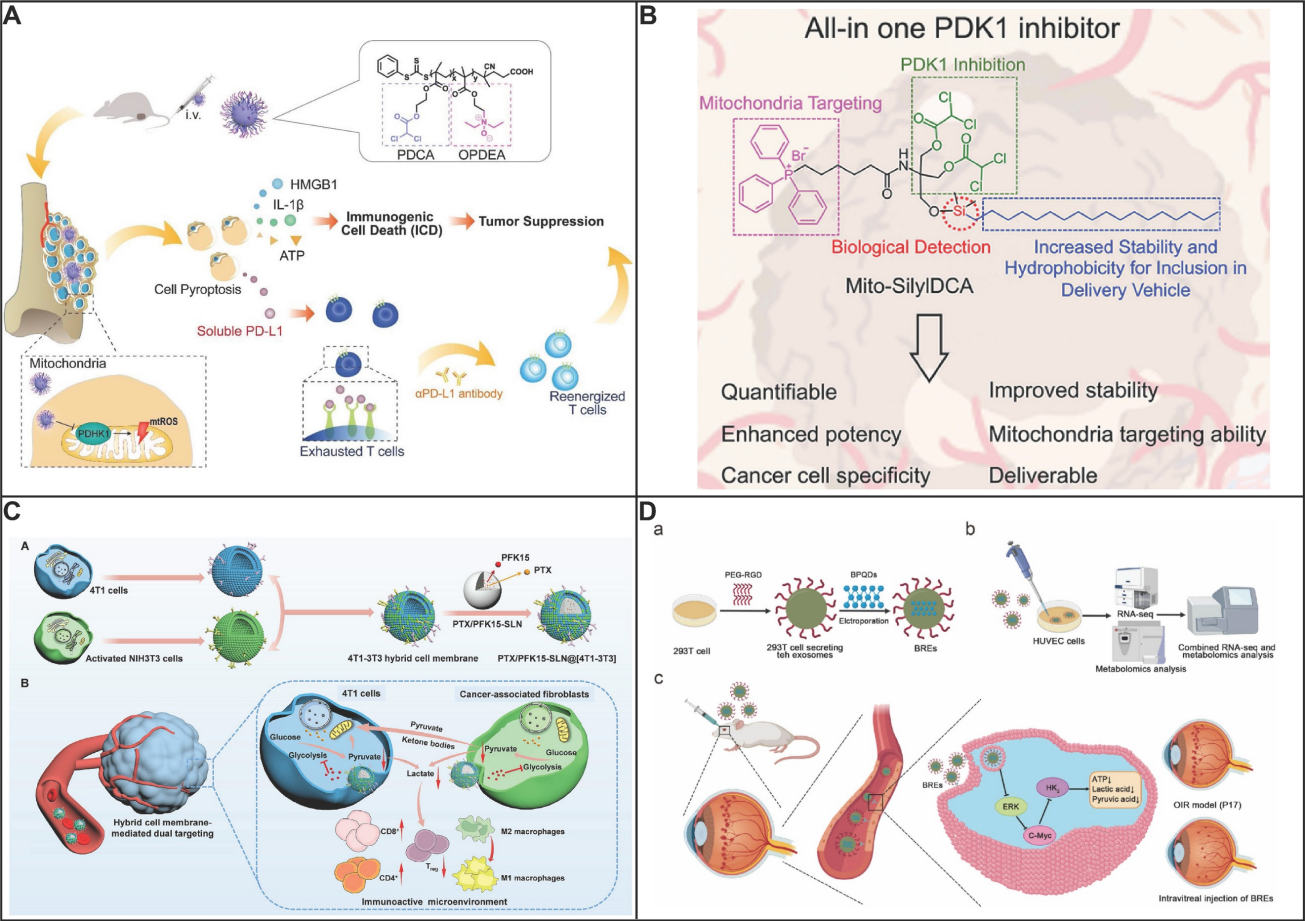
Reprogramming metabolic pathways. (A) Schematic diagram showing cell proptosis induced by mitochondria-targeted nanodrug in osteosarcoma and the mechanism of the enhanced antitumor effect triggered by combined therapy with nanodrug and an anti-PD-L1 monoclonal antibody. Reproduced with permission from ref 134. Copyright 2022 American Chemical Society. (B) Schematic diagram showing pyruvate dehydrogenase kinase inhibitor for tracking, targeting, and enhanced efficacy. Reproduced with permission from ref 135. Copyright 2023 American Chemical Society. (D) Schematic diagram showing the preparation and application of BPQDs@RGD-EXO. Reproduced with permission from ref 182. Copyright 2023 Elsevier.

It is clearly distinct from the metabolic pathways of cancer cells, M2 macrophages are drawn to hypoxic regions within tumors, where glycolysis is subdued, and OXPHOS is favored due to the activation of the IRE1-XBP1 pathway. This metabolic shift leads to the accumulation of intracellular lipids, which in turn sustains the M2 phenotype. Energy for M2 macrophages is derived from both OXPHOS and FAO. Historically, driving the polarization from M1 to M2 was seen as a breakthrough in antitumor therapy by modulating the IRE1-XBP1 pathway. However, the hypoxic tumor microenvironment restricts the effectiveness of M2 repolarization, and the abundance of ROS in the TME further impedes this process. Effective repolarization of M2 macrophages to M1 requires a metabolic reprogramming that enhances glycolysis and curtails FAO. M2-TAM, a critical component of the TME, contribute to tumor tolerance to anti-PD-1 therapy by upregulating molecules such as triggering receptor expressed on myeloid cells 2 and IDO. Metabolic reprogramming of macrophages is, therefore, essential for overcoming tumor resistance to anti-PD-1 therapy. Jiang and colleagues have developed a nanoemulsion containing KIRA6, an inhibitor of the IRE1-XBP1 pathway, and α-tocopherol, a ROS inhibitor, to facilitate the repolarization of M2-TAMs to M1 macrophages (Figure 6B) 161. This innovative approach significantly increased the M1/M2 ratio under both normoxic and hypoxic conditions, bolstered lymphocyte proliferation, and curbed tumor cell growth, independent of the hypoxic TME. The treatment repressed fatty acid metabolism in macrophages and redirected glucose metabolism from OXPHOS to glycolysis, leading to alterations in key transcription factors (STAT1 for M1 macrophages and STAT3 for M2 macrophages), and ultimately achieved the repolarization of M2 cells to M1. The presence and cytotoxic function of CD8^+^ T cells in the tumor microenvironment were increased following the macrophage switch to M1, thereby constraining tumor progression. The nanoemulsion developed by this team offers the advantages of simple composition, low cost, straightforward preparation, and robust storage stability, indicating its significant potential for clinical translation and application. 2-DG impedes glycolysis in M2 TAMs, significantly reducing the polarization of anti-inflammatory M2 macrophages 162. Furthermore, DCA has been shown to markedly inhibit macrophage migration in a lung tumor xenograft model, an effect attributed to the suppression of glycolysis in macrophages 163. While several inhibitors of O-GlcNAcylation have demonstrated the ability to hinder cancer cell growth, the targeted disruption of a single metabolic pathway in macrophages may be an evasive strategy 164, 165. It suggests that a multifaceted approach to metabolic intervention could be more effective, considering the complex metabolic network within the tumor microenvironment and the plasticity of macrophage phenotypes.

DCs, as crucial antigen-presenting cells, are tasked with capturing antigens from pathogens or tumors and presenting them to T cells, initiating an immune response. The metabolism of DCs intricately governs their development, polarization, maturation, and functional performance, providing the energy required for these processes. Aberrant metabolism within tumor cells triggers alterations in the TME, including heightened glycolysis, lactate and lipid accumulation, acidification, and tryptophan depletion. These changes impede DC function, fostering tumor immune evasion. Activated DCs rely heavily on glycolysis and the PPP for energy production, membrane integrity maintenance, inflammatory mediator synthesis, and migration capabilities 166. Disrupting glycolysis impairs DC functions, including antigen presentation, cytokine secretion, and T-cell activation. The activation of multiple T cells by DCs leads to intense nutrient consumption, particularly glucose, which can disrupt DC glucose availability. This competitive glucose uptake by T cells modulates DC metabolism, inactivating mTORC1/HIF-1α/NOS2 signaling and promoting the generation of pro-inflammatory DCs to amplify T-cell responses. HIF-1α serves as a potent regulator of glycolysis by enhancing the expression of key glycolytic genes, including GLUT1 and LDH, to augment energy production. Upon TLR4 activation in monocyte-derived dendritic cells (moDCs), glycolysis is sustained through an intricate mechanism involving the p38-mitogen-activated protein kinase (MAPK) pathway. This pathway triggers HIF-1α accumulation, which in turn stimulates the activity of HK2, the initial rate-limiting enzyme of glycolysis, thereby facilitating the metabolic reprogramming of DCs to meet their energetic demands 167. Lactate accumulation in the TME further inhibits DC activation and antigen expression. Enhancing DC vaccines through targeting the highly expressed mannan receptor on antigen-presenting cells (APCs) offers a promising avenue for improving DC efficacy. By conjugating tumor antigens to mannan, DCs can more precisely target tumor sites, enhancing IFN-γ production and promoting the generation of antigen-specific CD8^+^ T cells. Additionally, a novel DC subgroup, merocytic DCs (mcDCs), characterized by high CD11c expression and deficiency in CD8a and CD11b, has emerged. Derived from pre-conventional DCs (cDCs), mcDCs co-express ZBTB46 and IFN regulatory factor 4 (IRF4) 168. Notably, mcDCs exhibit reduced glucose ingestion and glycolysis dependence compared to cDC1 and cDC2 subsets, conferring upon them the unique ability to reverse T-cell dysfunction. These findings underscore the complex interplay between DC metabolism, TME alterations, and immune responses in tumor biology.

Diverse T-cell subsets, including T helper cells (Th), Tregs, cytotoxic T cells (TCs), memory T cells, NK cells, and gamma delta (γδ) T cells, exhibit distinct metabolic profiles that govern their differentiation and tumor-specific responses. Notably, CD4^+^ Th cells and CD8^+^ Teff cells rely heavily on glycolysis, whereas Tregs primarily utilize FAO for their metabolic needs 169. These metabolic pathways are crucial regulators of T-cell function in the TME. While NK cells are instrumental in anti-tumor immunity through direct cytotoxicity and cytokine-mediated immune orchestration, their dysfunction within the hostile TME poses a significant obstacle to cancer immunotherapy, particularly against solid tumors. The TME suppresses NK cells, enabling tumor escape and progression. NK cells' primary metabolic fuel is glucose, which fuels their anti-tumor response through enhanced glycolysis and mitochondrial OXPHOS. Recent evidence suggests that NK cell activation and memory formation are accompanied by profound metabolic reprogramming. Activated NK cells shift their metabolism from FAO to glycolysis and OXPHOS. However, elevated expression of fructose-1,6-bisphosphatase (FBP1) in NK cells impairs their glycolytic capacity 170. Moreover, the TME inhibits NK cell glucose metabolism primarily through lipid-peroxidation-induced oxidative stress, further hindering their anti-tumor efficacy 171. Srebp facilitated elevated glycolysis and OXPHOS, fostering a distinctive metabolic pathway that channeled glucose into cytosolic citrate via the citrate-malate shuttle 172. Thus, overcoming these metabolic barriers in NK cells holds promise for advancing cancer immunotherapy strategies. Future research should delve deeper into the metabolic disparities between cancer and immune cells within the TME, informing refined strategies for metabolic regulation-based therapies.

Neutrophils, though pivotal in immune defense, paradoxically contribute to tumor progression in cancer by generating ROS that disrupt lymphocyte function. Traditionally overlooked for their metabolic diversity, neutrophils in glucose-deprived tumor microenvironments adapt metabolically, transitioning to oxidative metabolism to sustain local immune suppression 173. Loss of Glut1 accelerated neutrophil turnover in tumors, diminishing a SiglecF-expressing neutrophils subset, thereby inhibiting tumor growth and enhancing the effectiveness of radiotherapy 174. Elevated HIF-1α and LDHA levels in neutrophils led to glucose metabolism reprogramming, thereby suppressing their immune effector functions 175.

Angiogenesis, the vital process of forming new blood vessels, plays a pivotal role in tumor development by facilitating the delivery of essential nutrients and oxygen to cancer cells while efficiently removing metabolic waste. This crucial process significantly contributes to the growth, progression, invasion, and metastasis of tumors, underlining its significance in cancer biology. Vascular endothelial cells (ECs) undergo phenotypic changes during tumor growth, shifting to a glycolytic-dominant metabolism even in aerobic conditions. This altered metabolism, characterized by heightened glycolytic flux and lactate utilization, fuels tumor growth, metastasis, and resistance to chemotherapy. Tumor vessel normalization, achieved through targeting glycolytic enzymes like PFKFB3 176, PKM2 177, TRPM7 178 and PDK 179 emerges as a promising anticancer strategy by improving vessel maturity and perfusion, thereby reducing tumor aggressiveness and enhancing chemotherapy effectiveness. Bladder cancer-derived exosomes reprogram glucose metabolism in ECs by augmenting the flux through the HBP 180. This metabolic shift culminates in enhanced O-GlcNAcylation, a post-translational modification process that particularly targets serine-tRNA synthetase, thereby fostering the aggressive expansion and neovascularization of bladder cancer. Furthermore, lactate, abundant in the milieu of tumor-associated endothelial cells (TECs), inhibits the activity of prolyl hydroxylase-2 (PHD-2), thereby enabling the activation of the NF-κB signaling pathway 181. This intricate interplay underscores PHD-2's pivotal role in orchestrating angiogenesis and the maturation of neovessels, achieved through its regulation of NF-κB, HIF-1α, and HIF-2α. By modulating these key factors, lactate contributes to the complex mechanisms that govern tumor vascularization and its associated biological consequences. The incorporation of black phosphorus quantum dots (BPQDs) into exosomes effectively inhibits the proliferation, migration, tube formation, and sprouting capabilities of ECs *in vitro*, as evidenced by their disruption of glucose metabolism 182. This disruption is corroborated through comprehensive analyses of metabolites, ATP production, and the c-MYC/Hexokinase 2 signaling pathway. To enhance the targeting specificity towards blood vessels, the exosome membranes were modified with Arg-Gly-Asp (RGD) peptides. Notably, as glucose metabolism accounts for approximately 75% of ATP production in vascular ECs, the inhibition of this pathway results in a profound suppression of angiogenesis, underscoring the therapeutic potential of this nanocomposite approach.

### 6.6 Metabolic Waste Product Removal

Cancer cells produce high levels of lactate and other metabolic waste products as a result of glycolysis. A diverse array of tumors, including lung, gastric, and pancreatic cancers, exhibit heightened lactate content, with intratumorally lactate levels often surpassing those of glucose. This suggests that the direct utilization of lactate, rather than glucose, may confer an advantage by providing a more abundant fuel source for tumor metabolism and potentially enhancing metabolic flexibility 183. Drawing inspiration from the ubiquitous presence of lactate in tumors, a promising strategy to combat cancer emerges by harnessing the intratumorally catalytic consumption of lactate and its subsequent biological effects. By leveraging natural enzymes like lactate oxidase (LOD), lactate can be efficiently eliminated *in vivo*, offering a targeted approach to disrupt tumor metabolism and unleash a cascade of therapeutic benefits. This innovative strategy leverages the tumor's own metabolic signature against it, offering a novel and promising avenue for cancer treatment.

LOD was not enriched at tumor site to exert the therapeutic efficacy. To overcome the above shortcomings, nano particles were used to encapsulate LOD which facilitated to accumulate at tumor site. The produced H_2_O_2_ converted to ROS through Fenton-like reaction for killing tumor cells. Metal organic framework was employed to encapsulate LOD and Fe_3_O_4_ nanoparticles (NPs) for simultaneous delivery 184. LOD carried by MOF depleted lactate to change tumor microenvironment and cell cycles, which committed the tumor cells to apoptosis. Furthermore, Fe_3_O_4_ NPs was degraded to produce Fe^2+^, occurring Fenton-like reaction to kill tumor cells. The tandem nanoreactor with excellent compatibility showed high catalytic efficiency because of high loading of LOD and Fe_3_O_4_, resulting in marked reduced tumor growth. MOF (ZIF-8) was used as carrier to encapsulate Gd-doped CeO_2_ nanorods (Gd/CeO_2_), Syro, LOD 185. Gd/CeO_2_ have demonstrated peroxidase-like activity in weakly acidic conditions, enabling them to catalyze H_2_O_2_ and generate hydroxyl radicals. Lactate depletion could reduce glucose supply as well as mitochondrial damage, thereby reprogramming glycometabolism. This catalytic metabolic reprogramming not only triggers a local response but also stimulates a systemic antitumor immune response. It activates M1-polarized macrophages and CD8^+^ T cells, which are crucial for mounting a robust immune attack against cancer cells. A pH-responsive MOF, coated with mannose-modified PEG for precise targeted delivery to M2-type macrophages, serves as a versatile vehicle, transporting LOD and CRISPR-Cas9-mediated SIRPα genome-editing plasmids 186. Upon encountering the acidic byproduct pyruvate, the MOF (ZIF-67) undergoes controlled decomposition, precisely releasing the plasmids for targeted disruption of the SIRPα gene. undergoes controlled dissociation, releasing the plasmids for targeted SIRPα gene knockout. This genetic modification eliminates the "don't eat me" signal, empowering macrophages with enhanced phagocytic capabilities. In parallel, the lactate-depleting mechanism of ZIF-67@LOx generates cytotoxic H_2_O_2_ and ROS, orchestrating a phenotypic transformation of TAMs from their immunosuppressive M2 state to the immunostimulatory M1 state. The harmonious integration of SIRPα knockout and lactate depletion profoundly reshapes the TME, enhancing macrophage-mediated cancer immunotherapy and fostering a more conducive immune landscape for effective tumor eradication.

LOD serving as a local energy source boost the production of H_2_O_2_. H_2_O_2_ then interacts with a chemiluminescence reagent (CPPO), releasing energy to activate a photosensitizer and generate cytotoxic singlet oxygen, precisely targeting cancer cells. To bypass the formidable barrier posed by the BBB, the nanosystem employs a biomimetic design coated with tumor cell membranes, enabling it to penetrate the BBB and actively seek out glioblastoma multiforme (GBM) cells 187. Upon reaching the GBM cells, the nanosystem releases the therapeutic agents necessary for a combined lactate metabolic therapy and a novel form of chemiexcited PDT that does not rely on direct light excitation. This approach transcends the limitations of traditional PDT in terms of laser penetration depth within the brain, offering a potent and effective treatment option against both laboratory-derived and patient-specific GBM models. The synergy between modulating lactate metabolism and harnessing chemiexcited PDT holds great promise for advancing the treatment of GBM and other central nervous system malignancies.

Fang and coworkers have developed a nanoenzyme, Mn-CuS@BSA-FA, which plays a pivotal role in the transformation of lactate to pyruvic acid, thereby targeting and reducing lactate levels within TME 188. This conversion is facilitated by the interaction of pyrrole-2-ketone derivatives, which are LDHA inhibitors, with Mn^2+^ ions on the surface of CuS nanoparticles. The anticancer efficacy of these inhibitors is amplified upon nanoformulation due to their selective accumulation and the photothermal effects of the nanocomposites. To enhance tumor targeting, Mn-CuS is integrated with polyethylene glycol-folic acid modified bovine serum albumin (BSA) to create Mn-CuS@BSA-FA nanocomposites through hydrophobic and electronic interactions. The catalytic conversion of lactic acid to pyruvate by Mn-CuS@BSA-FA is facilitated by the energy transfer from CuS absorption to the Mn(II) center via the Mn-O bond. Under LED lamp illumination (808 nm), the rate of pyruvate production is significantly increased due to the photothermal effect of CuS nanoparticles in the presence of lactate in cancer cells. Since pyruvate can be converted back to lactate by LDHA, the nanoparticles effectively inhibit this reverse reaction. By engaging with glycolysis, the nanoparticles significantly reduce the cellular ATP levels when present at a concentration of 1 μg ml^-1^ under near-infrared (NIR) irradiation. Additionally, Mn-CuS@BSA-FA exhibits inhibitory effects on the expression of HIF-1α, a key regulator of glycolysis. Consequently, Mn-CuS@BSA-FA emerges as a NIR-responsive glycolytic inhibitor, with potential applications in cancer therapy, due to its ability to suppress cellular ATP levels, LDHA activity, and HIF-1α expression.

**Figure 7:**
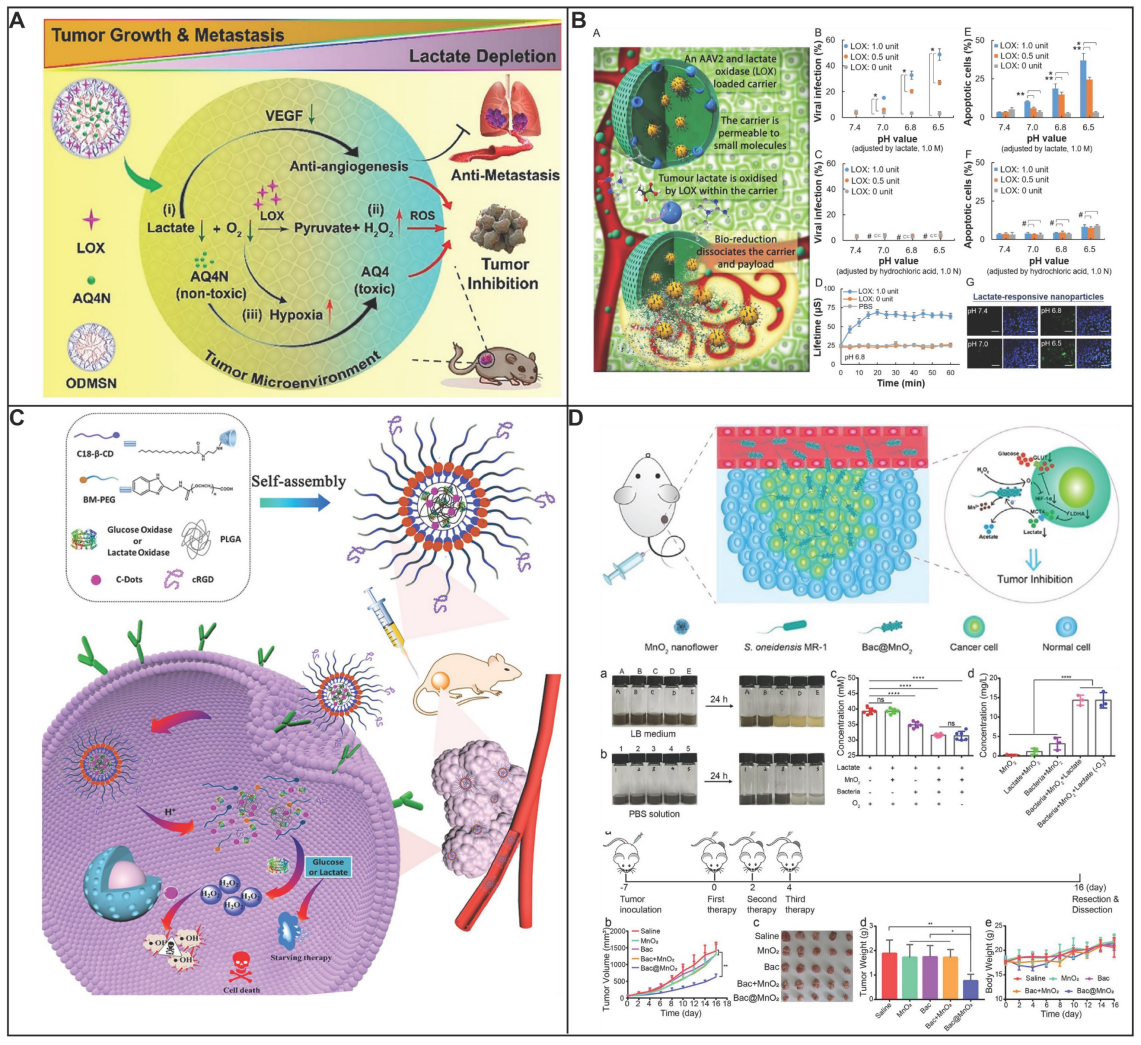
(A) Schematic diagram showing lactate-depletion-enabled tumor microenvironment adjustment and combinational cancer treatment strategy. Reproduced with permission from ref 189. Copyright 2020 Wiley-VCH. (B) Schematic diagram showing tumor bioreduction activated nanoparticle for targeted magnetized virus delivery. Reproduced with permission from ref 190. Copyright 2018 American Chemical Society. (C) Schematic diagram of tumor-targeted micelles as the combination of starving and catalytic therapy based on the generation of highly toxic •OH. Reproduced with permission from ref 191. Copyright 2020 American Chemical Society. (D) Schematic diagram showing tumor targeting of Bac@MnO_2_ and its tumor inhibition mechanism. Reproduced with permission from ref 192. Copyright 2020 Wiley-VCH.

O_2_ was consumed by LOD to catalyze lactate into pyruvate, causing hypoxia in the tumor. Lactate concentrations within tumors exhibit variability, ranging from 10 to 40 mM. Consequently, an elevated dosage of LOD is essential to achieve effective lactate depletion. However, the direct administration of LOD in high concentrations carries the risk of generating H_2_O_2_, which can lead to significant toxic side effects. A delivery system capable of bearing a high load and controlling the sustained release of LOD holds the potential to pioneer an effective enzyme-lactic acid consumption approach for cancer treatment. This system could address the challenge of achieving adequate lactate depletion within tumors. Yu group has successfully utilized dendritic mesoporous silica nanoparticles (DMSN), which are characterized by their expansive radial pore structures and readily available surface areas, for the delivery of LOD (Figure 7A) 189. These DMSNs demonstrate an impressive loading capacity, reaching up to 731.8 μg/ml for LOD, and are capable of providing a sustained release of the enzyme over a period of 72 hours. The use of DMSN for the delivery of LOD has facilitated targeted intra-tumoral distribution of the enzyme, resulting in an astonishing 99.9% reduction in lactate levels within the TME. The substantial decrease in lactate levels was a result of several interconnected effects: the downregulation of Vascular Endothelial Growth Factor, which led to the promotion of anti-angiogenic and anti-metastatic processes; the catalytic production of H_2_O_2_, a cytotoxic agent; and the induction of hypoxia, which in turn activated the co-administered prodrug AQ4N, converting it into its active cancer-fighting form, AQ4, thereby enhancing the effectiveness of chemotherapy. Besides, Tseng *et al.* have developed a hypoxia responsive nanocarrier to encapsulate photosensitive protein by employing the self-assemble of hyaluronic acid which was conjugated by 6-(2-nitroimidazole) hexylamine (Figure 7B) 190. In tumor microenvironment, lactate was allowed to enter into carrier to generate pyruvate with simultaneous oxygen consumption. Hydrophobic 2-nitroimidazole was reduced to hydrophilic 2-aminoimidazole under hypoxia condition, the carrier changed from hydrophobic to hydrophilic, leading to carrier disassembly, thus releasing LOD in TME, enhancing penetration in tumor tissue. The released LOD further exacerbated hypoxia, more adeno-associated virus serotype 2 (AAV2) was released, thus minimizing side effect as well as ectopic transduction of AAV2. The AAV encoded a photosensitive protein produced ROS under irradiation, significantly inhibiting tumor growth. As compared to control group, the tumor was approximately reduced 2.44-fold after two weeks of treatment. The introduction of LOD into nanomedicine could achieve hypoxia responsively precise release of cargo. Besides, magnetic resonance imaging tracking of the virus *in vivo* was achieved by modification of iron oxide nanoparticles to AAV2.

The uniqueness of the tumor microenvironment, including its acidity and sensitivity to ROS, as well as the low content of antioxidants in tumors, leads to a high redox potential and increased sensitivity of tumor cells to ROS. Therefore, the generation of ROS can be utilized to kill tumor cells and inhibit tumor progression. Nanoenzymes with catalase-like properties exhibit better stability than natural enzymes. Interestingly, their pH-dependent manner allows H_2_O_2_ to decompose into H_2_O and O_2_ under neutral conditions, while in acidic conditions, it transforms into hydroxyl radicals. GOx can break down glucose in tumor cells, inhibiting tumor growth through starvation therapy. However, this strategy is not applicable to tumors lacking glucose. Tumors commonly contain large amounts of lactate, and lactate oxidase can similarly produce H_2_O_2_. Utilizing both glucose oxidase and lactate oxidase to generate H_2_O_2_, and subsequently using nanoenzymes to produce ROS for therapy, is more universally applicable. To deliver the aforementioned three enzymes to the tumor, Wang and colleagues used tumor-targeting and pH-sensitive supramolecular micelles (Figure 7C) 191. Through host-guest recognition and self-assembly, they encapsulated GOx and LOD to consume glucose and lactate within the tumor. The glucose and lactate within the tumor are converted into H_2_O_2_, which is then sequentially reduced to highly toxic hydroxyl radicals (OH) by the loaded C-dot nanoenzyme's peroxidase-like activity. A cascade catalytic reaction was observed for its tumor-killing effect. After intravenous injection, this nanocomposite material demonstrated good tumor targeting ability and excellent biocompatibility, indicating its effective anti-tumor action. The nanocomposite effectively combines starvation and catalytic therapy, exerting synergistic anti-cancer effects with minimal side effects and no external addition.

Besides LOD, certain unique bacteria, like the anaerobic bacterium Shewanella oneidensis MR-1 (S. oneidensis MR-1), have the ability to use lactate as an energy source in place of glucose to generate acetate in the presence of active materials such as Fe^3+^ or Mn^4+^ when oxygen is lacking. Due to unique feature of MR-1, Zhang group have used MnO_2_ as electrons acceptors for lactate metabolism (Figure 7D) 192. Nanoscale MnO_2_ nanoflower possessed large surface, facilitating to capture lactate further enhancing lactate consumption. Moreover, MnO_2_ could react with H_2_O_2_ within tumor microenvironment to generate O_2_. Interestingly, the oxygen that is generated can additionally mitigate the production of lactate by suppressing the expression of HIF-1α. This suppression leads to a decrease in the levels of enzymes related to glycolysis, such as GLUT1 and LDHA. Nanoscale MnO_2_ modified MR-1 enabled depletion lactate content in extracellular matrix and inhibition lactate production in cancer cells. After intravenous injection, mice that received the Bac@MnO2 intervention for three times demonstrated a marked reduction in tumor growth over a 16-day period as compared to other groups. The Bac@MnO_2_ treatment group exhibited a notably lower lactate concentration compared to the other control groups. This finding indicates that the significant suppression of tumor growth in the Bac@MnO_2_ group can be attributed to the reduction in lactate levels at the tumor site. The strategy of regulating lactate metabolism through the synergistic action of bacteria and nanoscale MnO_2_ can significantly inhibit tumor progression.

### 6.7 Targeting OXPHOS inhibition

Cancer cells often rely on OXPHOS for their survival and proliferation, with an exaggerated dependence on OXPHOS being a hallmark of cancer stem cells and a common feature in instances of primary or acquired resistance to chemotherapy and tyrosine kinase inhibitors 193. For example, the PD-1-resistant model exhibited a significantly higher reliance on OXPHOS compared to the PD-1-sensitive model, and exposure to X-ray therapy further enhanced OXPHOS activity 194. CSCs, identified across a spectrum of tumor types, are implicated in the processes of tumor metastasis and resistance to traditional therapeutic approaches 195. The metabolic characteristics of CSCs can vary, exhibiting either a predominantly glycolytic or OXPHOSphenotype, influenced by genetic and microenvironmental factors 196. In various cancer types, such as breast 197, leukemia 198, lung, pancreatic 199 and glioblastoma 200, there is a growing body of evidence indicating that CSCs, which are quiescent or slow-cycling and capable of initiating tumor growth, are less reliant on glycolysis. These cells consume less glucose, produce less lactate, yet maintain higher ATP levels compared to their more differentiated cancer cell counterparts. PGC1α, a pivotal regulator of mitochondrial biogenesis known as peroxisome proliferator-activated receptor gamma coactivator 1-alpha, has been identified as overexpressed in CSCs with a heightened OXPHOS phenotype. This overexpression is associated with the promotion of cancer cell migration, the enhancement of ECM proteases (EMP), and the facilitation of immune evasion 201, 202. The reliance on OXPHOS by CSCs is thought to bolster their resistance to nutrient scarcity and metabolic stress, which are common features of the tumor microenvironment in many solid tumors. This adaptation may underlie the resilience of CSCs to conventional treatments and their role in tumor recurrence and metastasis.

**Figure 8:**
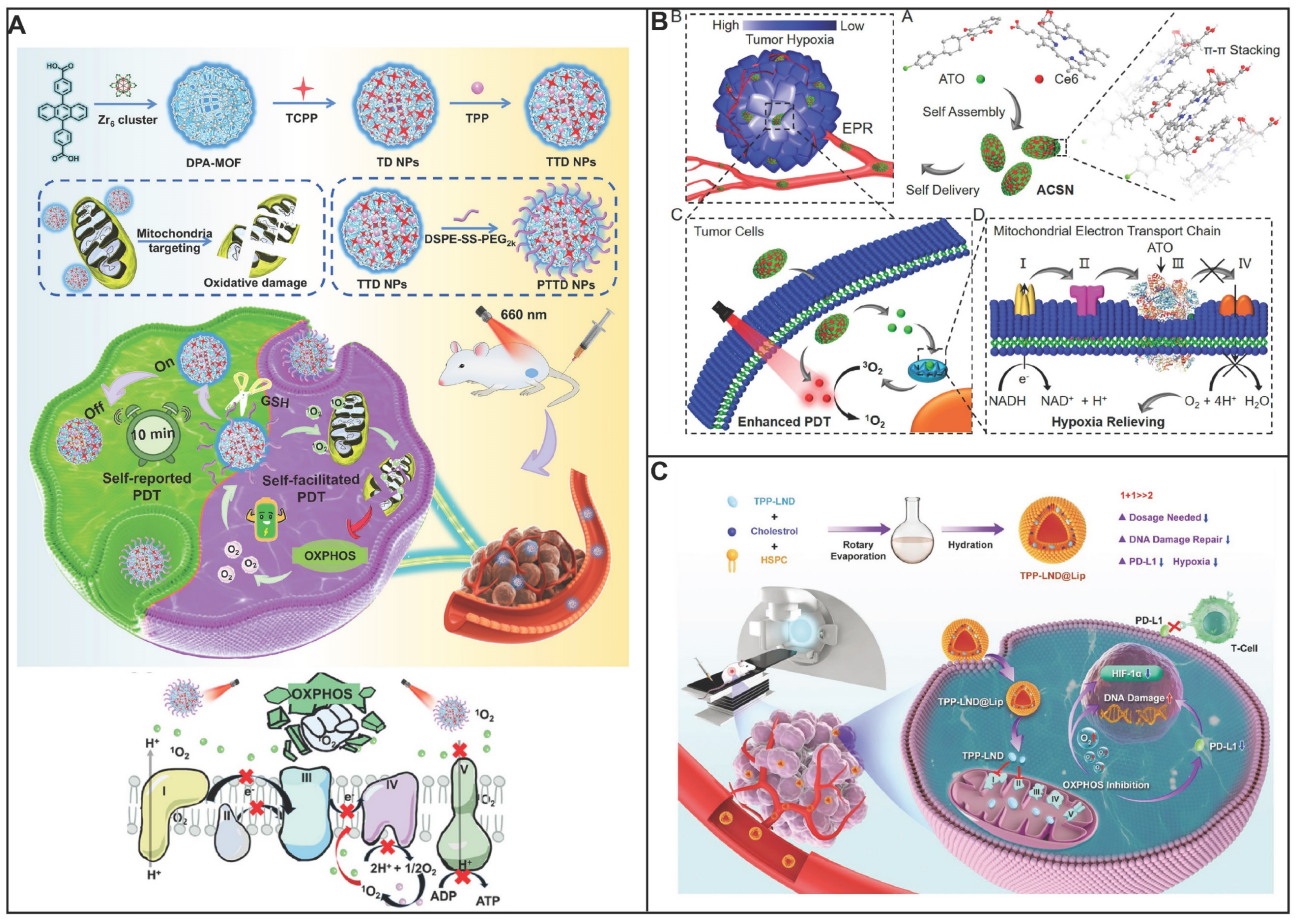
Targeting OXPHOS inhibition. (A) Schematic diagram showing the self-reported and self-facilitated theranostic oxygen nano-economizer for precise and hypoxia alleviation potentiated photodynamic tumor eradication. Reproduced with permission from ref 203. Copyright 2023 Royal Society of Chemistry. (B) Schematic diagram showing the self-delivery nanomedicine for mitochondrial respiratory inhibition enhanced photodynamic tumor therapy. Reproduced with permission from ref 204. Copyright 2020 American Chemical Society. (C) Schematic diagram showing preparation process of TPP-LND@Lip nanoparticles and the metabolic intervention mechanism of sensitized radioimmunotherapy by inhibiting PD-L1 expression and reversing tumor hypoxia. Reproduced with permission from ref 205. Copyright 2023 Wiley-VCH.

The OXPHOS metabolic pathway is inherently dependent on a steady supply of oxygen to catalyze the production of ATP, essential for sustaining cellular life. The persistently high respiration rate observed in tumors, driven by mitochondrial activity, can result in the depletion of oxygen, creating a hypoxic environment. As essential organelles for cellular respiration, mitochondria might exacerbate tumor hypoxia, thereby limiting the effectiveness of ROS-dependent cancer therapies, including sonodynamic therapy (SDT), PDT, and radiotherapy. By targeting the OXPHOS pathway within the mitochondria of cancer cells to reduce oxygen consumption, the concentration of ROS can be enhanced, potentially improving therapeutic outcomes. However, the indiscriminate distribution, swift clearance from the bloodstream, and the challenges of solubility and bioavailability associated with OXPHOS inhibitors and photosensitizers can impede the enhancement of tumor oxygenation and the effectiveness of PDT. Furthermore, the precise colocalization of OXPHOS inhibitors and PSs is imperative for an oxygen-conserving PDT approach, ensuring a uniform distribution of both agents to prevent the uneven allocation of PSs and oxygen. Li and colleagues have developed a mitochondrial-targeted PDT approach that disrupts theOXPHOS pathway in tumor cell mitochondria, effectively alleviating the hypoxic state within tumor tissues (Figure 8A) 203. This intervention not only relieves hypoxia but also harnesses the freed oxygen to generate additional singlet oxygen through tetrakis(4-carboxyphenyl) porphyrin, enhancing the self-facilitating PDT effect. The PTTD nanoparticles (NPs) demonstrated a significant reduction in the activity of complex II, with a decrease 4.5 times greater than that of the PTD NPs alone. The PTTD NPs' superior mitochondrial targeting and uptake resulted in a substantial inhibition of the mitochondrial complexes' activity. Under optimal laser exposure, these NPs produced a substantial amount of singlet oxygen, which targeted the entire OXPHOS pathway, reducing oxygen consumption and mitigating tumor hypoxia. In comparison to other treatments, the PTTD NPs combined with laser therapy maintained 85% of the initial relative oxygen content, surpassing other groups by 30%. By generating singlet oxygen, the PTTD NPs destroyed the OXPHOS pathway, conserving oxygen and enhancing the PDT effect. This meticulously designed oxygen nano-economizer integrates diagnostic and therapeutic advantages, optimizing laser exposure duration and self-reporting capabilities to alleviate hypoxia, enabling self-facilitating PDT. The PTTD NPs, with their tumor-specific accumulation and defined synergistic therapeutic mechanism, offer a high level of safety and have shown to be highly effective in antitumor treatments both *in vitro* and *in vivo*. As anticipated, these PTTD NPs significantly alleviated tumor hypoxia and inhibited tumor growth, demonstrating their potential as a promising therapeutic strategy. Researchers have explored the use of atovaquone (ATO) as an inhibitor of OXPHOS to facilitate an oxygen-conserving PDT for hypoxic tumors (Figure 8B) 204. ATO, when combined with chlorin e6 (Ce6) at an optimized ratio, can self-assemble into well-defined nanosized aggregates known as ACSN. These aggregates are formed through π-π stacking and hydrophobic interactions, boasting a high drug loading capacity and excellent long-term stability. The nanodelivery system, being a carrier-free system, can selectively concentrate in tumor tissues via the EPR effect and effectively infiltrate hypoxic regions of tumors. Upon internalization by tumor cells, ATO disrupts the ETC, slowing the rate of oxygen consumption and alleviating the hypoxic microenvironment. This intervention is pivotal for boosting the production of ROS and enhancing the efficacy of PDT. As a result, ACSN has demonstrated potent antitumor activity with minimal systemic toxicity. This self-delivery nanomedicine approach for O_2_-economized PDT presents a promising alternative strategy, potentially accelerating the advancement of carrier-free nanomedicines for tumor theranostics, particularly in challenging microenvironments where traditional therapies may falter. Radiotherapy (RT) faces challenges in clinical effectiveness due to the reduced DNA damage under hypoxic conditions and the development of immune tolerance resulting from increased expression of PD-L1 (Figure 8C)^205^. Suppressing intracellular PD-L1 expression has been demonstrated to enhance the sensitivity of RT by impeding DNA repair mechanisms. Lonidamine (LND), which inhibits mitochondrial complexes I and II, is used to decrease cellular oxygen consumption, thereby sensitizing cells to RT. However, LND's high hydrophobicity results in slow diffusion in the cytoplasm, limiting its delivery to tumor tissues and particularly to the mitochondria of cancer cells. Consequently, the development of mitochondria-targeted LND formulations is crucial to enhance its efficacy. To address the limitations of free LND, triphenylphosphine cations (TPP) were conjugated to LND to confer mitochondrial targeting, significantly reducing the required dose for effective OXPHOS inhibition from 300 µM to just 2 µM. Furthermore, TPP-LND is encapsulated within liposomes to create TPP-LND@Lip nanoparticles. This encapsulation strategy not only enhances the delivery of TPP-LND to the tumor site but also reverses the hypoxic tumor microenvironment, leading to increased DNA damage and a reduction in PD-L1 expression through the activation of AMPK. These meticulously designed TPP-LND@Lip nanoparticles have shown to be more potent than traditional anti-PD-L1 antibodies in sensitizing RT, offering a promising approach to overcome the barriers associated with hypoxia and immune evasion in cancer treatment.

### 6.8 Combination therapy by inhibiting glycolysis and OXPHOS

Cancer cells are distinguished by their exceptional ability to generate ATP through glycolysis, a process that can be up to 50 times more efficient in these cells compared to their normal counterparts. Furthermore, these cells also excel in OXPHOS, utilizing the mitochondrial ETC to produce ATP at a high rate. The metabolic flexibility of tumor cells, driven by heterogeneity and compensatory mechanisms, enables them to switch between glycolysis and OXPHOS to meet their energy requirements. In addition, not all cancer cells such as prostate cancer 206, Myc driven triple negative breast cancer 207, pancreatic cancer 208, cancer stem cells 15, shifted glucose metabolism from OXPHOS to glycolysis. Harnessing this insight, a more potent therapeutic strategy could be to simultaneously disrupt both metabolic pathways. By doing so, the strategy aims to thwart the cancer cells' ability to pivot to an alternate energy source when one pathway is compromised, thereby effectively cutting off their energy supply. This can be achieved by combining a glycolysis inhibitor with a compound that impairs mitochondrial function, potentially leading to a more effective reduction in the energy available to cancer cells. This dual-targeting approach not only leverages the metabolic vulnerabilities of cancer cells but also addresses their adaptive nature, making it a compelling strategy for enhancing the efficacy of antitumor bioenergetic therapies.

**Figure 9:**
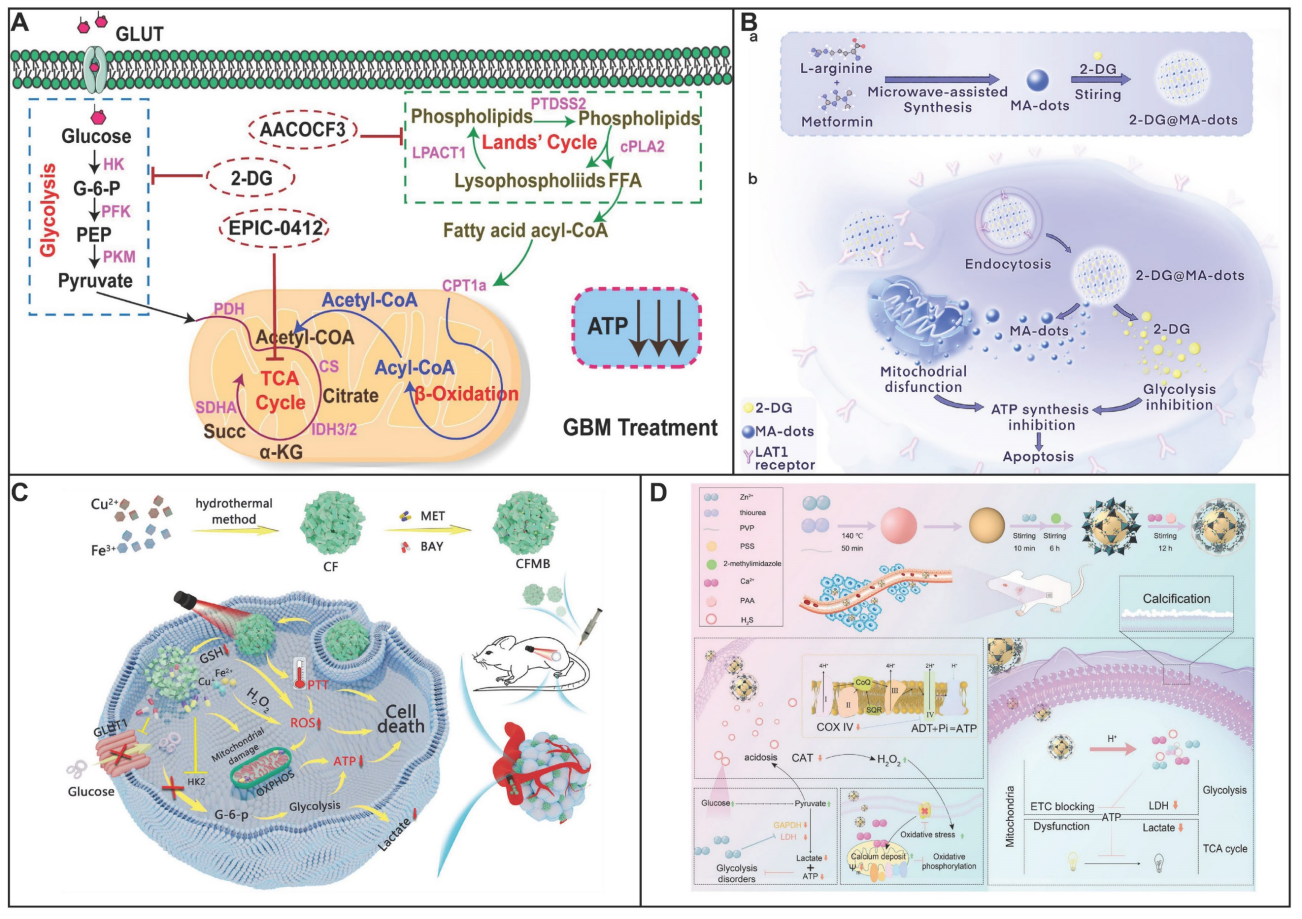
Combination therapy by inhibiting glycolysis and OXPHOS. (A) Schematic diagram showing the mechanistic scheme by which the TCA-phospholipid-glycolysis metabolism pathway was blocked using specific inhibitors to suppress the tumor proliferation in GBM. Reproduced with permission from ref 209. Copyright 2022 Ivyspring. (B) Schematic diagram showing the synthesis of 2-DG@MA-dots and its application in tumor cells. Reproduced with permission from ref 210. Copyright 2024 Elsevier. (C) Schematic diagram showing the synthesis of CFMB and its therapeutic mechanism for boosting energy deprivation by synchronous interventions of glycolysis and OXPHOS for bioenergetic therapy synergetic with CDT/PTT. Reproduced with permission from ref 211. Copyright 2024 Wiley-VCH. (D) Schematic diagram showing fabrication and energy disorders-therapeutic schematics of ZSZC NPs. Reproduced with permission from ref 212. Copyright 2024 Wiley-VCH.

GBM is characterized by its reliance on distinct energy pathways to fulfill the substantial metabolic needs of its cancer cells, with ATP at the heart of cellular metabolism and signaling. TCA is often replenished by metabolic intermediates derived from pathways like FAO and aerobic glycolysis. In GBM, the FAO pathway is particularly upregulated to meet the high ATP production and anabolic demands of the tumor cells. Moreover, mitochondrial ATP plays a pivotal role in fatty acid uptake and transport in endothelial cells, which are integral to tumor angiogenesis. Elevated ATP levels within brain tumors are associated with accelerated growth rates and diminished immune responses. Targeting the principal ATP production pathways including glycolysis, FAO, and OXPHOS presents a promising therapeutic strategy. Yang *et al.* has revealed that ATP production from FAO and the TCA cycle in GBM accounts for 45% and 47% of mitochondrial respiration, respectively, with glycolysis contributing 45% to the overall ATP content 209. A targeted triple therapy that disrupts the TCA-phospholipid-glycolysis axis was developed to effectively curtail ATP synthesis and tumor progression (Figure 9A). The therapy comprises SMI EPIC-0412, which impedes the TCA cycle by disrupting the interaction between the long non-coding RNA HOTAIR and the enhancer of zeste homolog 2; AACOCF3, which partially inhibits FAO by modulating phospholipid metabolism; and 2-DG, a HK2inhibitor, which targets the glycolytic pathway. This synergistic approach has been shown to reduce ATP production within mitochondria and through glycolysis to a mere 5% of its original capacity, leading to cell cycle arrest in the G0/G1 phase and a significant suppression of tumor growth. The enhanced survival outcomes observed in tumor-bearing mice underscore the potency of this therapeutic strategy. However, the effectiveness of these drugs can be attenuated because they are differentially metabolized, preventing them from reaching the target tissues or cells. To mitigate this, co-encapsulation within nanoparticles ensures simultaneous delivery to the target site, thereby maximizing therapeutic efficacy.

The application of high concentrations of OXPHOS and glycolysis inhibitors including metformin or 2-DG poses a risk to healthy tissues, potentially inducing severe side effects such as hypoglycemia, cardiac toxicity, and lactic acidosis. To address this challenge and minimize inhibitor dosages, Chen *et al.* have ingeniously crafted a nanoformulation to encapsulate these agents (Figure 9B) 210. The process begins with the synthesis of carbon dots from metformin and L-arginine through a microwave-assisted method. Then, 2-DG is seamlessly integrated into these carbon dots via a physical blending technique, yielding a novel nanocomplex known as MA-dots. Due to the presence of L-arginine in MA-dots, these MA-dots are selectively bind to the large neutral amino acid transporter 1, which is frequently overexpressed on the surface of tumor cells, ensuring precise delivery to the tumor site. This innovative approach cleverly overcomes the difficulty of directly co-assembling 2-DG and metformin due to their lack of inherent interaction. The 2-DG@MA-dots enable the co-delivery and synchronized release of both agents within the tumor cells, amplifying the therapeutic impact. In the acidic tumor microenvironment, the nanocomplex rapidly disintegrates, liberating 2-DG to inhibit glycolysis via HK2, while metformin targets mitochondrial OXPHOS. This dual-targeted release mechanism is highly responsive to the tumor's acidic conditions (pH 5.5), with an impressive 40% release of 2-DG within the first hour, significantly accelerating the drug release rate. In contrast, under neutral conditions (pH 7.4), the 2-DG@MA-dots maintain their particle size and morphology, ensuring that the therapeutic action is concentrated at the tumor site. This dual-action strategy significantly diminishes ATP production in tumor cells, leading to apoptosis. *In vitro* studies using human lung tumor cells have shown that the half-maximal inhibitory concentration (IC_50_) of 2-DG@MA-dots is markedly lower than that of metformin or 2-DG alone, achieving a nearly 100-fold and 30-fold reduction in IC_50_ values to 11.78 μg/mL, down from 1159 μg/mL and 351.20 μg/mL, respectively. *In vivo* studies with A549 tumor-bearing mice corroborate the superior efficacy of this nanoformulation. While a combination of low-dose 2-DG and metformin showed no significant tumor growth inhibition, the 2-DG@MA-dots treatment group demonstrated a substantial decrease in tumor volume. The mean final tumor volume in the combination treatment group was approximately 89 times greater than that in the 2-DG@MA-dot group, underscoring the enhanced potency of the nanoformulation.

The inherent complexity and heterogeneity of tumors suggest that energy deprivation alone may not suffice to completely eradicate them. Hydroxyl produced by CDT not only impede OXPHOS process but also exert a direct cytotoxic effect on cancer cells. Furthermore, PTT can augment the catalytic efficiency of CDT, thereby enhancing its therapeutic impact. Wei *et al.* have proposed a multifaceted therapeutic strategy that integrates simultaneous interventions in both glycolysis and OXPHOS, complemented by the introduction of CDT and PTT (Figure 9C). This approach aims to starve the tumor of energy while significantly increasing the potential for complete tumor eradication. To realize this strategy, they have developed a sophisticated nanoplatform, CuFe_2_O_4_@MET@BAY (CFMB), which encapsulates metformin and BAY-876 within a CuFe_2_O (CF) matrix 211. This innovative CFMB platform is designed to selectively deliver its therapeutic agents to the tumor microenvironment, where it is activated by the tumor's elevated levels of GSH to release metformin and BAY-876. Metformin simultaneously suppresses glycolysis and OXPHOS by inhibiting HK2 and impairing mitochondrial function, effectively depriving the tumor of vital energy sources. BAY-876 further amplifies this effect by inhibiting glucose uptake through the downregulation of GLUT1, leading to a more pronounced depletion of energy reserves. The GSH-triggered degradation of CFMB also liberates Fenton-like/Fenton reagent ions of Cu^+^/Fe^2+^, which catalyze the conversion of H_2_O_2_ into hydroxyl radicals. This not only disrupts OXPHOS but also amplifies the CDT effect through GSH depletion. The photothermal properties of CFMB, when activated by 808 nm light irradiation, enable it to kill cancer cells through PTT and enhance the production of hydroxyl radicals, thereby boosting the CDT effect. *In vitro* studies have demonstrated the superior therapeutic efficacy of CFMB nanoparticles, evidenced by a significant reduction in lactic acid and ATP levels in tumors from mice treated with CFMB. The CFMB platform outperforms both CFM and CFB nanoparticles in tumor suppression, attributed to the synergistic metabolic intervention by metformin and BAY-876, which results in a more profound energy deprivation within the tumor. By harnessing these synergistic effects, CFMB not only enhances bioenergetic therapy but also potentiates CDT and PTT in a mutually reinforcing manner. In the treatment group administered CFMB in conjunction with NIR, there was a marked reduction in both lactic acid and ATP levels. Additionally, the weight of the tumors in mice treated with this combination was notably the lowest among all treatment groups. This novel, bioenergetics-based approach to synergistic cancer treatment represents a significant advancement in the field of oncology, offering a promising new avenue for the design of more effective cancer therapies.

ZnS@ZIF-8@CaP, synthesized by Ding and colleagues, was employed to disrupt glycolysis and OXPHOS to dismantle the energy metabolism of cancer cells (Figure 9D) 212. Within the TME, the decomposition of ZnS yields H_2_S, an efficacious modulator of the ETC. This action not only impedes OXPHOS but also triggers an upsurge in ROS, exacerbating the destabilization of the cellular energy infrastructure. Furthermore, the anomalous aggregation of Zn^2+^ within the mitochondria acts as a metabolic Trojan horse, precipitating mitochondrial dysfunction and bolstering a multifaceted approach to mitochondrial disruption. The CaP component, rich in calcium ions, exacerbates mitochondrial damage by inducing a state of calcium overload, a condition that significantly amplifies the mitochondrial stress induced by H_2_S. This strategy is further fortified by the interference of H_2_S in glycolysis, effectively crippling the cancer cell's ability to generate energy through this pathway. The presence of Ca^2+^ ions also play a crucial role in inhibiting OXPHOS, reinforcing the overall suppressive effect on the cancer cell's metabolism. The emergence of acidification and calcification during the therapeutic process was an unexpected yet serendipitous discovery that has propelled the treatment forward. Chaube and coworkers have demonstrated that combination of metformin and DCA exhibited synthetic lethality in melanoma via inhibit glycolysis and OXPHOS 213.

Cytochrome C oxidase (COX), a copper-containing enzyme, is a key component of mitochondrial complex IV and is essential for OXPHOS. The activity of COX is highly dependent on the availability of copper. Limited copper supply has been shown to disrupt mitochondrial metabolism and hinder cancer cell proliferation, particularly in breast cancer cells that have an increased requirement for copper. However, the effectiveness of copper-chelating drugs in treating primary tumors is often inadequate for many cancer types, as they reduce OXPHOS and, paradoxically, enhance glycolysis. To address these challenges, advanced therapeutic nanomaterials have been engineered to enhance cellular uptake, enable controlled and targeted drug release, and maintain a sustained therapeutic effect, which could potentially enhance antitumor outcomes. However, solely inhibiting mitochondrial function may foster drug resistance in cancer cells, as they can adapt by switching their energy supply from OXPHOS to glycolysis. Therefore, there is a pressing need for a comprehensive therapeutic agent that can simultaneously inhibit both glycolysis and respiration to counteract the metabolic reprogramming of cancer cells, thereby improving the efficacy of cancer treatments. Lei *et al.* has crafted an innovative metabolic inhibitor in the form of Zn-carnosine metallodrug network nanorods (Zn-Car MNs), designed to concurrently suppress OXPHOS and glycolysis 214. Carnosine (β-Ala-L-His), a natural dipeptide was chosen for its ability to inhibit glycolysis by clearing glycolytic intermediates, to serve as the organic ligand. The Zn-Car MNs was synthesized using a straightforward "one-pot" approach that involved the coordination of carnosine with zinc ions, resulting in a high carnosine loading efficiency of 61 wt%.

The positive surface charge of the nanorods facilitated their accumulation within mitochondria. The Zn-Car MNs operates on a principle of "ion exchange" with zinc, which leads to a significant depletion of mitochondrial copper, accompanied by the copper-triggered release of both zinc and carnosine within cancer cells. Crucially, when cancer cells were exposed to Zn-Car MNs, there was a substantial reduction in both mitochondrial OXPHOS activity and glycolysis, resulting in a marked decrease in ATP levels. This was complemented by a weakened mitochondrial membrane potential and an increase in oxidative stress. The Zn-Car MNs demonstrated a robust therapeutic effect on breast cancer models (which are sensitive to copper depletion) and colon cancer models (which are less sensitive to copper depletion), outperforming the conventional copper chelator in terms of treatment efficacy.

To enhance the efficacy and safety of combination therapies, theCI serves as an invaluable quantitative tool for assessing the degree of therapeutic synergy or antagonism among drugs. This unified metric streamlines the process of pinpointing drug combinations that can achieve the most significant antitumor effects while remaining within the boundaries of clinical tolerability. By doing so, the CI effectively expands the therapeutic window, thus increasing the likelihood of successful outcomes in combination therapies, particularly in the realm of cancer treatment. The significance of using CI in nanomedicine is highlighted by its ability to account for the unique properties of nanoscale drug delivery systems, such as enhanced EPR effect, which can influence drug distribution and interaction at the tumor site. The application of the CI in nanomedicine is essential for the systematic exploration of drug interactions within nanoscale formulations, facilitating the advancement of personalized and precise cancer treatments.

## 7. Challenges

The achievements of nanomedicine in modulating cancer glucose metabolism are encouraging, yet challenges remain for its practical clinical use. Nanomedicines present unique biosafety challenges beyond those of traditional pharmaceuticals, primarily due to the nanocarrier's impact on biodistribution and potential for tissue overexposure and toxicity. The immunogenic potential of nanocarriers also poses a risk of hypersensitivity reactions. Comprehensive preclinical assessments of nanocarrier-tissue interactions are vital to preempt and mitigate clinical safety concerns. To address these concerns, the biodegradability of therapeutic nanomaterials must be a design priority, featuring chemical bonds or noncovalent interactions that respond to physiological triggers. The emergence of biodegradable nanomaterials is a significant advancement, ensuring the therapeutic agents are safely eliminated post-treatment, thus minimizing adverse effects.

Cancer's metabolic heterogeneity, often relying on glutamine pathways and exhibiting plasticity that can undermine treatment efficacy, necessitates the development of nanotherapeutics that can co-regulate multiple metabolic pathways. Such an integrated approach not only enhances the effectiveness of current treatments but also opens new avenues for therapies targeting the metabolic vulnerabilities of various tumors. With the progression of nanotechnology and a more profound understanding of the tumor microenvironment, it is anticipated that an increasing number of nanomedicines will be adopted in clinical settings, providing patients with more potent and secure treatment options.

In the research and development R&D phase of nanomedicines, a rational design approach emphasizing simplicity, efficiency, and scalability is crucial. High-throughput screening, AI, and ML expedite formulation optimization. Establishing global regulatory protocols tailored to nanomedicine's unique properties, such as material composition, particle characteristics, and interactions with biological systems, is imperative for industrial and clinical advancement. Refining current frameworks to accommodate these specifics ensures safe and effective nanomedicine translation from lab to patient.

## 8. Clinical Potential of Nanomedicine in Regulating Glucose Metabolism

Regulating glucose metabolism offers a promising strategy for selectively starving cancer cells of energy and nutrients, thereby enhancing therapeutic outcomes. Expanding further on the potential of nanomedicine in revolutionizing cancer therapy, let us delve into the multifaceted advancements and their implications for the future of oncology. Firstly, the precision and specificity of nanomedicine's approach to modulating glucose metabolism offer a level of targeting that was previously unattainable. By harnessing the unique properties of nanoparticles, such as their size, shape, and surface chemistry, carriers were designed that navigate the complex tumor microenvironment with remarkable precision. This enables the delivery of therapeutic agents directly to cancer cells, minimizing off-target effects and maximizing therapeutic efficacy. Moreover, the adaptability of nanocarriers to respond to tumor-specific cues, such as pH gradients, enzyme activity, or redox potential, further enhances their effectiveness. These stimuli-responsive nanocarriers can be programmed to release their payload only in the presence of specific tumor markers, ensuring that drugs are activated precisely where and when they are needed. The synergy between nanotechnology and immunotherapy is particularly exciting. By leveraging nanoparticles to deliver immune-stimulatory agents or cancer antigens directly to immune cells, we can amplify the immune system's response against cancer. This approach not only strengthens the body's natural defenses but also overcomes some of the limitations of traditional immunotherapy, such as limited antigen presentation or immune suppression within the tumor microenvironment. Additionally, the integration of theranostics into cancer care represents a significant step forward in personalized medicine. By combining diagnostic and therapeutic functions within a single platform, theranostics allows for real-time monitoring of treatment response and the ability to adjust treatment strategies in response to individual patient outcomes. This approach has the potential to significantly improve patient outcomes by ensuring that each patient receives the most appropriate and effective treatment tailored to their unique needs. However, the translation of these scientific breakthroughs into clinically viable solutions requires careful consideration of safety, efficacy, and accessibility. Rigorous preclinical and clinical testing is essential to ensure that nanomedicines are safe for use in humans and that they demonstrate meaningful therapeutic benefits. Furthermore, regulatory pathways must be adapted to support the rapid yet responsible integration of nanomedicine into standard cancer care. It is crucial to continue investing in research and innovation to unlock the full potential of nanomedicine in cancer therapy. Collaboration between researchers, clinicians, and industry partners is essential to accelerate the development of new nanomedicines and to ensure their timely and widespread adoption. In conclusion, the future of cancer therapy is bright with the potential of nanomedicine to revolutionize our understanding and treatment of this devastating disease. By harnessing the precision and adaptability of nanotechnology, cancer cells are targeted with unprecedented specificity and effectiveness, while minimizing harm to healthy tissues. As continuing to explore the boundaries of nanomedicine, the promise of improved patient outcomes and a paradigm shift in cancer care becomes increasingly tangible.

## 9. Conclusion

Modulating glucose metabolism presents an auspicious tactic for depriving cancer cells of vital energy and nutrients selectively, thus amplifying the effectiveness of therapeutic interventions. However, this approach is not without its drawbacks, such as limited efficacy and side effects related to the solubility, safety, targeting, and half-life of the therapeutic agents. As nanotechnology continues to evolve, researchers have discovered that it can address these shortcomings. The application of nanotechnology in the treatment and diagnosis of various diseases has led to improvements in the bioavailability of therapeutic agents, the achievement of controlled release of nanomedicines, the protection of unstable therapies, and the extension of the residence time of nanomedicines at the site of the lesion. To further advance cancer treatment with nanomedicine, it is crucial to gain a deeper understanding of the toxicological profiles, long-term stability, safety data, and clearance mechanisms of these novel therapies. Additionally, the development of multifunctional nanoparticles presents new opportunities for creating sophisticated cancer treatments. Integrating glucose metabolism inhibitors and therapeutic agents into a single formulation could lead to a more potent therapeutic effect. In summary, while challenges persist in the development of nanomedicine for cancer therapy through glucose metabolism modulation, the field is replete with significant potential and opportunities. Overcoming these challenges will unlock a future for nanomedicine rich with hope and promise.

## Author Contributions

XW (Xudong Wang), LW developed the review outline, drafted and wrote the manuscript. XW (Xudong Wang), MC and QH developed the figures. XW (Xudong Wang), XW (Xueting Wang), MC and WA revised the manuscript. All authors contributed to the article and approved the submitted version.

## Figures and Tables

**Table 1:** Drug targeting glucose metabolism for cancer therapy.

Target pathway and protein	Agent	Development stage	Refs
**Glut1**	WZB117, BAY-876, and RNAi	Preclinical studies	16, 17
**Glut4**	Ritonavir, GLUT4-IN-2, KL-11743, Fasentin	Preclinical studies	18
**Hexokinases**	2‑deoxyglucose, lonidamine, 3‑bromopyruvic acid, methyl jasmonate	Preclinical and clinical studies	19-22
**Phospho-fructokinase 1**	PFK158	Preclinical studies	23
**Aldolase**	Itaconate, raltegravir (an antiretroviral agent that targets HIV integrase) dipicolinic acid, naphthalene 2,6-bisphosphate, Aldometanib, FBA-IN-1	Preclinical studies	24-27
**Glycerdehyde 3-phosphate dehydrogenase**	DC-5163, Misetionamide	Preclinical studies	28, 29
**Pyruvate kinase isoform M2 (PKM2)**	TLN‑232, RNAi	Preclinical and phase II clinical studies	30, 31
**Lactate dehydrogenase A(LDHA)**	GNE‑140, FX11, galloflavin, and RNAi	Preclinical studies	32
**Pyruvate dehydrogenase kinase**	dichloroacetate	Phase II clinical trials	33
**IDH1, IDH2**	Ivosidenib, AGI-5198, Agios 135, ML309 HCl, Novartis 224, Novartis 556, GSK864, BAY-1436032, Sanofi 1, SYC-435; AGI-6780, Enasidenib; Vorasidenib (AG-881)	Preclinical data and Phase III clinical trials only	34
**Malic enzyme**	diethyl oxaloacetate, NPD387, embonic acid	Preclinical data only	35-37
**Mitochondrial complex I**	Metformin	Approved agent (not for cancer)	38

**Table 2:** Nanoparticle-mediated therapies for cancer treatment through targeting glucose metabolism.

Strategies	Nanoparticles type	Active pharmaceutical ingredients	Tumor model	Refs
Targeting glucose transporter inhibition	hyaluronic acid	phloretin,	breast cancer	74
	gold nanorods	diclofenac, gold nanorods	cervical cancer	75
	gold nanoparticles	hairpins for glut1, glut3	/	76
	cationic lipid assisted PEG-PLA	siRNA	pancreatic cancer	77
	Poly β-amino ester	BAY-876, CTLA-4-antagonizing aptamer	breast cancer	78
	host-guest complexation	pyropheophorbide a, JQ1	pancreatic cancer	79
	MOF	Zn^2+^, DNAzyme	melanoma	80
		BAY-876, V-9302	pancreatic cancer	82
	Escherichia coli Nissle, human serum albumin	Escherichia coli Nissle, BAY-876, paclitaxel		83
	mPEG-co-P(Asp)-g-TEPA-g-DC	miR-519C, gemcitabine	pancreatic cancer	84
Targeting glucose depletion by glucose oxidase	hollow MSN	collagenase, Cu^2+^	pancreatic cancer	85
	erythrocyte membrane cloaked MOF	GOx, tirapazamine	colon cancer	88
	cancer cell membrane camouflaged MOF	GOx, CAT, porphyrin	breast cancer	89
	hydroxyethyl starch	GOx, HBO, DOX, Cu^2+^	breast cancer	90
	hyaluronic acid	GOx, MnO_2_,	colon cancer	91
	P(PBEM-co-PEM)	GOX, quinone methide	/	92
	dendritic MSN	GOx, Fe_3_O_4_	breast cancer	93
	MOF	GOx, AQ4N	liver cancer	97
	MnO_2_/hyaluronic acid	GOx, MnO_2_	colon Cancer	91
	tumor cell membrane-camouflaged mesoporous Fe_3_O_4_	GOx, Fe_3_O_4_, PFP	osteosarcoma	94
	amphiphilic peptide-modified GalNAc	GOx. lysosome-targeting chimeras	liver cancer	95
	MOF	GOx, 1-methyltryptophan	melanoma	96
	tumor cell membrane, MOF	GOx, AQ4N	liver cancer	97
	SiO_2_	GOx, Celecoxib, SN-38	breast cancer	98
Modulation of glycolytic enzymes	liposome	3-bromopyruvate	pancreatic cancer	100
	gold nanoparticles	3-bromopyruvate	/	101
	Silver nanoparticles	Juglone silver nano framework	/	103
	peptide	SK(TPA-1)LVFF	prostatic/breast cancer	104
	albumin/lactoferrin	disulfiram, shikonin	glioma	105
	polypeptide/ hyaluronic acid	doxorubicin, PKM2 siRNA	lung cancer	106
	polypeptide	ICG, PKM2 siRNA	breast cancer	107
	DNA- FeS_2_	curcumin, FeS_2_	/	108
Targeting lactate metabolism regulation	CLAN	siRNA, anti-PD-1 antibody	breast cancer	110
	HA- metalphenolic nanomedicines	GSK2837808A, IR780, Ca^2+^	breast cancer	111
	WS_2_	FX11, WS_2_	breast cancer	112
	APEG-PAsp(PEI)	LDHA siRNA, oxaliplatin	colorectal cancer	113
	R-mPDV/PDV	LDHA siRNA, doxorubicin	breast cancer	114
	Silica nanoparticles	doxorubicin, silybin, LDHA-imprinted biodegradable silica nanoparticles	breast cancer	115
	hollow MSN	siRNA, hydroxycamptothecin	melanoma	117
	ROS-sensitive polymer	syrosingopine, cisplatin	osteosarcoma	118
	CuSe/CoSe_2_/BSA	CuSe/CoSe_2_, syrosingopine	breast cancer	119
	MOF	syrosingopine, Mn^2+^ CRISPR	colorectal cancer	120
	Fe_3_O_4_	LOD, syrosingopine	melanoma	121
	CoP/NiCoP	fluvastatin sodium, CoP/NiCoP	breast cancer	122
	ultra-pH sensitive micelle nanoparticles	AZD3965, anti-PD-1	lung cancer	123
	DNA	Ca^2+^, MCT4 siRNA	breast cancer	124
	polyamidoamine	MCT4 siRNA, diethyl maleate, ferrocene	breast cancer	125
	MOF	LOD, MCT4 siRNA, Fe^3+^	melanoma	126
Reprogramming metabolic pathways	CDs doped g-C_3_N_4_	arginine aptamer, carbon dots, graphitic carbon nitride	breast cancer	128
	PLGA	syrosingopine, Tppa	breast cancer	131
	polymer micelle	dichloroacetate, anti-PD-L1 antibody	osteosarcoma	134
	PLGA-PEG	platin-M, dichloroacetate	breast cancer	139
	Ångstrom-scale silver particles	Ångstrom-scale silver particles	osteosarcoma	140
	Polymer (P1)	JX06, metformin	cancer patients with diabetes	141
	emulsion	KIRA6, α-tocopherol,	breast cancer	161
	exosomes	BPQDs	/	182
Metabolic waste product removal	MOF	LOD, Fe_3_O_4_	breast cancer	184
	ZIF-8	LOD, syrosingopine, CeO_2_	hepatocellular carcinoma	185
	MOF	LOD, CRISPR	breast cancer	186
	hemoglobin	LOD, chlorin e6	glioblastoma	187
	BSA	pyrrole-2-ketone derivatives, CuS NPs	/	188
	ODMSNs	LOD, AQ4N	breast cancer	189
	hyaluronic acid	LOD, AAV2	non-small cell lung cancer	190
	micelles	GOx, LOD, carbon dots	melanoma	191
	Shewanella oneidensis MR-1	Shewanella oneidensis MR-1, Mn^4+^	Colon Cancer	192
Targeting OXPHOS inhibition	MOF	TCPP	breast cancer	203
	self assembly	chlorin e6, atovaquone	breast cancer	204
	liposomes	lonidamine, radiotherapy	lung cancer	205
Combination therapy by inhibiting glycolysis and OXPHOS	/	SMI EPIC-0412, 2-DG, AACOCF3	glioblastoma	209
	CDs	2-DG, CDs	lung cancer	210
	CuFe_2_O_4_	metformin, BAY-876, Cu^+^/Fe^2+^	breast cancer	211
	ZIF-8	ZnS, Ca^2+^	breast cancer	212
	metallodrug network nanorods	carnosine, Zn^2+^	breast cancer	214

Note: CLAN, cationic lipid-assisted nanoparticles; GOx, glucose oxidase; CAT, catalase; HBO, hyperbaric oxygen; PFP, perfluoropentane; GalNAc, N-acetylgalactosamine; CDs, carbon dots; MOF, metal organic framework; g-C_3_N_4_, graphitic carbon nitride; Syr, syrosingopine; BPQDs, black phosphorus quantum dots; ODMSNs, openwork@ dendritic mesoporous silica nanoparticles; LOX, lactate oxidase; AAV2, adeno-associated virus serotype 2; MSN, mesoporous silica nanoparticles; TCPP, tetrakis (4-carboxyphenyl) porphyrin, 2-DG, 2-deoxy-d-glucose.

## Abbreviations

OXPHOS: mitochondrial oxidative phosphorylation
TME: tumor microenvironment
ROS: reactive oxygen species
CSCs: cancer stem cells
EPR: enhanced permeability and retention
Glut: glucose transporters
G6P: glucose-6-phosphate
HK1: hexokinase 1
PFK1: phosphofructokinase 1
F6P: fructose-6-phosphate
F1,6BP: fructose-1,6-bisphosphate
ATP: adenosine triphosphate
LDH: lactate dehydrogenase
NAD+: nicotinamide adenine dinucleotide
NADH: nicotinamide adenine dinucleotide
MCT: monocarboxylate transporters
LDHA: lactate dehydrogenase A
ETC: electron transport chain
FADH2: reduced flavin adenine dinucleotide
2DG: 2-deoxy-D-glucose
PBMCs: peripheral blood mononuclear cells
PKM2: pyruvate kinase M2
DCA: Dichloroacetate
NLR: long rod nanoparticles
NSR: short rod nanoparticles
NS: spherical nanoparticles
VLPs: virus-like particles
MSN: mesoporous silica nanoparticle
LVI: lymphatic vessel invasion
CREKA: Cys-Arg-Glu-Lys-Ala
PEG: polyethylene glycol
H2O2: hydrogen peroxide
GSH: glutathione
PFKFB3: 6phosphofructo 2kinasefructose2,6biphosphatase 3
PPP: pentose phosphate pathway
PTT: photothermal therapy
HSPs: heat shock proteins
GNR: gold nanorod
DC: diclofenac
HA: hyaluronic acid
Haase: hyaluronidase
TNBC: triple-negative breast cancer
Tregs: regulatory T cells
HBP: hexosamine biosynthetic pathway
aptPD-L1: PD-L1-antagonizing aptamers
aptCTLA-4: CTLA-4-antagonizing aptamers
DCs: dendritic cells
DMAP: damage-associated molecular patterns
PDT: photodynamic therapy
GSDMD: gasdermin D
EcN: Escherichia coli Nissle 1917
AMPK: AMP-activated protein kinase
dCTP: deoxycytidine triphosphate
SHH: sonic hedgehog pathway
SMO: smoothened
HIF-1α: hypoxia-inducible factor 1 α
GOx: Glucose oxidase
FAD: flavin adenine dinucleotide
PDAC: pancreatic ductal adenocarcinoma
CDT: chemodynamic therapy
TPZ: arapazamine
CAT: catalase
HBO: hyperbaric oxygen
MnO2: manganese dioxide
QM: quinone methide
mFe3O4: mesoporous Fe3O4
PFP: perfluorocarbon
LYTACs: Lysosome-targeting chimeras
CI-M6PR: cation-independent mannose 6-phosphate receptor
CI-M6PR: cation-independent mannose 6-phosphate receptor
ASGPR: asialoglycoprotein receptor
HCC: hepatocellular carcinoma
Trp: tryptophan
Kyn: kynurenine
IDO: 2,3-dioxygenase
MOF: metal-organic framework
1-MT: 1-methyltryptophan
PEI: polyethylenimine
·OH: hydroxyl radicals
AQ4N: banoxantrone
COX-2: cyclooxygenase-2
MeJA: jasmonate
3BP: 3bromopyruvate
PEP: phosphoenolpyruvate
JFSN: juglone-modified silver nanoparticles
NOX4: NADPH oxidase 4
OGA: O-GlcNAcase
DSF: disulfiram
SHK: shikonin
BBB: blood-brain barrier
CI: combination index
ICD: immunogenic cell death
TAMs: tumor-associated macrophages
ABC: ATP-binding cassette
DOX: doxorubicin
ICG: indocyanine green
NIR: near-infrared
TILs: tumor-infiltrating T lymphocytes
CTLA-4: cytotoxic T lymphocyte-associated protein-4
zDTL: Piezodynamic therapy
OXA: oxaliplatin
G-CSF: ranulocyte colony-stimulating factor
MDSCs: myeloid-derived suppressor cells
IL-10: interleukin-10
NK: natural killer
TNBC: triple-negative breast cancer cells
TAFs: tumor-associated fibroblasts
SLC: solute carrier
BSA: bovine serum albumin
ER: endoplasmic reticulum
Flu: fluoxetine
DEM: diethylmaleate
NO: nitric oxide
PPa: pyropheophorbide a
PLGA: poly(lactic-co-glycolic acid)
PDKs: pyruvate dehydrogenase kinases
PDC: pyruvate dehydrogenase complex
DCA: dichloroacetate
ICB: immune checkpoint blockade
OPDEA: poly[2-(N-oxide-N, N-diethylamino)ethyl methacrylate]
IL-1β: interleukin-1β
HMGB1: high mobility group box 1
PD-L1: programmed cell death-ligand 1
TPP: triphenylphosphonium
TKI: tyrosine kinase inhibitor
NSCLC: non-small cell lung cancer
HNC: head and neck cancer
CSCs: cancer stem cells
PLGA-b-PEG: poly(lactic-co-glycolic acid)-block-polyethyleneglycol
PDH: pyruvate dehydrogenase
EC: endometrial cancer
mTOR: mammalian target of rapamycin
FOXO: forkhead box protein O
CRC: colorectal cancer
ECM: extracellular matrix
CAFs: cancer-associated fibroblasts
TCA: tricarboxylic acid
IDH3α: isocitrate dehydrogenase 3 alpha
MHC: major histocompatibility complex
TGF-β: transforming growth factor-β
moDCs: monocyte-derived dendritic cells
MAPK: p38-mitogen-activated protein kinase
APCs: antigen-presenting cells
mcDCs: merocytic DCs
cDCs: conventional DCs
Th: T helper cells
TCs: cytotoxic T cells
FBP1: fructose-1,6-bisphosphatase
ECs: endothelial cells
TECs: tumor-associated endothelial cells
PHD-2: prolyl hydroxylase-2
BPQDs: black phosphorus quantum dots
RGD: Arg-Gly-Asp
LOD: lactate oxidase
Gd/CeO_2_: Gd-doped CeO_2_ nanorods
GBM: glioblastoma multiforme
DMSN: dendritic mesoporous silica nanoparticles
AAV2: adeno-associated virus serotype 2
S. oneidensis MR-1: Shewanella oneidensis MR-1
EMP: extracellular matrix proteases
SDT: sonodynamic therapy
ATO: atovaquone
Ce6: chlorin e6
RT: radiotherapy
LND: Lonidamine
